# ﻿A critical review of the distribution of the endangered European earth-borer beetle *Bolbelasmusunicornis* (Coleoptera, Geotrupidae), with new records from 13 countries and observations on its bionomy

**DOI:** 10.3897/zookeys.1105.81474

**Published:** 2022-06-15

**Authors:** Daniel Juřena

**Affiliations:** 1 Lidická 59, 796 01 Prostějov, Czech Republic Unaffiliated Prostějov Czech Republic

**Keywords:** Asia Minor, Bolboceratinae, ethology, Europe, Palaearctic realm, zoogeography

## Abstract

The distribution of *Bolbelasmusunicornis* (Schrank, 1789) is critically reviewed throughout its range with emphasis on the Czech Republic and Slovakia. The species has been reliably recorded from 377 localities in 19 countries. New records are given from 152 localities of Bulgaria, Czech Republic, Germany, Hungary, Italy, Moldova, Poland, Romania, Serbia, Slovakia, Turkey, and Ukraine. For Germany, the species is recorded for the first time in 54 years. The occurrence of the species in Switzerland is confirmed by two historical specimens from Zürich. The only known historical specimen labelled “Kaukasus” is given, which could originate from Russia, where this species has not been recorded before (however, confusion of the locality label cannot be ruled out). All published faunistic data from across the range are presented here in full, in several cases supplemented by details subsequently obtained by the author. Distribution maps are compiled separately for the Czech Republic and Slovakia, and for the entire range. A separate map is also available for Hungary, where approximately one-third of the known localities are located. Statistical data concerning the flight activity of adults, seasonal dynamics for part of the distribution area, details of records and notes on the bionomy and ethology of the species are provided. Possible feeding strategies for adults and larvae of *B.unicornis* are discussed, as well as current knowledge of the natural history of various representatives of the subfamily Bolboceratinae. A monitoring method for the species is proposed.

## ﻿Introduction

**Figure 1.**
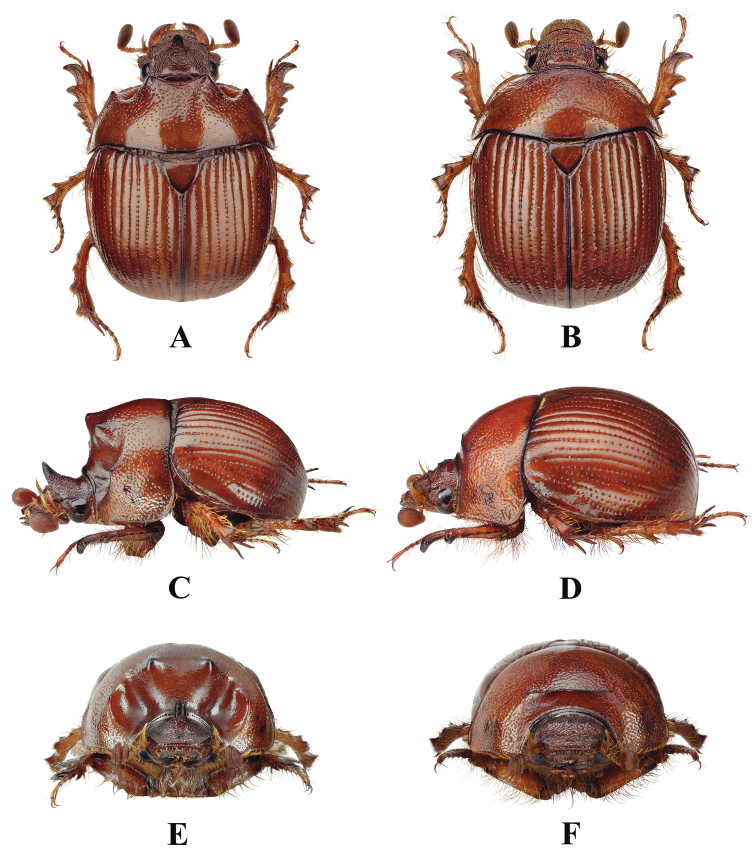
Habitus of *Bolbelasmusunicornis***A** male, dorsal view **B** female dorsal view **C** male lateral view **D** female lateral view **E** male frontal view **F** female frontal view (photographs by Peter Kurina).

**Figure 2.**
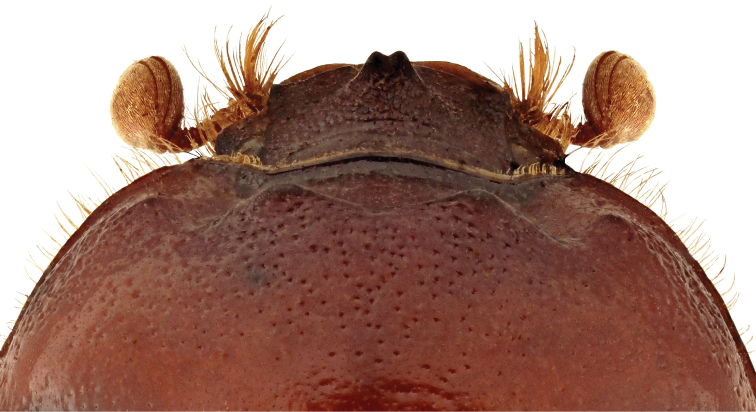
Habitus of *B.unicornis*, male, detail. Rarely, males have a frontal horn ending in two apices (photographed specimen: “Autriche” [= Austria], “coll. Reiber”, deposited in RBIN, photograph by Julien Lalanne, edited by Peter Kurina).

**Figure 3.**
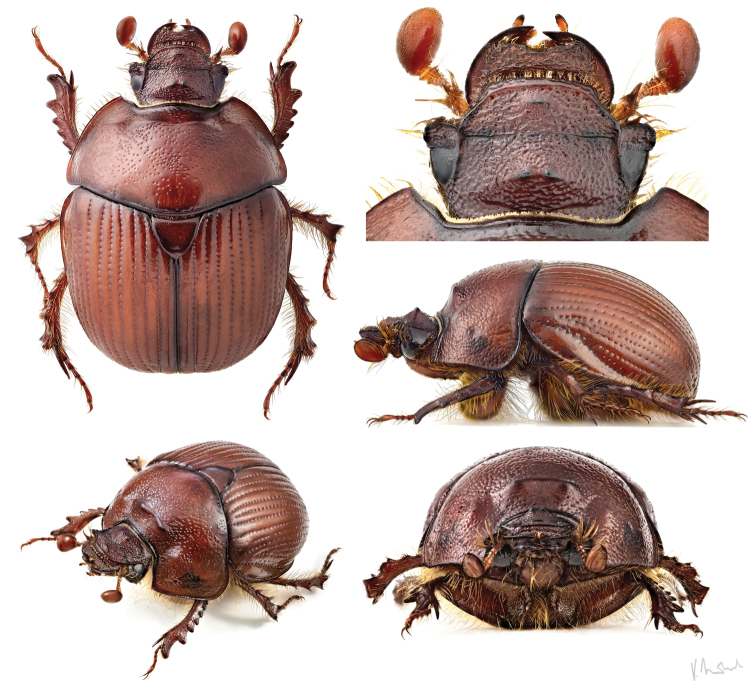
Small male of *B.unicornis* (body length: 11.5 mm) with feebly developed modifications of head and pronotum (Slovakia, Bratislava env.). The head features two small tubercles instead of the characteristic horn (photographs by Vlastimil Mihal).

*Bolbelasmusunicornis* (Schrank, 1789) is a European species of earth-borer beetle extending into the western Asian part of Turkey with the centre of distribution in the Pannonian Basin (see Faunistic records and Fig. 18). It is a medium-sized bolboce­ratine which was the subject of considerable interest to the insect collectors as early as the 19^th^ century because of its interesting and attractive appearance (for male and female habitus see Figs 1–3, 21). In literature, the body length of adults is reported to be 12.0–15.0 mm (Savchenko 1938; Endrődi 1956; Panin 1957; Mikšić 1958, 1960; Medvedev 1965; Machatschke 1969; Baraud 1992; Hůrka 2005; Ballerio et al. 2014). According to measurements taken during this study on ca. 800 specimens, the body length of this species ranges between 9.5–14.5 mm. *Bolbelasmusunicornis* is considered an endangered species with bioindicator significance throughout its range (see Habitat preferences). For this reason, it has been listed as a species of special conservation in many European countries. At the instigation of Slovakia, it has been included in Annexes II and IV of the Habitat Directive of the European Union (species in need of strict protection). As very few faunistic records are known from most countries, each new record is critically important to increase our knowledge to implement appropriate conservation strategies for the species. For more than 50 years the species has not been recorded in France, Slovenia, Bosnia and Herzegovina, Albania, and Moldova. It is probably extinct in France, Switzerland, Poland, and the Czech Republic.

The species was described as *Scarabaeusunicornu* by Schrank von Paula (1789) and subsequently as *S.aeneas* by Panzer (1793a). Since the end of the 18^th^ century, the species was often confused with *Scarabaeusquadridens* Fabricius, 1781 from India and later synonymised with it (Panzer 1793b, 1795, 1802; Illiger 1798; Duftschmid 1805; Sturm 1805, 1843; Schönherr 1806; Skrimshire 1812; Dejean 1821, 1833, 1836; Curtis 1829a, b, 1837; Stephens 1829, 1830, 1839; Eichwald 1830; Laporte de Castelnau 1840; Heer 1841). However, Illiger (1800) had already assumed that these were two distinct species. It was only Klug (1843) who separated the two species from each other, however, later authors (e.g., Erichson 1847; Gaubil 1849; Kiesenwetter and Schaum 1849; Redtenbacher 1849, 1858, 1874; Westwood 1852; Oechsner 1854; Lacordaire 1856; Calwer 1858; Fuss 1858; Gerstaecker 1863; Stierlin and Gautard 1867; Gemminger and Harold 1869; Mulsant and Rey 1871; Bertolini 1872; Jäger 1884; Seidlitz 1891; Luigioni 1929) continued to list the name *quadridens* among synonyms and often ascribed authorship of this species name to Panzer (1795). The same mistake was reported in both editions of the Catalogue of Palearctic Coleoptera (Král et al. 2006; Nikolajev et al. 2016).

Given its secretive lifestyle and lack of knowledge of effective collecting methods, the distribution and bionomy of *B.unicornis* are poorly known. Adults spend most of their time underground, with above-ground activity limited to short flight periods when they fly very close to the ground just after sunset (see Natural history of Bolboceratinae in this study). Nothing is known about the immature stages and the diet of adults and larvae. However, some authors assumed that both adults and larvae feed on hypogeous fungi (e.g., Sajó 1910a, b; Ohaus 1929; Roubal 1936; Koch 1989; Bratek et al. 1992; Merkl 2003, 2014, 2015; Nádai 2006). Adults, like in other members of the genus *Bolbelasmus*, are able to stridulate loudly, a fact first mentioned by Ghiliani (1847). Individuals of *B.unicornis* produce a wide range of sounds, varying in intensity and other characte­ristics depending on whether it is in response to a disturbance or part of their normal activities (pers. obs.). In the congeneric species *B.gallicus* (Mulsant, 1842) and *B.brancoi* Hillert & Král, 2016, this ability has also been recorded in larvae (Verdú et al. 1998: *B.brancoi* listed as *B.bocchus* (Erichson, 1841); Verdú et al. 2004; Rahola Fabra 2004).

## ﻿Materials and methods

The nomenclature used in this research follows Howden et al. (2007), Smith (2009), and Nikolajev et al. (2016), with corrections according to Bouchard and Bousquet (2020) and Ziani et al. (2021). The taxon *Bolbocerodema* Nikolajev, 1973 is considered here to be a subgenus of the genus *Bolbocerosoma* Schaeffer, 1906, in accordance with Krikken (1979) and Smith (2009). The concept of Bolboceratinae as a subfamily of Geotrupidae is consistent with Lawrence and Newton (1995), Verdú et al. (2004), Howden et al. (2007), and Nikolajev et al. (2016).

Faunistic records from the Czech Republic and Slovakia are divided into paragraphs beginning with a number representing the code of the faunistic square that refers to the Central European grid for mapping fauna and flora (Fig. 9; also see e.g., Zelený 1972; Novák 1989; Pruner and Míka 1996; Kolouch 2002). For other countries, the records are divided into paragraphs according to the largest superior administrative units or traditional regions. The countries, the faunistic square codes and the administrative units/traditional regions are ordered according to their geographical positions from east to west and from north to south. A question mark at the beginning of a faunistic record indicates dubious data. For protected areas in the Czech Republic and Slovakia, three acronyms are used in the text: PP – Přírodní památka (= Natural Monument), PR – Přírodní rezervace (= Nature Reserve), and **NPR** – Národní přírodní rezervace (= National Nature Reserve). The abbreviation **FSLG** means flying slowly low above the ground. The following acronyms are used for time zones: CEST – Central European Summer Time, and EEST – Eastern European Summer Time. The abbreviation representing a collector/observer (see list below) with no further details mentioned means the collector and depository are identical (leg. and coll.). All details regarding observations of adults of *B.unicornis* (in particular their flight activities) were provided by the listed participants of these observations. The material has been identified by the author, the curators of the collections, or the observers and collectors listed.

The following systems are used to transliterate cited literature and geographical or personal names in the Cyrillic and Armenian scripts: BGN/PCGN 2013 Agreement for Bulgarian, BGN/PCGN 1947 System for Russian, BGN/PCGN 2005 Agreement for Serbian, BGN/PCGN 2019 Agreement for Ukrainian, and BGN/PCGN 1981 System for Armenian.

For the distribution map of the Czech Republic and Slovakia, the records are divided into three time periods: the records before 1960, records between 1960–1999, and records after 1999 (Fig. 9). This map was compiled by manually placing the circles in the grid map used for faunistic research in these countries in standard free graphics software. For the distribution maps of Hungary and Europe, the following time periods are used: records before 1950, records between 1950–1999, and records after 1999 (Figs 12, 18). These maps were created using the Google Maps web application by inserting specific GPS coordinates into the system. GPS coordinates were obtained from collectors or providers of the sightings listed for each faunistic record. In cases where the exact GPS coordinates were not known (e.g., records from literature), the midpoint GPS coordinates of the village, town, county, or area were used.

Statistics on flights of adults were compiled for eight localities (seven Slovak and one Serbian), for which detailed data were available (Tables 1–8). A table with the same statistics was also created for the published data from the Italian locality of Cor­denons (Table 9; Glerean and Stefani 2019).

The graph of seasonal dynamics was generated with data obtained from countries of the Pannonian Basin for which data on a minimum of 30 specimens were available (Fig. 19).

The dates of Panzer’s works are adopted from Bousquet (2016) and Alonso-Zarazaga and Evenhuis (2017). Panzer (1793a) is cited according to Sherborn (1902), Hillert et al. (2016) and Löbl and Löbl (2016). Kuthy’s book (1898) is cited following Bousquet (2016), but with some modifications.

### ﻿Acronyms for the collectors, observers, and institutes

**ABC** Attila Balázs, Čamovce, Slovakia

**ABZ** Andrii Ivanovych Bachynskyi (Андрій Іванович Бачинський), Zalishchyky, Ukraine

**ADW** Alexander Dostal, Vienna, Austria

**AGB** András Gór, Biatorbágy, Hungary

**AHB** Adam Hergovits, Bratislava, Slovakia

**AKB** Attila Kotán, Budapest, Hungary

**AMK** András Máté, Kecskemét, Hungary

**APC** Alexandru-Mihai Pintilioaie, Comănești, Romania

**APE** Attila Pál, Érd, Hungary

**APO** Antonín Peutlschmid, Olomouc, Czech Republic

**ARC** Adrian Ruicănescu, Cluj-Napoca, Romania

**ASH** Aleš Sedláček, Hranice, Czech Republic

**ASK** Artur Anatoliiovych Shekhovtsov (Артур Анатолійович Шеховцов), Kharkiv, Ukraine

**AUP** Ákos Uherkovich, Pécs, Hungary

**BBO** Boris Bubeník Sr., Ostrava, Czech Republic

**BCK** Csaba Bán, Kecskemét, Hungary

**BJN** Jiří Brestovanský Jr., Neratovice, Czech Republic

**BJO** Boris Bubeník Jr., Ostrava, Czech Republic

**BKL** Bence Krajcsovszky, Lábatlan, Hungary

**BMP** Marek Bunalski, Poznań, Poland

**BSP** † Svatopluk Bílý, Prague, Czech Republic

**BVK** Bohdan Mykolaiovych Vasko (Богдан Миколайович Васько), Kyiv, Ukraine

**CBE** Csaba Bartha, Eger, Hungary

**CBK** Csaba Bíró, Kecskemét, Hungary

**CKZ** Csaba Kutasi, Zirc, Hungary

**CMI** Cosmin-Ovidiu Manci, Iași, Romania

**CSB** Csaba Szabóky, Budapest, Hungary

**CSS** Csaba Szinetár, Szombathely, Hungary

**CVK** Csaba Vadász, Kecskemét, Hungary

**CWP** Christian Wieser, Pischeldorf, Austria

**DCO** Dan Čagánek, Otrokovice, Czech Republic

**DHH** David Hrebeň, Havířov, Czech Republic

**DHP** David Horák, Prostějov, Czech Republic

**DJP** Daniel Juřena, Prostějov, Czech Republic

**DKC** Denis Keith, Chartres, France

**DKP** David Král, Prague, Czech Republic

**DPB** Dragan Pavićević (Драган Павићевић), Belgrade, Serbia

**DPS** Dmytro Protopopov (Дмитро Протопопов), Semyhiria, Ukraine

**DRW** Dominik Rabl, Vienna, Austria

**DVB** Dalibor Všianský, Brno, Czech Republic

**DVH** Dušan Vacík, Hranice, Czech Republic

**DVZ** Daniel Vít, Zlín, Czech Republic

**EJB** Eduard Jendek, Bratislava, Slovakia

**FKD** Ferenc Klecska, Dunaharaszti, Hungary

**FPT** Filip Pavel, Týniště nad Orlicí, Czech Republic

**FSB** Filip Štrba, Bratislava, Slovakia

**FSP** František Štěpánek, Přerov, Czech Republic

**FTK** Florian Theves, Weingarten (Baden), Germany

**FTR** Filip Trnka, Rychnov nad Kněžnou, Czech Republic

**FTV** Filip Trojan, Velké Němčice, Czech Republic

**GAB** Ádám Gór, Biatorbágy, Hungary

**GPB** Gergely Petrányi, Budapest, Hungary

**GML** Geoffrey Miessen, Liège, Belgium

**GSB** Győző Szél, Budapest, Hungary

**HDO** Hryhorii Mykolaiovych Demydov (Григорій Миколайович Демидов), Odessa, Ukraine

**HMS** Heinz Mitter, Steyr, Austria

**HTR** Hennadii Tarasenko (Геннадій Тарасенко), Rzhyshchiv, Ukraine

**IIB** Ionuț Ștefan Iorgu, Bucharest, Romania

**IJN** Ivo Jeniš, Náklo, Czech Republic

**IMO** Ivo Martinů, Olomouc, Czech Republic

**IMP** Ivan Marvan, Pardubice, Czech Republic

**IPO** Ivan Paloušek, Olomouc, Czech Republic

**IRB** Imre Retezár, Budapest, Hungary

**ITV** Ilja Trojan, Velké Němčice, Czech Republic

**JAH** † Josef Adámek, Hradec Králové, Czech Republic

**JBB** Jan Bezděk, Brno, Czech Republic

**JCM** Josef Chybík, Modřice, Czech Republic

**JDB** János Dobos, Budapest, Hungary

**JDC** Jiří Dvořák, Čelčice, Czech Republic

**JHH** Jiří Hájek, Hrobce, Czech Republic

**JHL** Jan Helešic, Lužice (near Hodonín), Czech Republic

**JHM** Jaromír Hanuš, Moravské Budějovice, Czech Republic

**JHP** Jiří Hanzlík, Přerov–Popovice, Czech Republic

**JJP** † Josef Jurčíček, Prague, Czech Republic

**JKB** Ján Kodada, Bratislava, Slovakia

**JKJ** Jaroslav Kaláb, Jinačovice, Czech Republic

**JKO** Jindřich Kuja, Olomouc, Czech Republic

**JKP** Josef Krošlák, Plzeň, Czech Republic

**JKV** Josef Kadlec, Varnsdorf, Czech Republic

**JMB** József Muskovits, Budapest, Hungary

**JMD** † Jaroslav Máslo, Dolní Radechová, Czech Republic

**JMH** Jan Matějíček, Hradec Králové, Czech Republic

**JPH** Jan Pelikán, Hradec Králové, Czech Republic

**JPP** Jiří Plecháč, Pecka, Czech Republic

**JRC** Jindřich Ryšavý Jun., České Budějovice, Czech Republic

**JRS** Jaroslav Resl, Sněžné (near Nové Město nad Metují), Czech Republic

**JSP** Jan Schneider, Prague, Czech Republic

**JST** József Sár, Teklafalu, Hungary

**JSU** Jiří Spružina, Ústí nad Labem, Czech Republic

**JTK** Jaroslav Turna, Kostelec na Hané, Czech Republic

**JVP** † Jan Vitner, Prague, Czech Republic

**JZJ** Jaroslav Žák, Jezernice, Czech Republic

**KBB** Karol Bucsek, Bratislava, Slovakia

**KDO** Karel Doležel, Olomouc, Czech Republic

**KFW** Katrin Fuchs, Vienna, Austria

**KHE** Krisztián Harmos, Eger, Hungary

**KLP** Jiří Klícha, Prague, Czech Republic

**KPH** Karel Pils, Hlohovec, Slovakia

**KPL** Krzysztof Pałka, Lublin, Poland

**KPV** † Karel Poláček, Vysoké Mýto, Czech Republic

**KRU** Květoslav Resl, Uherský Brod, Czech Republic

**KVB** Klára Varga Bánné, Kecskemét, Hungary

**KVM** Kirill Vladimirovich Makarov (Кирилл Владимирович Макаров), Moscow, Russia

**KVS** Václav Křivan, Štěměchy, Czech Republic

**LAB** László Ádám, Budapest, Hungary

**LEN** Ladislav Ernest, Nymburk, Czech Republic

**LFI** Lucian Fusu, Iași, Romania

**LHI** Lucian Hănceanu, Iași, Romania

**LKK** Ladislav Kandrnál, Kunovice (near Uherské Hradiště), Czech Republic

**LKM** Ľudevít Kašovský, Martin, Slovakia

**LMB** Levente Erik Moharos, Budakeszi, Hungary

**LMN** Ladislav Miško, Nové Zámky, Slovakia

**LMO** Luboš Mazal, Olomouc, Czech Republic

**LMT** Ladislav Mencl, Týnec nad Labem, Czech Republic

**LNB** László Nádai, Budapest, Hungary

**LRL** Luciano Ragozzino Lerma, Italy

**MBO** Michal Bednařík, Olomouc, Czech Republic

**MBF** Marco Bastianini, Follonica, Italy

**MBP** Milan Brabec, Prague, Czech Republic

**MHP** Marcel Hájek, Plzeň, Czech Republic

**MJR** Martin Jagelka, Rohožník, Slovakia

**MKJ** Martin Kejzlar, Jevíčko, Czech Republic

**MKY** Mark Yuriyevich’ K’alashyan (Մարկ Յուրիևիչ Քալաշյան), Yerevan, Armenia

**MLB** Márk Lukátsi, Budapest, Hungary

**MLS** Miloš Lošťák, Šumperk, Czech Republic

**MNB** Martin Němec, Brno, Czech Republic

**MNR** Milan Nikodým, Roztoky (near Prague), Czech Republic

**MOB** Oto Majzlan, Bratislava, Slovakia

**MPK** Michal Pikner, Kněžpole (near Uherské Hradiště), Czech Republic

**MPN** Miloš Popović (Милош Поповић), Niš, Serbia

**MPP** Milan Sláma, Prague, Czech Republic

**MPV** Milan Pilarčík, Velké Pavlovice, Czech Republic

**MRM** Miklós Ringler, Munich, Germany

**MRV** Milan Ryšavý, Vlkoš (near Přerov), Czech Republic

**MSB** Milan Štrba, Bratislava, Slovakia

**MSN** Marko Šćiban (Марко Шћибан), Novi Sad, Serbia

**MSZ** Miloslav Šanda, Žatec, Czech Republic

**MTM** Maximilian Teodorescu, Măgurele, Romania

**MTS** Milan Trubačík, Staré Město (near Uherské Hradiště), Czech Republic

**MVP** Martin Volf, Prague, Czech Republic

**MYP** Vladislav Malý, Prague, Czech Republic

**MZB** Miroslav Zúber, Bradlec, Czech Republic

**MZK** Miroslav Ihorovych Zaika (Мирослав Ігорович Заїка), Kyiv, Ukraine

**MZP** Zdena Martinová & Zdeněk Znamenáček, Prague, Czech Republic

**NKB** Naďa Kazdová, Brno, Czech Republic

**NPB** Norbert Pometkó, Budapest, Hungary

**OHS** Oliver Hillert, Schöneiche bei Berlin, Germany

**OKO** Oleksandr Oleksandrovych Kolomeichuk (Олександр Олександрович Коломейчук), Odessa, Ukraine

**OMB** † Ottó Merkl, Budapest, Hungary

**OSD** Oleksandr Oleksiiovych Sukhenko (Олександр Олексійович Сухенко), Dnipro, Ukraine

**OSO** Ondřej Sabol, Ostrava–Nová Bělá, Czech Republic

**OVK** Oleksii Vasyliuk (Олексій Василюк), Kyiv, Ukraine

**PCB** Petr Čechovský, Brno, Czech Republic

**PFS** Péter Farkas, Ságvár, Hungary

**PIL** Pavel Imríšek, Louny, Czech Republic

**PJH** Josef Pavlas, Havířov, Czech Republic

**PJL** Pavel Jáchymek, Luhačovice, Czech Republic

**PKB** Péter Kovács, Budapest, Hungary

**PKG** Peter Kurina, Gajary, Slovakia

**PMB** Peter Mihálik, Bratislava, Slovakia

**PPB** Petr Pacholátko, Brno, Czech Republic

**PSZ** Petr Středulínský, Zlín, Czech Republic

**PVP** Petr Včelička, Prague, Czech Republic

**PZW** Petr Zábranský, Vienna, Austria

**RCP** † Radek Červenka, Prague, Czech Republic

**RCR** Roman Cséfalvay, Rohovce–Kyselica, Slovakia

**REE** Róbert Enyedi, Eger, Hungary

**RFO** † Rostislav Fornůsek, Olomouc, Czech Republic

**RGM** Rudolf Gabzdil, Michalovce, Slovakia

**RHB** Roman Hergovits, Bratislava, Slovakia

**RHK** Rostyslav Pavlovych Herasymov (Ростислав Павлович Герасимов), Kyiv, Ukraine

**RKP** Radim Klíč, Prostějov, Czech Republic

**RMU** Robert Machálek, Uherské Hradiště, Czech Republic

**RPM** Riccardo Pittino, Milan, Italy

**RSG** Roland Štefanovič, Galanta, Slovakia

**RSP** Radim Šigut, Paskov, Czech Republic

**RVO** † Radovan Veigler, Olomouc, Czech Republic

**RZJ** Renata Žáková, Jezernice, Czech Republic

**SBP** Sándor Bérces, Pomáz, Hungary

**SBS** Stoyan Beshkov, Sofia, Bulgaria

**SDP** Stanislav Doležal, Plzeň–Božkov, Czech Republic

**SIB** Sándor Ilniczky, Budapest, Hungary

**SKP** Jiří Skýpala, Prague, Czech Republic

**SPP** Serge Peslier, Perpignan, France

**SRB** Richard Schnürmacher, Bratislava, Slovakia

**SRL** Stefan Rabl, Lengenfeld, Austria

**STK** Serhii Vasylovych Tsykal (Сергій Васильович Цикал), Kyiv, Ukraine

**SZM** Stefano Ziani, Meldola, Italy

**TBK** Torsten Bittner, Weißenberg, Germany

**TDB** Tamás Deli, Békéscsaba, Hungary

**TDS** Tibor Danyik, Szarvas, Hungary

**TGK** Tomáš Grulich, Dolní Studénky–Králec, Czech Republic

**TKB** † Tibor Kovács Sr., Bátonyterenye–Kisterenye, Hungary

**TKG** Tibor Kovács Jr., Gyöngyös, Hungary

**TKH** Tomáš Kopecký, Hradec Králové, Czech Republic

**TKK** Tamás Kiss, Kecskemét, Hungary

**TNB** Tamás Németh, Gödöllő, Hungary

**TSH** Tibor Spevár, Hlohovec, Slovakia

**TVP** Tomáš Vendl, Prague, Czech Republic

**VDP** Václav Dongres, Plzeň, Czech Republic

**VGG** † Vadim Gennad’yevich Grachëv (Вадим Геннадьевич Грачёв), Moscow, Russia

**VJP** Vojtěch Jiříček, Prostějov, Czech Republic

**VKK** Vitalii Volodymyrovych Kavurka (Віталій Володимирович Кавурка), Kyiv, Ukraine

**VKS** Vítězslav Kubáň, Šlapanice, Czech Republic

**VLP** Jaromír Vališ, Přerov, Czech Republic

**VMP** Vlastimil Mihal, Přerov, Czech Republic

**VPB** Vilmos Polonyi, Budapest, Hungary

**VRH** Vladislav Řebíček, Hradištko, Czech Republic

**VSI** Valentin Szénási, Isaszeg, Hungary

**VSK** Vasyl Musiiovych Serhiienko (Василь Мусійович Сергієнко), Kyiv, Ukraine

**VSM** Viktor Viktorovych Strenada (Віктор Вікторович Стренада), Mykolaiv, Ukraine

**VTH** Vladimír Thomka, Humenné, Slovakia

**VTO** Viacheslav Anatoliiovych Trach (Вячеслав Анатолійович Трач), Odessa, Ukraine

**VUB** Viorel Ungureanu, Buzău, Romania

**VVO** Vladimír Vyhnálek, Olomouc, Czech Republic

**VZO** Vladimír Zeman, Olomouc, Czech Republic

**WBW** Wolfgang Barries, Vienna, Austria

**WHS** Walter Heinz, Schwanfeld, Germany

**YHS** Yurii Mykolaiovych Geriak (Юрій Миколайович Ґеряк), Sambir, Ukraine

**YKL** Yurii Vasylovych Kanarskyi (Юрій Васильович Канарський), Lviv, Ukraine

**YKO** Yevhenii Vitaliiovych Khalaim (Євгеній Віталійович Халаїм), Odessa, Ukraine

**YSK** Yurii Yevhenovych Skrylnyk (Юрій Євгенович Скрильник), Kharkiv, Ukraine

**ZBB** Zoran Božović (Зоран Божовић), Batajnica, Serbia

**ZBP** Zsófia Mocskonyi Bércesné, Pomáz, Hungary

**ZCP** Zdeněk Čermák, Prostějov, Czech Republic

**ZDP** † Zdeněk Doležal, Plzeň, Czech Republic

**ZKB** Zoltán Körmendy, Budapest, Hungary

**ZKM** Zdeněk Kraus, Mikulovice (near Znojmo), Czech Republic

**ZLB** Zdeněk Laštůvka, Brno, Czech Republic

**ZVP** Zdeněk Vancl, Police nad Metují, Czech Republic

**BNMS** Brukenthal National Museum, Sibiu, Romania

**BZLA** Biologiezentrum Linz, Austria

**CMZC**Croatian Natural History Museum, Zagreb, Croatia

**CUIR** Alexandru Ioan Cuza University, Iași, Romania

**ETHZ** Entomological collection of the Swiss Federal Institute of Technology, Zürich, Switzerland

**FGBI** Franziskaner Gymnasium Bozen, Bolzano, Italy

**FMNH**Finnish Museum of Natural History LUOMUS, University of Helsinki, Helsinki, Finland

**GANM** “Grigore Antipa” National Museum of Natural History, Bucharest, Romania

**GUNU** Nizhyn Gogol State University, Nizhyn, Ukraine

**HNHM**Hungarian Natural History Museum, Budapest, Hungary

**IECA**Institute of Entomology, Biology Centre of the Czech Academy of Sciences, České Budějovice, Czech Republic

**IZCM**Institute of Zoology of the Academy of Sciences of Moldova, Chișinău, Republic of Moldova

**JHIS** Jovan Hadži Institute of Biology of the Research Centre of the Slovenian Academy of Sciences and Arts, Ig, Slovenia

**LKKA** Landesmuseums für Kärnten, Klagenfurt am Wörthersee, Austria

**MCAS**Museo Civico Archeologico e di Scienze Naturali “Federico Eusebio", Alba, Italy

**MCZR**Museo Civico di Zoologia, Rome, Italy

**MFSN**Museo Friulano di Storia Naturale, Udine, Italy

**MHKC** Museum of Eastern Bohemia in Hradec Králové, Hradec Králové, Czech Republic

**MHNG**Muséum d’histoire naturelle de Genève, Geneva, Switzerland

**MIZP**Museum and Institute of Zoology of the Polish Academy of Sciences, Warsaw, Poland

**MJMC** Muzeum jihovýchodní Moravy ve Zlíně, Zlín, Czech Republic

**MKPC** Muzeum Komenského v Přerově, Přerov, Czech Republic

**MMBC**Moravian Museum, Brno, Czech Republic

**MNBG** Leibniz-Institut für Evolutions- und Biodiversitätsforschung, Museum für Naturkunde, Berlin, Germany

**MMGH** Mátra Museum of the Hungarian Natural History Museum, Gyöngyös, Hungary

**MMSH**Móra Ferenc Museum, Szeged, Hungary

**MNFI** Natural History Museum “La Specola”, Florence, Italy

**MNHN**Muséum national d’Histoire naturelle, Paris, France

**MNSA** Museum Niederösterreich, Sankt Pölten, Austria

**MPGU** Moscow Pedagogical State University, Moscow, Russia

**MSNB** Museo di Scienze Naturali dell’Alto Adige, Bolzano, Italy

**MSNG**Museo Civico di Storia Naturale “Giacomo Doria”, Genoa, Italy

**MSNM**Museo Civico di Storia Naturale, Milan, Italy

**MTDG** Senckenberg Naturhistorische Sammlungen, Museum für Tierkunde, Dresden, Germany

**MUSE**Museo delle Scienze, Trento, Italy

**MZLU**Biological Museum, Lund University, Lund, Sweden

**MZSF**Musée zoologique de l’université et de la ville de Strasbourg, Strasbourg, France

**NHMB**Naturhistorisches Museum Basel, Switzerland

**NHMD**Natural History Museum of Denmark, University of Copenhagen, Copenhagen, Denmark

**NHMK** State Natural History Museum of V. N. Karazin Kharkiv National University, Kharkiv, Ukraine

**NHML**Natural History Museum, London, United Kingdom

**NHMU** National Science and Natural History Museum of the National Academy of Sciences of Ukraine, Kyiv, Ukraine

**NHMW**Naturhistorisches Museum Wien, Vienna, Austria

**NMAG**Naturmuseum Augsburg, Germany

**NMBE**Naturhistorisches Museum Bern, Switzerland

**NMCM** National Museum of Ethnography and Natural History, Chișinău, Republic of Moldova

**NMEG**Naturkundemuseum Erfurt, Germany

**NMPC**National Museum, Prague, Czech Republic

**NMSB**National Museum of Natural History, Sofia, Bulgaria

**PMSL**Slovenian Museum of Natural History, Ljubljana, Slovenia

**RBIN**Royal Belgian Institute of Natural Sciences, Brussels, Belgium

**RMNH** Naturalis Biodiversity Centre (formerly Rijksmuseum van Natuurlijke Historie), Leiden, Netherlands

**SDEI**Senckenberg Deutsches Entomologisches Institut, Müncheberg, Germany

**SIZK**I. I. Schmalhausen Institute of Zoology of National Academy of Sciences of Ukraine, Kyiv, Ukraine

**SMLU** State Museum of Natural History, Lviv, Ukraine

**SMNK**Staatliches Museum für Naturkunde Karlsruhe, Germany

**SMNS**Staatliches Museum für Naturkunde Stuttgart, Germany

**SMOC**Silesian Museum, Opava, Czech Republic

**SNMS** Slovak National Museum–Natural History Museum, Bratislava, Slovakia

**TLMF**Tiroler Landesmuseum Ferdinandeum, Innsbruck, Austria

**TMLS** Tekovské múzeum v Leviciach, Levice, Slovakia

**UMJG**Universalmuseum Joanneum, Graz, Austria

**VMHS** Vihorlatské múzeum Humenné, Slovakia

**ZFMK**Zoologishes Forschungsmuseum Alexander Koenig, Bonn, Germany

**ZMNU** Zoological Museum of the Taras Shevchenko National University, Kyiv, Ukraine

**ZMPC** Západočeské muzeum v Plzni, Plzeň, Czech Republic

**ZINR** Zoological Institute of Russian Academy of Sciences, Saint Petersburg, Russia

**ZSMG**Staatliche Naturwissenschaftliche Sammlungen Bayerns, Zoologische Staatssammlung, Munich, Germany

**ZUDH** Department of Nature Conservation, Zoology and Game Management, University of Debrecen, Debrecen, Hungary

## ﻿Systematics

**Family: GEOTRUPIDAE Latreille, 1802**

**Subfamily: Bolboceratinae Mulsant, 1842**

**Tribe: BOLBELASMINI Iablokoff-Khnzorian, 1977**

**Genus: *Bolbelasmus* Boucomont, 1911**

**Subgenus: *Bolbelasmus* Boucomont, 1911**

**Species: *Bolbelasmus (Bolbelasmus) unicornis* (Schrank, 1789)**

## ﻿Faunistic records

### ﻿Czech Republic

**Published data**

? **5354**: “Kummer” [= Hradčany near Mimoň], 1 ♂ flying in the evening, no other data (Kral 1915). Given that Kral listed several species from this locality which have never been confirmed, this record is not considered very reliable.

**5756**: Loučeň, 28.v.1905, 1 spec., [Augustin] Šrámek leg., Radek Červenka and Radek Dunda det., coll. NMPC (Juřena et al. 2008); note: this specimen was probably stolen from NMPC.

**6865**: “Kammberg b. Brünn” [probably Brno – Kohoutovice env., perhaps Kamenný vrch hill], no other data (Horion 1958). This specimen should be deposited in Georg Frey’s collection in NHMB, but still on loan (Christoph Germann pers. comm., 2021).

**7067**: Hovorany, 6.v.1941, Jan Roubal leg. (Tesař 1957); Čejč, 28.v.1982, 1 ♀, J. Voříšek leg. (Juřena et al. 2008); Čejč env., Bílý kopec hill, [= PP Bílý kopec u Čejče, ca. 48°56'14"N, 16°59'E, ca. 200 m a.s.l.], July 1978, collector not specified (Juřena et al. 2008); Čejč env., “Mansonova step” [= “Manson’s steppe”, 48°55'32.1"N, 16°58'46.6"E], 1.vii.1987, 1 ♂, at light at 21.45 CEST, VJP (Juřena et al. 2008); 15.vi.1988, 1 ♂ FSLG at 21.45 CEST, ca. 20 °C, VJP (Juřena et al. 2008); 16.vi.1988, 1 ♂ flying ca. 10 cm above the ground at 21.52 CEST, ca. 10–12 °C, VJP leg., coll. DJP (Juřena et al. 2008); 17.–18.vi.1988, 2 ♀♀, JKJ (Juřena et al. 2008); 26.vi.1988, 1 ♀ was caught while trying to fly out from the grass, 21.55 CEST, MLS (Juřena et al. 2008); 29.vi.1999, 1 ♀ FSLG at 22.00 CEST, VKS (Juřena et al. 2008; Hillert et al. 2016); 3.vii.1999, 1 ♂ crawling on the footpath at 21.55 CEST, together with 1 ♂ of *Od.armiger*, RZJ leg., coll. JZJ (Juřena et al. 2008).

**6568**: Prostějov, [between 1878–1899, see Koleška 1985] [Karel] Kyselý leg. (Kliment 1899); Záhoří near Prostějov [probably area SW of the town, near the village of Domamyslice (6568), or Na Záhoří hill (6468), ca. 600 m NE of the village of Čelechovice na Hané – Kaple], [probably between 1878–1899], [Karel] Kyselý leg. (Fleischer 1930); “Prosznitz” [= Prostějov] (Schubert 1905; Zoufal 1922; Horion 1958); “Prossnitz” [= Prostějov], 1 ♂, [probably between 1878–1899], K[arel] Kyselý leg., coll. Georg Frey deposited in NHMB (Hillert et al. 2016).

? **6570**: Přerov env. [probably Bochoř near Přerov], no other data (Hudeček 1928, 1930); Bochoř, no other data (Hudeček 1937). These two records are very doubtful. Rusty-coloured specimens of *Od.armiger* (ab.testaceus) labelled as *Bolbelasmusunicornis*, with black specimens of the same species, correctly labelled as *Odonteusarmiger*, have been found in the Hudeček’s collection in MKPC; no specimens of *B.unicornis* were discovered in this collection (Jaroslav Žák pers. comm., 2016).

**6870**: “Ungarisch Hradisch” [= Uherské Hradiště] env., Morava River valley, no other data (Schlögl 1883).

**Material examined and new observations**

**7067**: Bořetice env., PR Zázmoníky, 48°56'06.9"N, 16°51'20.5"E, ca. 300 m a.s.l., 1.v.1998, 1 elytron excavated from loess soil, KRU obs.; Čejč env., “Květnatá step” [= so-called Květnatá steppe, northern part of the PR Čejkovické Špidláky reserve], 48°55'22.0"N, 16°57'24.2"E, ca. 190 m a.s.l., 1.vii.1995, remains of a female excavated from a burrow of *Oryctolaguscuniculus*, KRU (VKS det., 15 October 2005); Čejč env., “Mansonova step” [= so-called “Manson’s steppe”], 48°55'32.1"N, 16°58'46.6"E, ca. 210 m a.s.l., 20.vi.1986, 1 ♀ FSLG after sunset, PCB; 17.vi.1988, 1 ♀ FSLG after sunset, PCB; 21.vi.1988, 2 ♀♀ FSLG after sunset, VKS; 27.vi.1988, 8 spec. FSLG after sunset (for a photograph of one of them see Král et al. 2018), together with hundreds of spec. of *Odonteusarmiger* (Scopoli, 1772), VKS; 29.vi.1988, 2 spec. FSLG after sunset, VKS; 19.vi.1989, 1 ♀ FSLG after sunset, JTK; 16.vi.1995, 1 ♂ FSLG after sunset, VKS (for partial data on this record see Hillert et al. 2016); 17.vi.1995, 1 ♂ and 1 ♀ flying slowly ca. 10 cm above the ground after sunset just after the rain, VKS; Mutěnice [= Čejč env.], “Mansonka” [= Manson’s steppe], 2002, no other data [1 spec., anonymous collector leg. et coll.], non-public record of NDOP [= Records Database of Nature Conservation] of AOPK ČR [= Nature Conservation Agency of the Czech Republic].

**6376**: “Friedek Umg.” [= Frýdek-Místek env.], 20.vi.1923, 1 ♀ [ex. coll. Dr Karel Samšiňák], Jos[ef] Hlisnikowski [leg.], DJP det., coll. SMOC.

**Comment**

In the Czech Republic, the species is known from a few localities only. Old reports by Kral (1915) from Hradčany near Mimoň and by Hudeček (1928, 1930, 1937) from Přerov and Bochoř are dubious. In this study, the species is reported for the first time from northern Moravia on the basis of an old record from the vicinity of Frýdek-Místek. The latest record from the Czech Republic is from Čejč from 2004 and will be published with additional details at a later date (David Král pers. comm., 2021). For the distribution of the species in the Czech Republic see Fig. 9.

### ﻿Slovakia

**Published data**

**7867**: [Bratislava env.,] Děvín: Kobyla [= Devínska Kobyla hill], [between 1921–1936, see Koleška 1995a], 1 spec. in horse dung, [František] Šlégl leg. (Roubal 1936; Majzlan et al. 2005); “Dévény” [= Bratislava – Devín], no other data (Endrődi 1957).

**7568**: Malacky, no other data (Roubal 1938).

**7868**: “Pozsony” or “Presburg” [= Bratislava] env., no other data (Bolla 1859; Rózsay 1868, 1880; Kuthy 1898; Ortvay 1902; Balthasar 1933; Roubal 1936; Endrődi 1957); Bratislava, June 1957, 1 ♂, collector unknown, coll. LKK (Juřena et al. 2008).

**7968**: Bratislava, Kopáč Island, [PR Kopáčsky ostrov], 19.v.2006, 1 spec., Malaise trap, MOB leg. (Majzlan 2006, 2007; Juřena et al. 2008).

**7969**: “Somorja” [= Šamorín], 10.v.1897, 1 spec. inside the digestive system of *Upupaepops*, Ernő Csiki obs. (Csiki 1905).

**7371**: “Pustá Ves” [= Prašník – Horná Pustá Ves or Dolná Pustá Ves], 22.vii.1984, 1 ♀, [at light], JMD leg., coll. NMPC (Hillert et al. 2016; data completed by the author).

**7272**: Čachtice, [probably between 1920–1938, see Koleška 1981], F[rantišek] Hajný leg., coll. JDC (Juřena et al. 2008).

**7572**: Hlohovec, undated [probably first half of the 20^th^ century], 1 ♀, Várkonyi leg., coll. DKP deposited in NMPC (Juřena et al. 2008; Hillert et al. 2016).

**7373**: Brunovce, no other data, 1 ♀ in coll. NMEG (Hillert et al. 2016).

**7074**: “Liborcsa” [= Nemšová – Ľuborča], [ca. 230 m a.s.l.], undated [probably second half of the 19^th^ century], 2 spec., Nitnausz leg. (Brancsik 1899, 1905; Balthasar 1933); “Bolessó – Pjechó” [= Bolešov – Piechov], “Branne” forest, [ca. 250–300 m a.s.l.], undated, József Laczó leg. (Laczó 1905; Laco 1928); [Trenčín –] Zlatovce [env., Malá hora hill, 48°54'43"N, 18°0'30"E, ca. 230 m a.s.l. and Vinohrady, 48°54'47.22"N, 18°1'4.68"E, ca. 250 m a.s.l.], 1.vi.–10.vii.[probably 1920s], tens of spec., Rudolf Čepelák leg. (Čepelák 1925; the site specified from Čepelák’s diary – see Fig. 20); June 1926, [Rudolf] Čepelák leg., 1 ♂ in coll. JSP (Juřena et al. 2008) and 1 ♀ (ex original coll. Vladimír Balthasar) in coll. NMPC (Hillert et al. 2016; data completed by the author); Trenčín – Zlatovce [env.], 27.vi.1935, 1 ♀, collector unknown, coll. NMPC (Hillert et al. 2016); Istebník env., “Weinberg” [= Trenčín – Zlatovce env., Vinohrady, 48°54'47.22"N, 18°1'4.68"E, ca. 250 m a.s.l.], May-July 1926–1927, more spec., Georg Polentz and Rudolf Čepelák leg. (Polentz 1927).

**7174**: Trenčín, date not specified, old vineyard, more spec, Rudolf Čepelák, František Hajný, and Ladislav Korbel leg., and 1 spec., Jan Roubal leg. (Roubal 1936); Trenčín, no other data (Endrődi 1957); “Trencsen, Hungaria” [= Hungary, Trenčín], 1 ♂ and 1 ♀ with no other data, coll. BMP (Bunalski 1999; collection specified by Bunalski pers. comm., 2021); Trencsen [= Trenčín], no other data, 1 ♂ and 1 ♀ in coll. OHS (Hillert et al. 2016); Trenčín, no other data, 3 spec. coll. TMLS (Kollár and Smetana 1994); Trenčín, [Rudolf] Čepelák leg., no other data (Tesař 1954, 1957); Trenčín, no other data, 2 ♂♂ and 1 ♀ in coll. NMPC (Hillert et al. 2016; data specified by the author), 1 ♀ with no other data (Král et al. 2018); Trenčín, undated, 1 ♀, V[ilém] Steidl leg., ex original coll. Jan Havelka, currently in coll. NMPC (Hillert et al. 2016; data specified by the author); Trenčín, undated, 1 ♂ and 1 ♀, Dr A[lois] Richter leg., coll. NMPC (Hillert et al. 2016); Trenčín, undated, [Rudolf] Čepelák leg., 2 ♂♂ and 3 ♀♀ in coll. DKP deposited in NMPC, 3 ♂♂ in coll. MJMC [data specified by the author], 1 spec. in coll. JMH, 2 ♀♀ in coll. JSP, 1 spec. in coll. MZP, 9 ♂♂ and 10 ♀♀ in coll. NMPC, 2 ♂♂ and 1 ♀ in coll. OHS, 1 spec. in coll. SDP (Juřena et al. 2008; Hillert et al. 2016; data specified by the author); Trenčín, undated, [Ladislav] Korbel leg., 3 ♂♂, in coll. MJMC (Juřena et al. 2008; data specified by the author); Trenčín, May 1931, Dr A[lois] Richter leg., 1 ♂ in coll. JMH (Juřena et al. 2008), 1 ♀ in coll. NMPC (Hillert et al. 2016); Trenčín, June 1931, 1 ♀, Dr A[lois] Richter leg., coll. MJMC (Juřena et al. 2008; data specified by the author); Trenčín, 1960, no other data, 1 ♀ in coll. MZB (Juřena et al. 2008).

**7274**: “Trenčín – Inovec” [= Považský Inovec Mts, Inovec hill env.], undated [probably 1920s-1930s], 1 ♂, [Rudolf] Čepelák leg., ex original coll. Rudolf Veselý, currently in coll. NMPC (Hillert et al. 2016; data completed by the author).

**7374**: “Podhragy” [= Podhradie near Topolčany], June and July 1895–1897, collector not specified (Kelecsényi 1900; Roubal 1936).

**8174**: “Keszegfalu” [= Keszegfalva, currently Kameničná], 25.v.1906, 1 spec. inside the digestive system of *Falcovespertinus*, Ernő Csiki obs. (Csiki 1910).

**7275**: Ľutov [env., Pálenice hill, ca. 48°46'57"N, 18°16'44"E, 250–300 m a.s.l.], 1.vi.–15.vii.[probably 1920s], Čepelák leg. (Tesař 1957; the site specified from Čepelák’s diary – see Fig. 20).

**8176**: “Bátorkeszi” [= Bátorove Kosihy], June [between 1919–1923, see Koleška 1995b], 1 spec., sandy path, [Václav] Thurnher leg. (Roubal 1936; Endrődi 1957).

**8177**: Štúrovo (8278) [Štúrovo env., Belianské kopce hills, Modrý vrch hill env., PR Vŕšok env.], 24.v.1985, 2 spec., RFO (Týr 1997); Modrý vrch hill near Štúrovo [= Štúrovo env., Belianské kopce hills, Modrý vrch hill env., PR Vŕšok env.], 28.vi.1981, 1 ♂ and 1 ♀, 23.v.1985, 1 ♂, IJN (Juřena et al. 2008).

**8178**: Kamenica nad Hronom env., 47°50'29.5"N, 18°43'34.8"E, 9.vii.1980, ca. 30 spec. FSLG around midnight, PJH leg., 1 ♂ and 1 ♀ in coll. VJP, 1 ♀ in coll. ZDP deposited in ZMPC (Juřena et al. 2008; data supplemented by VJP pers. comm., 2021, and the author); 17.vii.1990, 1 ♂ and 1 ♀, 5.v.1992, 1 ♂, MTS (Juřena et al. 2008); 26.vi.1999, 1 ♂ and 1 ♀, dead on a path, JCM (Juřena et al. 2008); 24.v.2008, 2 ♀♀, at light (flew through the open window) ca. at 21.45 CEST, BBO (Juřena et al. 2008); Kamenica nad Hronom env., NPR Burdov, 47°49'32.88"N, 18°44'54.72"E, 154 m a.s.l., June 2011, 1 spec., Malaise trap, Vladimír Hošek leg. (Majzlan 2016); Kováčov, July 1985, 1 spec., ZVP (Týr 1997), 5.vii.1985, 1 spec., KPV leg., coll. JRS (Juřena et al. 2008); 4.vii.1999, 1 ♂, Karel Deneš Sr. leg., coll. DCO (Juřena et al. 2008); 29.vi.2001, 1 ♂, at light, JSU leg., coll. MSZ (Juřena et al. 2008).

**8179**: Chľaba env., 47°49'27"N, 18°50'57"E (the site near the confluence of the Danube and Ipeľ rivers), 103 m a.s.l., 5.vii.1975, plant materials alluviated by flooded Danube and Ipeľ rivers, 1 ♀, VKS leg. et coll., 1 ♂, PPB leg., coll. VKS (Juřena et al. 2008; data corrected by VKS pers. comm., 2021).

**7781**: “Plachti[n]ce” [= Horné, Stredné or Dolné Plachtince], 5.vi.1938, [Rudolf] Schwarz leg. (Tesař 1957).

**7683**: “Losoncz” [= Lučenec], 1877–1891, Emil Malesevics leg. (Malesevics 1892; Černecký et al. 2014); Lučenec, June [probably first half of the 20^th^ century], Slanec leg. (Roubal 1936).

**7884**: Šiatorská Bukovinka, parking at the cemetery, [48°11'4"N, 19°49'33"E; 290 m a.s.l.], 8.vii.1973, 1 spec., at light (kerosene lamp), SKP leg., coll. SPP (Skýpala 1978; Juřena et al. 2008; storage of the specimen specified by Serge Peslier pers. comm., 2022)

**7785**: Hajnáčka [– Buková, 48°13'36.97"N, 19°58'24.11"E, steppe slope near the forest], 15.vii.1984, 1 ♀, dead on the ground, RCP (Juřena et al. 2008); 5.–8.vi.1986, 1 ♂, JMH; 18.v.1989, 1 ♂ FSLG after sunset, IMO; 5.vi.1989, 1 ♀, in flight at 21.35 CEST, IMO; 10.–11.vi.1989, 7 ♂♂ and 2 ♀♀, in flight after sunset or crawling on the ground, RVO (Juřena et al. 2008); 11.vi.1989, 1 ♂ and 2 ♀♀ FSLG after sunset, APO (Juřena et al. 2008); 24.vi.1989, 3 ♂♂ and 2 ♀♀ FSLG after sunset, MBO (Juřena et al. 2008); 27.vi.1989, 1 ♂ and 1 ♀ FSLG after sunset, RVO (Juřena et al. 2008); 1.vii.1989, 1 ♂ and 1 ♀ excavated with a garden shovel from their burrow on a steppe in the immediate vicinity of an oak forest (the burrow with push-up was localised thanks to audible stridulation of one or both specimens), VMP (Juřena et al. 2008); 6.vii.1989, 1 ♂ and 2 ♀♀ FSLG after sunset, MBO (Juřena et al. (2008); 16.vi.1990, 1 ♀ FSLG at 21.25 CEST, VJP (Juřena et al. 2008); 17.vi.1990, 1 ♂ flying at 21.28 CEST, VJP, 2 ♀♀ flying at 21.30–22.00 CEST, MNR (Juřena et al. 2008); 28.vi.1990, 1 ♂ crawling on the ground near an oak forest at 21.30 CEST, IMO (Juřena et al. 2008); 16.vi.1991, 1 ♂ and 1 ♀ FSLG at 21.25 CEST, VJP; 16.vi.1992, 3 ♂♂ and 3 ♀♀ FSLG after sunset, JDC, VJP (Juřena et al. 2008); 18.vi.1992, 1 ♂ and 1 ♀ FSLG after sunset, JDC (Juřena et al. 2008); 16.vi.1994, 2 ♂♂ FSLG after sunset, APO (Juřena et al. 2008); 28.v.1995, 1 spec., JKP (Týr 1997), 1 ♀ flying at 21.30–22.00 CEST, MNR (Juřena et al. 2008); 3.vii.1997, 3 ♀♀ FSLG at 21.30–22.00 CEST, MZP, MNR (Juřena et al. 2008); 16.vi.2009, together with *Od.armiger* and *Och.chrysomeloides*, the number of spec. and the collector name not specified (Byk et al. 2012).

**7882**: Kiarov, 15.–20.vi.1936, 1 ♀, [Dr Rudolf] Schwarz leg., ex original coll. Bohumil Štícha, currently in coll. NMPC (Juřena et al. 2008; Hillert et al. 2016; data completed by the author).

**7277**: Prievidza, forest park, 18.vii.1995, 1 ♂, RGM; (Juřena et al. 2008).

**7280**: Banská Bystrica, 18.v.1979, 1 ♂, collector unknown, coll. KVS (Juřena et al. 2008; Hillert et al. 2016).

**7488**: Silická Brezová, 3.vi.1999, 1 ♀, dead on a path crossing a steppe meadow, KDO (Juřena et al. 2008).

**7390**: Hrhov, 20.–21.vii.1981, 10 spec. excavated from their burrows, in a few cases together with *Od.armiger* (at a depth of up to 7 cm, the burrows changed direction from vertical to horizontal; in two cases, in one hole were two males or two females of *B.unicornis* together), LMT (Juřena et al. 2008).

**7494**: Slanská Huta env., 48°34'54.8"N, 21°28'31.7"E, 600 m a.s.l., 24.vii.1972, 1 ♀ crawling on the ground after sunset, ZLB obs. + photo – see Fig. 14A (Juřena et al. 2008; data specified by ZLB pers. comm., 2022).

**7596**: Ladmovce, 9.viii.1982, 2 ♂♂ excavated from their burrows from a depth of 8 cm, and 1 ♀ from a depth of ca. 20 cm, LMT (Juřena et al. 2008).

**7097**: Lackovce env., Veľká hill, [ca. 48°56'35"N, 21°58'13.5"E], 2.vii.–31.viii.2001, 2 ♂♂ and 2 ♀♀, steep forest-steppe hillside with shrubbery of *Rosacanina* and *Prunusspinosa*, pitfall traps with formaldehyde, together with more spec. of *Od.armiger*, VTH leg., coll. VMHS (Juřena et al. 2008); 16.vii.2017, 1 spec., pitfall trap with formaldehyde (48°56'37.63"N, 21°58'14.33"E), A. Macková leg. (Gajdoš and Majzlan 2018; Majzlan 2018).

**7098–7099**: Snina, July 1965, 1 ♂, MPP leg., coll. DKP deposited in NMPC (Hillert et al. 2016).

**Material examined and new observations**

**7868**: “Pressburg” [= Bratislava], no other data, 2 ♂♂ and 3 ♀♀ in coll. UMJG; “Hu, Pressburg” [= Hungaria, Bratislava], undated, 1 ♂, Maj[or Robert] Weber [leg.], coll. UMJG.

**Figure 4.**
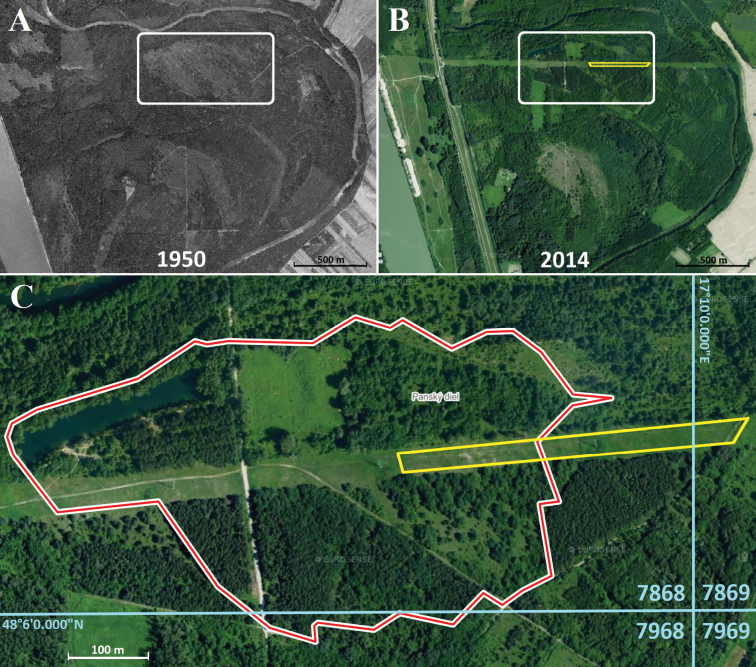
Bratislava, Kopáč Island (Slovakia), the area with the largest known population of *B.unicornis* in Europe before its conversion by inappropriate conservation management (removal of small trees and shrubs and introduction of intensive sheep grazing) **A** view of the site in 1950 **B** view of the site in 2014 with the area of the highest density of *B.unicornis* outlined with yellow borders **C** detail of the area with the highest density of the species with faunistic squares marked (see Materials and methods and Fig. 9).

**Figure 5.**
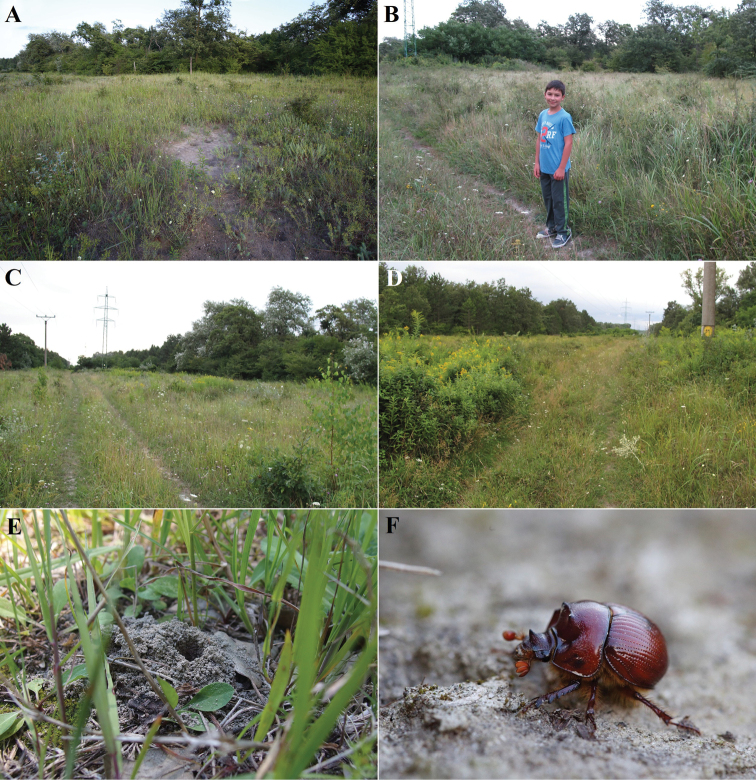
Bratislava, Kopáč Island, PP Panský diel (Slovakia) in 2016 (before its conversion due to inappropriate conservation management) **A–D** site details (**A** photographed by Dalibor Všianský **B–D** photographed by Ilja Trojan) **E** push-up of *B.unicornis* (photograph by Milan Štrba) **F** male excavated from its burrow (photograph by Dalibor Všianský).

**7868–7869**: Bratislava – Podunajské Biskupice, Kopáč Island, PP Panský diel env. (Figs 4, 5), (e.g., 48°6'4.83"N, 17°9'37.55"E; 48°6'5.7"N, 17°9'48.7"E; 48°6'6.58"N, 17°9'58.21"E; 48°6'6.77"N, 17°10'2.16"E), 132–133 m a.s.l., 31.vii.2009, 1 ♀, at UV light, KBB leg., coll. DVH; 18.viii.2014, 1 ♀, at light, KBB obs.; 20.viii.2014, 1 ♂ and 1 ♀ FSLG after sunset, AHB and RHB obs.; 27.viii.2014, 2 ♀♀ FSLG after sunset, AHB obs.; 5.ix.2014, 3 ♂♂ and 2 ♀♀ FSLG after sunset, PKG and RHB obs.; 3.vi.2015, 11 ♂♂ and 4 ♀♀ flying slowly 10–20 cm above the ground after sunset, AHB and RHB obs.; 5.vi.2015, 5 ♂♂ and 1 ♀ flying slowly 10–20 cm above the ground after sunset, PKG and RHB obs.; 7.vi.2015, 4 ♂♂ FSLG after sunset, AHB obs.; 26.viii.2015, 2 ♀♀ flying slowly ca. 0.5 m above the ground at 20.20 and 20.30 CEST, DJP obs.; 28.viii.2015, 1 ♀ flying ca. 10–20 cm above the ground at 20.27 CEST, AHB obs.; 29.v.2016, 7 spec. FSLG after sunset, together with ca. 15 spec. of *Od.armiger*, EJB, MSB and RHB obs.; 7.vi.2016, 11 spec. (4 ♂♂, 6 ♀♀ and 1 spec. not sexed) FSLG at 21.25–21.45 CEST, AHB and RHB obs. (see Table 1 for full data on the flights); 8.vi.2016, 5 spec. (4 ♂♂ and 1 spec. not sexed) flying slowly ca. 0.5 m above the ground at 21.27–21.43 CEST, together with 3 spec. of *Od.armiger*, DJP obs. (see Table 1 for full data on the flights); 18.vi.2016, 3 ♂♂ and 3 ♀♀ flying relatively slowly (but faster than 8.vi.2016) ca. 0.5 m above the ground at 21.33–21.53 CEST, DJP obs. (see Table 1 for full data on the flights); 21.vi.2016, 3 ♂♂ and 2 ♀♀ flying slowly ca. 0.5 m above the ground at 21.31–22.03 CEST, MSB obs. (see Table 1 for full data on the flights); 22.vi.2016, 2 ♂♂ and 4 ♀♀ flying slowly ca. 0.5 m above the ground at 21.21–21.57 CEST, MSB obs. (see Table 1 for full data on the flights); 23.vi.2016, 3 ♂♂ and 1 ♀ excavated from their burrows on the edge of a path crossing a forest-steppe meadow, 6 ♂♂ and 5 ♀♀ FSLG at 21.33–21.53 CEST, together with ca. 15 spec. of *Od.armiger*, DJP obs. (see Table 1 for full data on the flights); 24.vi.2016, 10 ♂♂ and 14 ♀♀ FSLG at 21.36–22.08 CEST, DJP, MSB and PKG obs. (see Table 1 for full data on the flights); 25.vi.2016, 2 ♂♂ and 5 ♀♀ FSLG at 21.28–22.08 CEST, MSB and FSB obs. (see Table 1 for full data on the flights); 26.vi.2016, 13 ♂♂ and 12 ♀♀ flying slowly up to ca. 0.5 m above the ground or relatively quickly ca. 1.5 m above ground at 21.31–22.09 CEST, together with ca. 10 spec. of *Od.armiger* and 3 spec. of *Ochodaeuschrysomeloides* (Schrank, 1781), DJP, FSB, MSB and PKG obs. (see Table 1 for full data on the flights); 27.vi.2016, 2 ♂♂ and 8 ♀♀ FSLG at 21.31–22.57 CEST, MSB and FSB obs. (see Table 1 for full data on the flights); 28.vi.2016, 2 ♂♂ and 7 ♀♀ flying slowly up to ca. 0.5 m above the ground or relatively quickly ca. 1–1.5 m above the ground at 21.28–22.48 CEST, together with 1 spec. of *Od.armiger*, DJP and PKG obs. (see Table 1 for full data on the flights); 29.vi.2016, 8 ♂♂ and 6 ♀♀ FSLG at 21.29–22.03 CEST, together with ca. 10 spec. of *Od.armiger* and 7 spec. of *Och.chrysomeloides*, DJP, PKG and MSB obs. (see Table 1 for full data on the flights); 30.vi.2016, 2 ♂♂ and 3 ♀♀ FSLG at 21.38–22.48 CEST, MSB and FSB obs.; 1.vii.2016, 1 ♀ flying slowly up to 0.5 m above the ground at 21.35 CEST, together with 1 spec. of *Od.armiger* and 1 spec. of *Och.chrysomeloides*, DJP obs.; 21.vii.2016, 25 ♂♂ and 11 ♀♀, most individuals flying slowly, some relatively quickly, up to 0.5 m above the ground, 2 spec. flying quickly ca. 1–1.5 m above the ground, at 21.09–21.51 CEST, together with more spec. of *Od.armiger* and *Och.chrysomeloides*, DJP and MSB obs. (see Table 1 for full data on the flights); 22.vii.2016, 28 ♂♂ and 23 ♀♀ flying up to 0.5 m above the ground at 21.08–21.51 CEST, together with more spec. of *Od.armiger* and *Och.chrysomeloides*, DJP and MSB obs. (see Table 1 for full data on the flights); 23.vii.2016, 26 ♂♂ and 15 ♀♀ flying up to 1 m above the ground at 21.09–21.53 CEST, together with more spec. of *Od.armiger* and *Och.chrysomeloides*, DJP and MSB obs. (see Table 1 for full data on the flights); 24.vii.2016, 38 ♂♂ and 30 ♀♀ flying mostly up to 0.5 m above the ground at 21.08–21.49 CEST, together with more spec. of *Od.armiger*, DJP, DVB, FSB and MSB obs. (see Table 1 for full data on the flights); 25.vii.2016, 14 ♂♂ and 1 ♀ FSLG at 21.06–21.41 CEST, 22 °C, light rain, no wind, FSB and MSB obs. (see Table 1 for full data on the flights); 26.vii.2016, 2 ♂♂ FSLG at 21.08–21.10 CEST, 24 °C, dry, gentle persistent wind, MSB obs.; 29.vii.2016, 6 ♂♂ and 2 ♀♀ excavated from their burrows on a loess-sandy path crossing a steppe meadow, together with 1 spec. of *Od.armiger* and 3 spec. of *Och.chrysomeloides*, DJP obs., and 10 ♂♂ and 3 ♀♀ FSLG at 21.02–21.30 CEST, 22 °C, wet vegetation, no wind, MSB obs. (see Table 1 for full data on the flights); 30.vii.2016, 1 ♂ excavated from its burrow on a loess-sandy path crossing a steppe meadow, DJP obs., and 16 ♂♂ and 21 ♀♀ FSLG at 20.53–21.56 CEST, together with ca. 20 spec. of *Od.armiger* and ca. 10 spec. of *Och.chrysomeloides*, DJP and MSB obs. (see Table 1 for full data on the flights); 7.viii.2016, 1 ♂ and 1 ♀ excavated from its burrow on a loess-sandy path crossing a steppe meadow, DJP obs., and 16 ♂♂ and 13 ♀♀ FSLG at 20.46–21.16 CEST, together with ca. 10 spec. of *Od.armiger*, 17 °C, no wind, DJP and IMO obs. (see Table 1 for full data on the flights); 8.viii.2016, 26 ♂♂ and 20 ♀♀ FSLG at 20.45–21.25 CEST, together with ca. 15 spec. of *Od.armiger* and 2 spec. of *Och.chrysomeloides*, 17–14 °C, no wind, DJP, ITV, FTV, IMO and JKO obs. (see Table 1 for full data on the flights); 13.viii.2016, 32 ♂♂ and 19 ♀♀ flying slowly up to 0.5 m above the ground at 20.40–21.01 CEST, together with ca. 30 spec. of *Od.armiger* and 7 spec. of *Och.chrysomeloides*, 19 °C, no wind, DJP, ITV and FTV obs. (see Table 1 for full data on the flights); 14.viii.2016, 2 ♂♂ and 3 ♀♀ FSLG after sunset, together with 4 spec. of *Od.armiger*, 20 °C, no wind, MSB and VKS obs.; 4.vii.2020, 1 ♂ excavated from its burrow on the edge of a path, DJP obs., 6 spec. FSLG after sunset, together with 1 spec. of *Od.armiger* and 8 spec. of *Och.chrysomeloides*DJP and FSP obs.; 6.viii.2020, 6 spec. FSLG at 21.05–21.15 CEST, FSP, IMO, PMB and VZO obs.

**Table 1.** Data on flights of adults of *B.unicornis* at the locality of PP Panský diel. Key: BF = beginning of flights, EF = end of flights, S = sunset, S-BF = time period from sunset to the beginning of flights, S-EF = time period from sunset to the end of flights, DF = duration of flights, T = air temperature during flights.

Slovakia, Bratislava – Podunajské Biskupice, Kopáč Island, PP Panský diel									
**date**	***n*** (♂/♀)	**BF**	**EF**	**S**	**S-BF**	**S-EF**	**DF**	**T**	**note**
7.vi.2016	11 (6/5)	21.25	21.45	20.47	38 min	58 min	20 min	14 °C	heavy dew, no wind
8.vi.2016	4 (4/-)	21.27	21.43	20.48	39 min	55 min	16 min	16 °C	dew, no wind
18.vi.2016	6 (3/3)	21.33	21.53	20.54	39 min	59 min	20 min	19 °C	dew, no wind
21.vi.2016	5 (3/2)	21.31	22.03	20.54	37 min	69 min	32 min	21 °C	no wind
22.vi.2016	6 (2/4)	21.21	21.57	20.55	26 min	62 min	36 min	22 °C	no wind
23.vi.2016	11 (6/5)	21.33	21.53	20.55	38 min	58 min	20 min	23 °C	no wind
24.vi.2016	24 (10/14)	21.36	22.08	20.55	41 min	73 min	32 min	25 °C	no wind
25.vi.2016	7 (2/5)	21.28	22.08	20.55	33 min	68 min	32 min	26 °C	no wind
26.vi.2016	25 (13/12)	21.31	22.09	20.55	36 min	74 min	38 min	20 °C	light breeze
27.vi.2016	10 (2/8)	21.31	21.57	20.55	36 min	62 min	26 min	20 °C	gentle breeze
28.vi.2016	9 (2/7)	21.28	21.48	20.55	33 min	53 min	20 min	19 °C	light breeze
29.vi.2016	14 (8/6)	21.29	22.03	20.55	34 min	68 min	34 min	24 °C	light dew, no wind
21.vii.2016	36 (25/11)	21.09	21.51	20.41	28 min	70 min	42 min	24 °C	no wind
22.vii.2016	51 (28/23)	21.08	21.51	20.40	28 min	71 min	43 min	23 °C	ground mist, no wind
23.vii.2016	41 (26/15)	21.09	21.53	20.39	30 min	74 min	44 min	24 °C	light ground fog, no wind
24.vii.2016	68 (38/30)	21.08	21.49	20.38	30 min	71 min	41 min	23 °C	dew, no wind
25.vii.2016	15 (14/1)	21.06	21.41	20.37	29 min	64 min	35 min	22 °C	light rain, no wind
29.vii.2016	13 (10/3)	21.02	21.30	20.32	30 min	58 min	28 min	22 °C	light dew, no wind
30.vii.2016	37 (16/21)	20.53	21.56	20.30	23 min	86 min	63 min	24 °C	light dew, no wind
7.viii.2016	29 (16/13)	20.46	21.16	20.19	27 min	57 min	30 min	17 °C	no wind
8.viii.2016	46 (26/20)	20.45	21.25	20.17	28 min	68 min	40 min	16 °C	no wind
13.viii.2016	51 (32/19)	20.40	21.01	20.09	31 min	52 min	21 min	19 °C	no wind
***n*** (♂/♀)	**519 (292/227)**	**average**			**32 min**	**65 min**	**32 min**	**21 °C**	

**7968**: Rusovce – Záhrady, 48°3'20.614"N, 17°9'18.014"E, 140 m a.s.l., 6.vi.2020, 1 ♂ flying ca. 10–20 cm above the ground at 21.20 CEST, small forest-steppe clea­ring in the forest, SRB obs. + photo (DJP det.); Bratislava – Podunajské Biskupice, Kopáč Island, PR Kopáčsky ostrov, ca. 48°5'41.97"N, 17°9'43.14"E, 132 m a.s.l., 13.vi.2006, 1 ♀, Malaise trap, MOB leg., coll. VKS; 30.v.2016, 2 ♂♂, and 3 ♀♀ FSLG after sunset, together with ca. 15 spec. of *Od.armiger*, EJB and RHB obs.; 7.vi.2016, 1 spec. FSLG after sunset, MSB obs.; 23.vi.2016, 2 ♂♂ FSLG at 21.32 and 21.38 CEST, MSB obs.; 1.vii.2016, 2 ♂♂ FSLG at 21.27 and 21.43 CEST, MSB obs.; 19.vii.2016, 4 spec. FSLG after sunset, EJB and JKB obs.; 20.vii.2016, 7 spec. FSLG after sunset, EJB and JKB obs.; ca. 48°5'39.5"N, 17°9'42.3"E, and 48°5'43.8"N, 17°9'30.4"E, 14.viii.2016, 7 spec. FSLG after sunset, DJP, ITV and VKB obs.; ca. 48°5'45.8"N, 17°9'41.9"E, 19.v.2018, 1 ♂ and 1 ♀ FSLG after sunset, MRV and VMP obs.; 9.vi.2018, 7 ♂♂ and 9 ♀♀ FSLG after sunset, together with ca. 20 spec. of *Od.armiger*, JHP, MRV and VMP obs.; 14.vii.2018, 1 ♂ and 4 ♀♀ FSLG after sunset, JRC and MRV obs.

**7869–7969**: “Štefánikovce” [= Rovinka near Dunajská Lužná], ca. 130 m a.s.l., May 1949, tens of spec. observed during the day sitting on the tops of the grass blades above the water on a flooded steppe meadow (after the flood), Josef Marvan obs., 2 spec. (♂ and ♀) leg., coll. IMP.

**Figure 6.**
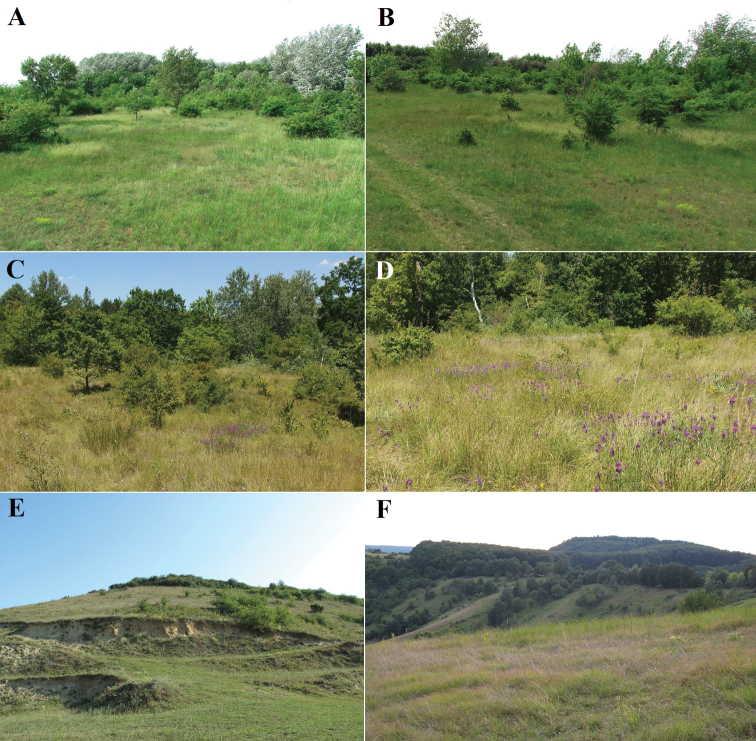
Slovak localities with *B.unicornis***A, B** Kalinkovo, Kalinkovská lesostep **C, D** Čunovo, PR Ostrovné lúčky (photographs by Milan Štrba) **E** Gemerský Jablonec (photograph by Ilja Trojan) **F** Hajnáčka (photograph by Ilja Trojan).

**7969**: Bratislava – Čunovo, PR Ostrovné lúčky (Fig. 6C, D), 48°2'28.02"N, 17°10'33.41"E, 138–139 m a.s.l., 21.vi.2016, 2 ♀♀ FSLG at 21.42 and 21.45 CEST, AHB obs.; ca. 48°2'24.5"N, 17°10'30.14"E and ca. 48°2'23.77"N, 17°10'34.47"E, 25.vii.2016, 6 ♂♂ and 4 ♀♀ flying slowly up to 0.5 m above the ground at 21.10–21.37 CEST, together with 7 spec. of *Od.armiger* and 3 spec. of *Och.chrysomeloides*, DJP obs. (see Table 2 for full data on the flights); 29.vii.2016, 1 ♂ and 1 ♀ flying quickly and 1 ♂ flying slowly up to 0.5 m above the ground at 21.11–21.25 CEST, together with 1 spec. of *Od.armiger* and 2 spec. of *Och.chrysomeloides*, DJP obs. (see Table 2 for full data on the flights); Kalinkovo env., Kalinkovská lesostep (Fig. 6A, B), 48°3'39.82"N, 17°12'37.94"E, 130 m a.s.l., 22.vi.2016, numerous ca. 2–3 weeks old burrows with push-ups weathered down, DJP and AHB obs.; 27.vii.2016, 1 ♂ and 2 ♀♀ flying up to 1 m above the ground at 21.04–21.15 CEST, together with 1 spec. of *Od.armiger*, DJP obs. (see Table 3 for full data on the flights).

**Table 2.** Data on flights of adults of *B.unicornis* at the locality of PR Ostrovné lúčky (for abbreviations see Table 1).

Slovakia, Bratislava – Čunovo, PR Ostrovné lúčky									
**date**	***n*** (♂/♀)	**BF**	**EF**	**S**	**S-BF**	**S-EF**	**DF**	**T**	**note**
25.vii.2016	10 (6/4)	21.10	21.37	20.37	33 min	60 min	27 min	24 °C	before rain, no wind
29.vii.2016	3 (2/1)	21.11	21.25	20.31	42 min	56 min	14 min	22 °C	no wind
***n***	**13 (8/5)**	**average**			**38 min**	**58 min**	**21 min**	**23 °C**	

**Table 3.** Data on flights of adults of *B.unicornis* at the locality of Kalinkovská lesostep (for abbreviations see Table 1).

Slovakia, Kalinkovo, Kalinkovská lesostep									
**date**	***n*** (♂/♀)	**BF**	**EF**	**S**	**S-BF**	**S-EF**	**DF**	**T**	**note**
27.vii.2016	3 (1/2)	21.04	21.15	20.34	30 min	41 min	11 min	22 °C	no wind

**7272**: Višňové, Čachtický hradný vrch hill, 22.v.1988, 1 ♂ dead on a forest-steppe slope, IPO leg., coll. DJP.

**7572**: Hlohovec env., Nová hora near Koplotovce, 48°28'24.98"N, 17°49'21.40"E, 260 m a.s.l., 12.iv.1988, 1 ♀, accidentally dug up while turning the soil in the garden, KPH leg., coll. VKS; Hlohovec env., Mlynárska hora near Koplotovce, 48°28'6.51"N, 17°49'29.04"E, 235 m a.s.l., 4.vi.2021, 1 ♀ at light at 21.10 CEST (= 27 minutes after sunset), TSH obs.

**7772**: Šoporňa, [ca. 122 m a.s.l.], July 1952, 1 ♂, Kotek leg., coll. MHKC.

**7373**: Modrovka, [ca. 170 m a.s.l.], 15.vii.1979, 1 ♀, Mrklovský leg., coll. Ladislav Bojčuk deposited in MHKC.

**7074**: Trenčín – Zlatovce [env.], June 1926, [Rudolf] Čepelák leg., 1 ♂ and 1 ♀ in coll. Josef Gottwald deposited in NHMB, 2 ♀♀ in coll. Paolo Luigioni deposited in MCZR, 1 ♂ in coll. Zdeněk Tesař deposited in SNMS, 1 ♂ in coll. MIZP; Trenčín – Zlatovce [env.], June 1926, 1 ♀, collector not specified, coll. TLMF; [Trenčín –] Zlatovce [env.], no other data, 2 ♀♀ in coll. Zdeněk Tesař deposited in SNMS; June 1931, L[adislav] Korbel leg., 1 ♂ in coll. JJP, 1 ♀ in coll. Ladislav Daněk deposited in MHKC; June 1935, 1 ♂ and 1 ♀ (ex coll. Johann Peter Wolf), “col. Kardasch” [= Gregor Kardasch leg.], coll. ETHZ.

**7174**: “Trencsen Ungarn” [= Hungary, Trenčín], undated, 1 ♂ and 1 ♀ (ex coll. Engelbert Pawlik) in coll. NMPC, 1 ♂ and 1 ♀ in coll. FMNH, 2 spec. in coll. ZSMG, 1 spec. in coll. MTDG, 2 ♀♀ (ex coll. P. Franck) in coll. MIZP, 1 ♀ (ex coll. † Richard Papperitz, Peutenhausen) in coll. SMNS; Trenčín, no other data, 3 spec. in coll. NHMW, 1 ♂ in coll. MNBG, 1 spec in coll. SZM; “Trencin Slow.” [= Slovakia, Trenčín], no other data, 1 ♀ in coll. Leopold Mader deposited in MNSA; “Trenčín, Tchécoslovaquie” [= Slovakia, Trenčín], undated, 1 ♂ and 1 ♀,“coll. J[oseph] Clermont”, coll. Jacques Baraud deposited in MNHN; “Slovakia Trenčín”, 2.vii.[year not specified], no other data, 1 ♀ in coll. MHKC; Trenčín, undated, [Rudolf] Čepelák [leg.], 6 ♂♂ and 5 ♀♀ in coll. Leopold Mader deposited in MNSA, 5 ♂♂ and 4 ♀♀ in coll. SNMS, 3 ♂♂ and 5 ♀♀ in coll. MHNG, 2 ♂♂ and 3 ♀♀ in coll. MNBG, 3 ♂♂ and 1 ♀ (ex coll. W. Liebmann, Arnstadt) in coll. SDEI, 2 ♂♂ and 2 ♀♀ in coll. Henri Coiffait deposited in MNHN, 3 ♂♂ and 1 ♀ (ex coll. Johann Peter Wolf) in coll. ETHZ, 3 ♂♂ and 1 ♀ in coll. MHKC, 2 ♂♂ (ex coll. Sten Stockmann) in coll. FMNH, 1 ♂ and 1 ♀ in coll. MIZP, 1 ♂ and 1 ♀ in coll. SMNS, 1 ♂ in coll. RBIN, 1 ♂ in coll. Ladislav Daněk deposited in MHKC, 1 ♀ in coll. Jacques Baraud deposited in MNHN, 1 ♂ in coll. Georg Frey deposited in NHMB, 1 ♂ and 1 ♀ in coll. Vladimír Zoufal deposited in MMBC, 1 ♀ in coll. Emil Jagemann deposited in MMBC, 2 spec. in coll. ZSMG, 1 ♂ (ex coll. Antonio Porta) in coll. MSNM, 1 spec. (head and pronotum missing) in coll. RMNH, 2 ♂♂ in coll. LEN, 1 ♂ in coll. DKC, 1 ♀ in coll. VKS; Trenčín, undated [most likely late 1920s/early 1930s], Z. Zeman leg., 1 ♀ in coll. SMNS, 1 ♂ in coll. VKS; Trenčín, undated, 1 ♂, V[ilém] Steidl [leg.], coll. MIZP; Trenčín, undated, [Ladislav] Krejcárek [leg.], 2 ♂♂ and 1 ♀ in coll. TMLS (see Kollár and Smetana 1994), 1 ♀ in coll. VKS; June 1925, 1 ♂, [Rudolf] Čepelák [leg.], coll. Jan Roubal deposited in SNMS; Trenčín, 16.vi.1928, 1 ♀, [Rudolf] Čepelák leg., coll. Jan Roubal deposited in SNMS; Trenčín, 18.vi.1929, [Ladislav] Korbel [leg.], 1 ♂ and 1 ♀ (ex coll. Dr J. B. Jörger, Masans bei Chur) in coll. NHMB, 1 ♂ in coll. FMNH; Trenčín, 1931, 3 ♂♂ and 3 ♀♀, [Rudolf] Čepelák [leg.], coll. Paolo Luigioni deposited in MCZR; Trenčín, May 1931, Dr A[lois] Richter leg., 1 ♀ in coll. NMPC, 1 ♀ in coll. MJMC; “Trencsin” [= Trenčín], undated, 1 spec., S. Kardasch [leg.], coll. SMNK; Trenčín, June 1935, 1 spec., G[regor] Kardasch [leg.], coll. SMNK; Trenčín, 1936, 2 spec., [Rudolf] Čepelák [leg.], coll. SMNK; Trenčín, June 1936, 1 ♂ and 1 ♀, [Rudolf] Čepelák [leg.], coll. VKS; Trenčín, July [19]36, 1 ♂ in coll. Jan Volák deposited in MHKC.

**7274**: [Považský Inovec Mts], Inovec [hill env.], 1 ♂, [Ladislav] Krejcárek [leg.], coll. Josef Gottwald deposited in NHMB.

**7674**: Nitra [env.], 1950, no other data, 1 ♂ in coll. MHKC.

**8177**: Štúrovo env., Belianské kopce hills, “Hegyfarok” [= Modrý vrch], 47°49'8.09"N, 18°39'32.4"E, ca. 150 m a.s.l., 20.viii.2005, 1 spec., at light after midnight, 1.ix.2005, 1 spec., at light after midnight, 14.vi.2006, 2 spec., at light, 15.vi.2006, 1 spec., at light, 15.vi.2007, 1 spec., at light, 29.vii.2008, 2 spec., at light, 30.vii.2008, 1 spec., VVO obs.; Modrý vrch, PR Vŕšok, 47°49'6"N, 18°39'33"E, ca. 150 m a.s.l., 22.v.2014, 1 ♂, at light, OSO obs.; 47°49'13.5"N, 18°39'21.5"E, ca. 195 m a.s.l., 4.vi.2015, 1 ♀ at UV light at 21.30–0.30 CEST and 1 ♀ at UV light (the same trap) at 1.15 CEST (5.vi.2015), OSO obs. (moreover ca. 50 spec. of *Och.integriceps* Semenov, 1891 in the light traps were observed); 6.vi.2015, 1 ♂ FSLG after sunset, anonymous observer from the Czech Republic obs. (moreover 1 spec. of *Och.integriceps* in the light trap was observed); 47°49'9.54"N, 18°39'26.4"E, ca. 170 m a.s.l., 27.v.2015, 1 spec. FSLG after sunset, and 1 spec. at light, two anonymous observers from the Czech Republic obs.

**8078**: Zalaba, 47°58'8.8"N, 18°42'29.2"E, ca. 150 m a.s.l., June 1975, 1 spec. crawling on the ground on a sandy slope sparsely covered with black locust trees (*Robiniapseudoacacia*) at ca. 19.00 CEST, JAH.

**Figure 7.**
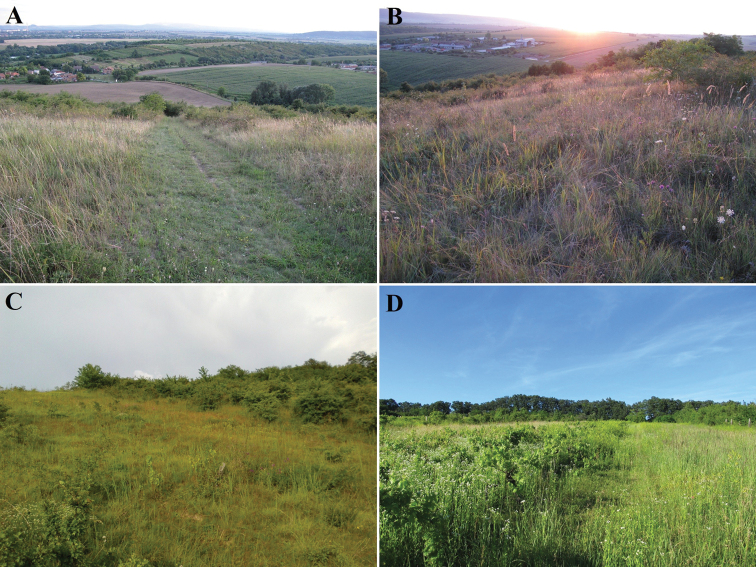
Biotopes of *B.unicornis* near Kamenica nad Hronom (Slovakia) **A–C** Čierna hora hill (**A, B** photographed by Ilja Trojan) **D** southwest facing slope northeast of Čierna Hora hill with old vineyards (photograph by Ondřej Sabol).

**Figure 8.**
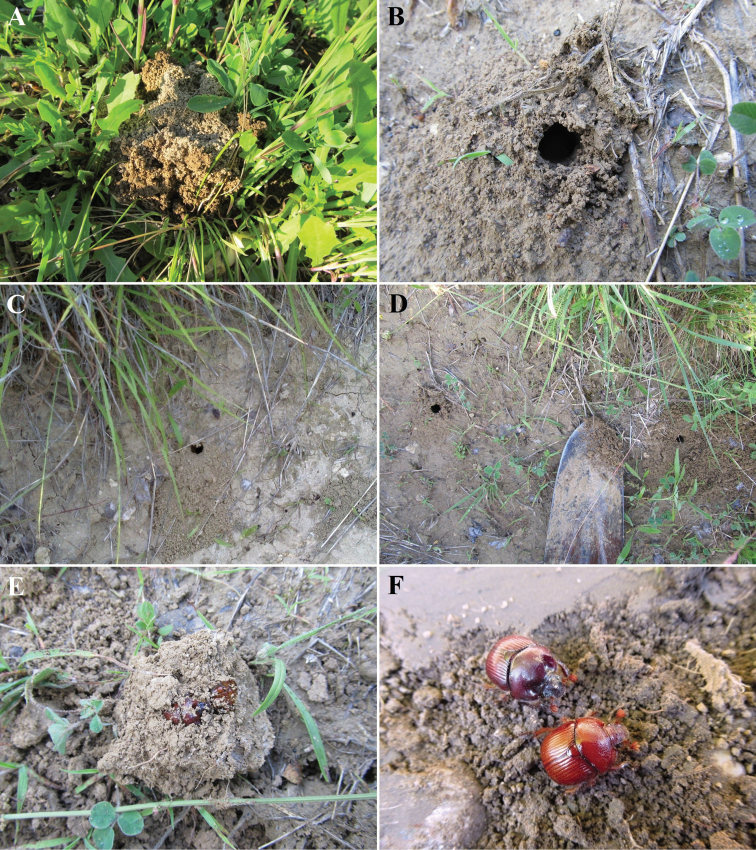
Excavation of *B.unicornis* at Čierna hora near Kamenica nad Hronom (Slovakia) **A–D** burrows dug by adults with push-ups (**A,B, D** photographed by Ondřej Sabol) **E, F** excavated pair (photographs by Ondřej Sabol).

**8178**: Bajtava, 16.vi.2006, 1 spec., at light, VVO obs.; Kamenica nad Hronom, Čierna hora hill (Fig. 7A–C), ca. 47°50'15"N, 18°43'34"E, ca. 180–190 m a.s.l., 27.vi.2009, 1 ♂ FSLG at 21.36 CEST, together with 1 ♂ a 2 ♀♀ of *Od.armiger*, OSO obs.; 5.vi.2010, 1 ♀ FSLG at 21.35 CEST, PJL obs.; 6.vi.2010, 3 ♂♂ and 1 ♀ FSLG at 21:30–21:45 CEST, and 1 ♂ at light, JHL, OSO and RSP obs. (see Table 4 for full data on the flights); 7.vi.2010, 16 spec. FSLG at 21:23–21.55 CEST, JHL, PJL, OSO and RSP obs. (see Table 4 for full data on the flights); 11.vi.2010, 4 ♂♂ flying relatively quickly ca. 1 m above the ground after sunset, light breeze to gentle breeze, DJP obs.; 12.vi.2010, 1 ♂ and 1 ♀ FSLG after sunset, light air to light breeze, DJP obs.; 26.vi.2010, 3 ♂♂ FSLG after sunset, LKK and PJL obs.; 27.vi.2010, 6 ♂♂ and 4 ♀♀ FSLG after sunset, LKK, PJL and OSO obs.; 30.vi.2010, 7 spec. FSLG after sunset, two anonymous observers from the Czech Republic obs.; 4.viii.2011, 17 spec. flying very slowly ca. 20–100 cm above the ground at 20.50–21.20 CEST, DJP and PJL obs. (see Table 4 for full data on the flights); 5.viii.2011, 10 spec. FSLG at 20.50–21.10 CEST, DJP and PJL obs. (see Table 4 for full data on the flights); 6.viii.2011, 9 spec. FSLG at 20.40–21.00 CEST, DJP and PJL obs. (see Table 4 for full data on the flights); 9.viii.2011, 15 spec. FSLG at 20.45–21.05 CEST, DJP and IMO obs. (see Table 4 for full data on the flights); 11.viii.2011, 14 spec. FSLG at 20.40–21.05 CEST, DJP, RKP and DHP obs. (see Table 4 for full data on the flights); 12.viii.2011, 9 spec. FSLG at 20.40–21.05 CEST, DJP, ITV and PJL obs. (see Table 4 for full data on the flights); 13.viii.2011, 16 spec. FSLG at 20.30–21.05 CEST, DJP, ITV and PJL obs. (see Table 4 for full data on the flights); 16.viii.2011, 1 ♂ and 1 ♀ FSLG at 20.35–21.05 CEST, DJP and RKP obs. (see Table 4 for full data on the flights); 7.vi.2013, 13 ♂♂ and 9 ♀♀ FSLG at 21.20–21.45 CEST, together with 11 spec. of *Och.integriceps*, DJP, IMO and ZKM obs. (see Table 4 for full data on the flights); 8.vi.2013, 10 ♂♂ and 5 ♀♀ FSLG at 21.17–21.42 CEST, together with 3 spec. of *Od.armiger* and 12 spec. of *Och.integriceps*, DJP, IMO and ZKM obs. (see Table 4 for full data on the flights); 9.vi.2013, 3 spec. FSLG after sunset, IJN and VVO obs.; 12.vi.2013, 6 spec. excavated from their burrows, OSO obs. (Fig. 8B, D, E, F), and 15 spec. FSLG at 21.20–21.35 CEST, together with more spec. of *Od.armiger*, DJP, JZJ and OSO obs. (see Table 4 for full data on the flights); 13.vi.2013, 10 spec. excavated from their burrows under a small piles of pushed-up soil, OSO obs. (Fig. 8A), 1 spec flying ca. 1 m above the ground just before the sunset, VLP obs., and 9 spec. FSLG at 21.30–21.50 CEST, OSO, PJL, VLP and VKS obs.; 14.vi.2013, 4 spec. FSLG after sunset, DJP, DKP, LKM and VKS obs.; 15.vi.2013, ca. 490 m NNE of the hilltop of Čierna hora hill, 3 spec. FSLG at 21.30 CEST, DKP, VKS and ZKM obs., SW hillside of Čierna hora hill, 7 spec. FSLG at 21.20–21.45 CEST, two anonymous observers from the Czech Republic obs. (see Table 4 for full data on the flights); 15.vi.2013, 10 ♂♂ and 10 ♀♀ FSLG after sunset, BBO and BJO obs.; 19.vi.2013, 1 ♀ FSLG at 21.46 CEST, RMU and OSO obs.; 15.vi.2014, 1 ♀ FSLG after sunset, BBO; 17.vi.2014, 1 ♀ FSLG after sunset, BBO; 3.ix.2014, 1 ♀ excavated from its burrow (from a depth of ca. 10 cm) on a path, ONV obs., 5 ♂♂ and 3 ♀♀ flying ca. 30–100 cm above the ground at 19.52–20.07 CEST, together with 1 ♂ of *Od.armiger*, DJP and ONV obs. (see Table 4 for full data on the flights); 4.ix.2014, 3 ♂♂ and 1 ♀ excavated from their burrows (from a depth of ca. 10–25 cm) on a path, 8 ♂♂ and 5 ♀♀ FSLG at 19.51–20.16 CEST, together with 1 ♂ of *Od.armiger*, DJP and ONV obs. (see Table 4 for full data on the flights); 5.ix.2014, 1 ♂ excavated from its burrow (from a depth of ca. 8 cm) on the edge of a path, 2 ♂♂ and 2 ♀♀ flying ca. 30–80 cm above the ground at 19.47–20.04 CEST, together with 3 ♀♀ of *Od.armiger*, DJP and VKS obs. (see Table 4 for full data on the flights); 6.ix.2014, 1 ♂ and 4 ♀♀ excavated from their burrows (from a depth of ca. 10–25 cm) on a path and on a loess forest-steppe slope, DJP and VKS obs.; 9.ix.2014, 3 ♂♂ and 1 ♀ excavated from their burrows (from a depth of ca. 10 cm) on a path, DJP, JCM and IMO obs., 1 ♂ flying relatively quickly ca. 1 m above the ground at 19.43 CEST and 1 ♂ flying very slowly ca. 10 cm above the ground at 19.47 CEST, DJP and IMO obs. (see Table 4 for full data on the flights); 17.ix.2014, 2 ♀♀ excavated from their burrows (from a depth of ca. 10 cm) on a path, IMO obs.; 31.v.2015, 1 ♂ and 1 ♀ flying slowly 10–30 cm above the ground at 21.40–21.45 CEST, FTR obs.; 3.vi.2015, 1 ♂ excavated from its burrow on a loess forest-steppe slope, 1 ♀ flying slowly near the ground after sunset and 1 ♀ attracted to the light trap, APO obs.; 4.vi.2015, 1 ♂ excavated from its burrow on a loess forest-steppe slope, APO obs.; 5.vi.2015, 2 ♂♂ flying relatively quickly ca. 1 m above the ground at 21.22–21.27 CEST, together with 1 ♀ of *Od.armiger* and 1 spec. of *Och.integriceps*, DJP obs. (see Table 4 for full data on the flights); 6.vi.2015, 2 ♂♂ excavated from a single burrow (from a depth of ca. 12 cm) and 1 ♀ excavated from another burrow (from a depth of ca. 25 cm) on a path (loess forest-steppe slope), 1 ♂ (length of body 9.5 mm!) and 1 ♀ flying relatively quickly ca. 120 cm above the ground at 21.24–21.29 CEST, together with 4 ♀♀ of *Od.armiger* and 1 spec. of *Och.integriceps*, and 1 ♂ flying slowly 10–20 cm above the ground at 21.47 CEST, DJP obs. (see Table 4 for full data on the flights); 28.v.2016, 6 spec. FSLG at 21.05–21.10 CEST, two anonymous obser­vers from the Czech Republic obs. (see Table 4 for full data on the flights); 11.vi.2016, 2 ♂♂ and 3 ♀♀ FSLG at 21.20–21.40 CEST, JKP and VDP obs.; 47°50'4.038"N, 18°43'53.947"E, ca. 165 m a.s.l., 2.viii.2014, 1 ♂ FSLG at 21.15 CEST, RCR obs.; 47°50'9.08"N, 18°43'39.93"E, ca. 185 m a.s.l., 20.vi.2018, 4 ♂♂ and 2 ♀♀ FSLG after sunset (3 spec.) and at light (3 spec.), OSO obs.; Kamenica nad Hronom env., ca. 530 m NNE of the hilltop of Čierna hora hill, 47°50'26"N, 18°43'50.7"E, 180 m a.s.l., 30.v.2011, 1 ♀ FSLG after sunset, small steppe hillside near an oak forest, FSP obs.; Kováčov, [110 m a.s.l.], 8.viii.1965, 1 ♂, K[arel] Poláček leg., coll. MHKC; Chľaba, 11.vi.1985, 1 ♂ and 1 ♀ J. Hladný leg., coll. JZJ.

**Table 4.** Data on flights of adults of *B.unicornis* at the locality of Čierná hora hill (for abbreviations see Table 1).

Slovakia, Kamenica nad Hronom env., Čierna hora hill									
**date**	***n*** (♂/♀)	**BF**	**EF**	**S**	**S-BF**	**S-EF**	**DF**	**T**	**note**
6.vi.2010	5 (4/1)	21.30	21.45	20.38	52 min	67 min	15 min	-	after the floods
7.vi.2010	16 (10/6)	21.23	21.55	20.39	44 min	76 min	32 min	-	-
4.viii.2011	17	20.50	21.20	20.18	32 min	62 min	30 min	18 °C	after ca. 10 days of persistent rainfall, ca. 2 hours after the rain has ceased, vegetation heavily soaked, no wind
5.viii.2011	10	20.50	21.10	20.16	34 min	54 min	20 min	20 °C	almost no wind
6.viii.2011	8	20.40	21.00	20.15	25 min	45 min	20 min	23 °C	almost no wind
9.viii.2011	15	20.45	21.05	20.10	35 min	55 min	20 min	17 °C	almost no wind
11.viii.2011	14	20.40	21.05	20.07	33 min	58 min	25 min	18 °C	almost no wind
12.viii.2011	9	20.40	21.05	20.05	35 min	60 min	25 min	18 °C	almost no wind
13.viii.2011	16	20.30	21.05	20.03	27 min	62 min	35 min	19 °C	almost no wind
16.viii.2011	2 (1/1)	20.35	21.05	19.58	37 min	67 min	30 min	22 °C	dry, almost no wind
7.vi.2013	22 (13/9)	21.20	21.45	20.39	41 min	66 min	25 min	18 °C	almost no wind
8.vi.2013	15 (10/5)	21.17	21.42	20.40	37 min	62 min	25 min	20 °C	light air to light breeze
12.vi.2013	15	21.20	21.35	20.43	37 min	52 min	15 min	18 °C	soil heavily saturated with water after rain, soaked vegetation
15.vi.2013	10	21.20	21.45	20.44	36 min	61 min	25 min	-	-
3.ix.2014	8 (5/3)	19.52	20.07	19.24	28 min	43 min	15 min	20 °C	light air to gentle breeze
4.ix.2014	13 (8/5)	19.51	20.16	19.22	29 min	54 min	25 min	22 °C	almost no wind
5.ix.2014	4 (2/2)	19.47	20.04	19.20	27 min	44 min	17 min	23 °C	almost no wind
9.ix.2014	2 (2/-)	19.43	19.47	19.12	31 min	35 min	4 min	22 °C	almost no wind
5.vi.2015	2 (2/-)	21.22	21.27	20.38	44 min	49 min	5 min	22 °C	almost no wind
6.vi.2015	3 (2/1)	21.24	21.47	20.39	46 min	68 min	27 min	23 °C	light air
28.v.2016	6	21.05	21.10	20.31	34 min	39 min	5 min	-	-
1.vi.2016	≈ 8	21.15	21.45	20.35	40 min	70 min	30 min	24 °C	no wind, very wet after rain
2.vi.2016	≈ 8	21.15	21.45	20.36	39 min	69 min	30 min	24 °C	no wind, very wet after rain
3.vi.2016	≈ 8	21.15	21.45	20.37	38 min	68 min	30 min	24 °C	no wind, very wet after rain
11.vi.2016	5	21.20	21.40	20.43	37 min	57 min	20 min	-	-
***n***	**241**	**average**			**36 min**	**58 min**	**22 min**	**21 °C**	

**8178–8278**: “Parkaň” [= Štúrovo], [ca. 110 m a.s.l.], 1934, no other data, 1 ♀ in coll. MHKC; 1940, no other data, 1 ♂ in coll. MHKC; Štúrovo, July 1967, 1 ♀, collector unknown, coll. ASH.

**8179**: Chľaba env., Močiar (the site near the confluence of the Danube and Ipeľ rivers), 47°49'14.53"N, 18°50'52.72"E, 110 m a.s.l., 12.vi.2014, 1 ♂ FSLG at 21.40 CEST, together with 2 ♀♀ of *Od.armiger*, OSO obs.

**7785**: Cerová vrchovina Mts, Hajnáčka – Buková env., ca. 48°13'51.39"N, 19°58'26.32"E, 1.vi.1978, 1 ♂ crawling on the ground in the afternoon in sunlight, IJN leg. [storage of the specimen unknown]; Cerová vrchovina Mts, Hajnáčka – Buková env., “circular pasture under vággon” [ca. 48°13'43.88"N, 19°58'15.53"E], 23.vi.1990, 1 ♀ flying at 21.28 CEST, JVP leg., ex original coll. JVP, currently in coll. NMPC; Hajnáčka – western edge of the village, 48°12'48.2"N, 19°56'52.1"E, ca. 275 m a.s.l., 7.vi.2010, 1 ♀ FSLG after sunset, together with more spec. of *Od.armiger* and *Och.chrysomeloides*, PVP obs.; 8.vi.2010, 1 spec. FSLG after sunset, together with more spec. of *Od.armiger* and *Och.chrysomeloides*, PVP obs.; Hajnáčka – Buková env., steppe hillside (former sheep pasture with low and sparse vegetation, near an oak forest), 48°13'37.24"N, 19°58'23.73"E, 340–390 m a.s.l., 27.v.2008, 5 ♀♀ FSLG at 21.10–21.35 CEST, 22 °C, no wind, together with 20 spec. of *Od.armiger* and 19 spec. of *Och.chrysomeloides*, DJP and FSP obs. (see Table 5 for full data on the flights); 28.v.2008, 1 ♀ FSLG at 21.20 CEST, 18 °C, light air – light breeze, together with 10 spec. of *Od.armiger* and 9 spec. of *Och.chrysomeloides*, FSP obs.; 29.v.2008, 1 newly hatched (light coloured) ♂ crawling on the T-shirt spread out on the ground near the edge of the forest, under an oak tree (*Quercuscerris*) at 19.55 CEST, 1 ♂ flying relatively quickly and zigzag ca. 1 m above the ground and 3 ♀♀ flying slowly ca. 0.5 m above the ground at 21.10–1.40 CEST, 21 °C, no wind to light air, together with 20 spec. of *Od.armiger* and 14 spec. of *Och.chrysomeloides*, DJP, KDO and PJL obs. (see Table 5 for full data on the flights); 30.v.2014, 1 ♂ FSLG at 21.10 CEST, 22 °C, almost no wind, together with 15 spec. of *Od.armiger* and 23 spec. of *Och.chrysomeloides*, DJP obs.; 28.vi.2009, 2 ♂♂ FSLG at 21.35 and 21.42 CEST, steppe hillside near an oak forest, together with 12 spec. of *Od.armiger* and 2 spec. of *Och.chrysomeloides*, OSO obs.; 29.vi.2009, 1 ♂ FSLG at 21.35 CEST, steppe hillside near an oak forest, together with 21 spec. of *Od.armiger* and 3 spec. of *Och.chrysomeloides*, OSO obs.; 4.vii.2009, 3 ♂♂ and 4 ♀♀ FSLG at 21.15–21.45 CEST, 22 °C, no wind, together with ca. 20 spec. of *Od.armiger* and ca. 15 spec. of *Och.chrysomeloides*, DJP and MBP obs. (see Table 5 for full data on the flights); 5.vii.2009, 1 ♂ and 2 ♀♀ FSLG at 21.15–21.30 CEST, 20 °C, no wind, together with ca. 10 spec. of *Od.armiger* and ca. 10 spec. of *Och.chrysomeloides*, DJP obs. (see Table 5 for full data on the flights); 6.vii.2009, 1 ♂ FSLG at 21:25 CEST, 21 °C, no wind, together with ca. 15 spec. of *Od.armiger* and ca. 10 spec. of *Och.chrysomeloides*, DJP obs.; 28.v.2010, 1 ♂ a 3 ♀♀ FSLG at 21.10–21.25 CEST, together with ca. 15 spec. of *Od.armiger* and ca. 25 spec. of *Och.chrysomeloides*, DJP obs. (see Table 5 for full data on the flights); 29.v.2010, 1 ♂ FSLG at 21.10 CEST, together with ca. 10 spec. of *Od.armiger*, DJP obs.; 7.vi.2010, 2 ♂♂ and 1 ♀ FSLG after sunset, FPT obs.; 48°13'30.26"N, 19°58'25.39"E, 305 m a.s.l., 20.vi.2020, 1 ♂ and 1 ♀ flying after sunset, JPH and TKH obs.; Hajnáčka, Tehliarske, 48°13'15.68"N, 19°57'45.57"E, ca. 270 m a.s.l., 8.viii.2014, 4 ♂♂ and 6 ♀♀ FSLG after sunset (ca. 21.07 CEST), RCR obs.; 8.vii.2015, 9 ♂♂ and 6 ♀♀ FSLG after sunset (ca. 21.11 CEST), RCR obs.; 29.vi.2017, 4 ♂♂ and 8 ♀♀ FSLG after sunset (ca. 21.07 CEST), RCR obs.; Hajnáčka, Lapos, 48°13'32.37"N, 19°57'50.58"E, ca. 350 m a.s.l., 24.vii.2020, 12 ♂♂ and 9 ♀♀ FSLG at 21.05–21.30 CEST, 15 °C, RCR obs.

**Table 5.** Data on flights of adults of *B.unicornis* at the locality of Hajnáčka – Buková (for abbreviations see Table 1).

Slovakia, Hajnáčka – Buková									
**date**	***n*** (♂/♀)	**BF**	**EF**	**S**	**S-BF**	**S-EF**	**DF**	**T**	**note**
27.v.2008	5 (-/5)	21.10	21.35	20.26	44 min	69 min	25 min	22 °C	first or second warm day after a colder period of persistent rainfall; no wind
29.v.2008	4 (1/3)	21.10	21.40	20.29	41 min	71 min	30 min	21 °C	newly hatched, light-coloured ♂ (not included in these statistics) crawling on a t-shirt spread on the ground at the edge of the forest under an oak tree (*Quercuscerris*) at 19.55 CEST; during flights, no wind to light air
4.vii.2009	7 (3/4)	21.15	21.45	20.43	32 min	62 min	30 min	22 °C	-
5.vii.2009	3 (1/2)	21.15	21.30	20.43	32 min	47 min	15 min	20 °C	-
28.v.2010	4 (1/3)	21.10	21.25	20.26	44 min	59 min	15 min	-	♂ flying fast and high (ca 1.5–1.8 m above the ground) around a pile of logs near the edge of the forest
***n*** (♂/♀)	**23 (6/17)**	**average**			**39 min**	**62 min**	**23 min**	**21 °C**	

**7785–7885**: Cerová vrchovina Mts, Gemerský Jablonec, 48°12'0.44"N, 19°59'24.31"E, 250–265 m a.s.l., steppe hillside with shrubbery of *Prunusspinosa* and *Rosacanina* on the hilltop, 4.vii.2009, 1 ♂ a 3 ♀♀ FSLG at 21.30–21.50 CEST, FPT and JPH obs. (see Table 6 for full data on the flights); 5.vii.2009, 3 ♂♂ a 1 ♀ FSLG at 21.30–21.50 CEST, FPT and JPH obesrv. (see Table 6 for full data on the flights); 28.v.2010, 1 ♂ and 3 ♀♀ FSLG at 21.00–21.15 CEST, ITV and MNB obs. (see Table 6 for full data on the flights); 29.v.2010, 1 ♂ and 1 ♀ FSLG after sunset, ITV and MNB obs.; 4.vi.2010, 1 ♂ FSLG at 21.30 CEST, together with 3 spec. of *Od.armiger*, ITV obs.; 8.vi.2010, 4 spec. FSLG after sunset, FPT and JPP obs.; 48°11'58.04"N, 19°59'23.71"E, 26.vi.2020, 1 spec. flying after sunset, JPH and TKH obs.; Gemerský Jablonec [env.], 5.vii.2013, 1 ♂ and 1 ♀, FPT leg., coll. GML.

**Table 6.** Data on flights of adults of *B.unicornis* at the locality of Gemerský Jablonec (for abbreviations see Table 1).

Slovakia, Gemerský Jablonec									
**date**	***n*** (♂/♀)	**BF**	**EF**	**S**	**S-BF**	**S-EF**	**DF**	**T**	**note**
4.vii.2009	4 (1/3)	21.30	21.50	20.43	47 min	67 min	20 min	-	-
5.vii.2009	4 (3/1)	21.30	21.50	20.43	47 min	67 min	20 min	-	-
28.v.2010	4 (1/3)	21.00	21.15	20.26	34 min	49 min	15 min	-	-
***n*** (♂/♀)	**12 (5/7)**	**average**			**32 min**	**65 min**	**32 min**		

**7786**: Cerová vrchovina Mts, Hostice – Katarínka env., 48°13'52.55"N, 20°5'0.82"E, 216 m a.s.l., small steppe hillside with rich low vegetation and shrubbery of *Prunusspinosa*, 6.vi.2010, 2 ♀♀, hovering on the spot ca. 20 cm above the ground at 21.15 and 21.30 CEST (25 spec. of *Od.armiger* and 4 spec. of *Och.chrysomeloides* were also observed at the site), DJP obs. (see Table 7 for full data on the flights); Cerová vrchovina Mts, Jestice env., 48°12'38.5"N, 20°03'07.3"E, 275 m a.s.l., 30.vi.2018, 1 ♂, 2.–3.vii.2018, 1 ♀, FPT and NKB leg., coll. GML; Jestice env., 48°12'54"N, 20°2'32"E, 6–7.vii.2019, 1 ♂ and 1 ♀ FSLG after sunset, JBB leg. (♀ in coll. IECA); Jestice – Kökényes, 48°12'45.84"N, 20°2'50.77"E, 250 m a.s.l., 23.vi.2020, 1 ♂ flying after sunset, ABC obs.; 6.vii.2020, 1 ♂ and 1 ♀ flying after sunset, ABC obs.; Jestice – Ivánkúta env., 48°12'30.9"N, 20°5'5.37"E, 254 m a.s.l., 7.vi.2015, 2 ♂♂ FSLG at 20.25 CEST, edge of an oak forest, RCR obs.

**Table 7.** Data on flights of adults of *B.unicornis* at the locality of Hostice – Katarínka (for abbreviations see Table 1).

Slovakia, Hostice – Katarínka									
**date**	***n*** (♂/♀)	**BF**	**EF**	**S**	**S-BF**	**S-EF**	**DF**	**T**	**note**
6.vi.2010	2 (-/2)	21.15	21.30	20.35	40 min	55 min	15 min	-	-

**7489**: Slovak Karst, “Rakaťa” [= Rakyta Cottage] env., 48°35'29.7"N, 20°34'01.45"E, ca. 540 m a.s.l., 5.vii.1988, 1 ♀ excavated from its burrow, DKP (for partial data on this record see Hillert et al. 2016).

**7390**: Slovak Karst, Hrhov, E of Okrúhle hill, 48°36'48.83"N, 20°47'22.98"E, ca. 395 m a.s.l., 4.vii.1988, 2 ♂♂ excavated from their burrows, DKP (for partial data on this record see Hillert et al. 2016); Slovak Karst, Hrhov, E of Okrúhle hill, 48°36'55.5"N, 20°47'27.3"E, ca. 430 m a.s.l., 28.v.2012, 1 ♂ drowned in a puddle on the path connecting two forest-steppe meadows, MHP obs.

**Comment**

**Figure 9.**
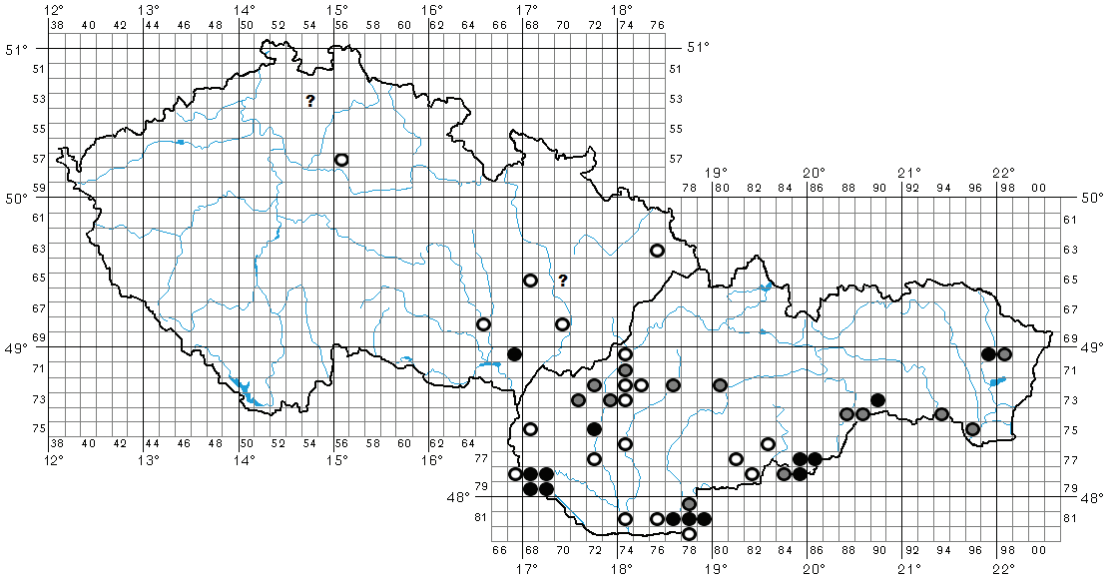
Distribution of *B.unicornis* in the Czech Republic and Slovakia (open circles refer to the records before 1960, open circles with a grey centre refer to the records between 1960–1999, and solid circles refer to the records after 1999; a question mark indicates a dubious record).

Slovakia is the country with the largest number of individuals found, as well as with the second largest number of known localities where the species has been recorded (52 sites). In addition to the already known localities, Endrődi (1957) mentioned Fehér Kárpátok [= The White Carpathians Mts], which most likely refers to two old records north of Trenčín (Nemšová – Ľuborča and Bolešov – Piechov) reported by Brancsik (1899, 1905), Laczó (1905), and Laco (1928). Most of the recent records are summarised by Juřena et al. (2008). New records from 26 Slovak localities are given in the present study. For the distribution of the species in Slovakia see Fig. 9.

### ﻿France

**Published data**

“Gallia”, no other data (Panzer 1802).

“Alsace”, no other data (Bedel 1911; Portevin 1931; Horion 1951; Peslier 2004; Callot 2018).

**Grand Est**, Bas-Rhin, Strasbourg, ca. 140 m a.s.l., “in coll. Dr Puton – Jules Bourgeois pers. comm.”, no other data (Scherdlin 1915; Sainte-Claire Deville 1936; Paulian 1941; [Bibr B303][Bibr B397]; Horion 1958; Paulian and Baraud 1982; Gangloff 1991; Brustel and Gouix 2012); Haut-Rhin, Colmar, ca. 195 m a.s.l., 25.vi.1967, 1 ♂, flew through the open window attracted by light, Schlatter leg. (Gangloff 1991; Brustel and Gouix 2012); Haut-Rhin, Mulhouse, no other data (Kampmann 1860; Knörzer 1912, Schaufuss 1916; Paulian 1941, 1959; Paulian and Baraud 1982), 1 ♂, undated, [Hans W.] Kesenheimer leg. (Scherdlin 1915; Gangloff 1991; Brustel and Gouix 2012), Haut-Rhin, Mulhouse – Dornach, ca. 250 m a.s.l., no other data, Klein leg. (Scherdlin 1920; Sainte-Claire Deville 1936; Paulian 1941, 1959; Horion 1958; Paulian and Baraud 1982), August (year not specified), 1 spec., collector unknown, coll. [Édouard] Klinzig (Gangloff 1991; Brustel and Gouix 2012); Haut-Rhin, Mulhouse – Tannenwald, ca. 300 m a.s.l., undated, several spec. excavated from their burrows, Oscar Koechlin leg. (Bourgeois 1904; Knörzer 1912; Huber 1916; Sainte-Claire Deville 1936; Paulian 1941, 1959; Horion 1958; Paulian and Baraud 1982); Haut-Rhin, Riedisheim, ca. 280 m a.s.l., July 1912, 2 spec., collector not specified (Sainte-Claire Deville 1936; Horion 1958), 15.vi.1949, 1 spec., [Édouard] Klinzig leg. (Gangloff 1991; Brustel and Gouix 2012); Haut-Rhin, Baldersheim, ca. 230 m a.s.l., 26.viii.1951, 4 spec., Burglin leg., coll. [Édouard] Klinzig (Gangloff 1991; Brustel and Gouix 2012).

? **Auvergne-Rhône-Alpes**, Savoie, Albertville, 19^th^ century, no other data, 1 ♂ and 1 ♀ in coll. Perroud deposited in MNHN (Brustel and Gouix 2012; according to Denis Keith pers. comm., 2020, this record is dubious – see also the comment below).

**Material examined**

? “S. Frankreich” [= south of France], 1 ♂ and 1 ♀, “Coll. C. Felsche, Kauf 20, 1918”, coll. MTDG [locality probably mistaken].

**Grand Est**, “Alsatia” [= Alsace], no other data, 1 ♂ in coll. Antoine Boucomont deposited in MNHN; Bas-Rhin, Strasbourg, [ca. 140 m a.s.l.], no other data, 1 ♂ in coll. MHNG (cf. Scherdlin 1915; Sainte-Claire Deville 1936; Paulian 1941, 1959; Tesař 1957; Horion 1958; Paulian and Baraud 1982); Haut-Rhin, Mulhouse – Dornach, [ca. 250 m a.s.l.], August [year not specified], 1 ♂, coll. MZSF (see Gangloff 1991); Haut-Rhin, Riedisheim, [ca. 280 m a.s.l.], 15.vi.1949, 1 ♂, [Édouard] Klinzig [leg.], coll. MZSF (cf. Gangloff 1991); Haut-Rhin, Baldersheim, ca. 230 m a.s.l., 26.vii.1951, 2 ♀♀, collector not specified [probably Burglin leg. – see Gangloff 1991], coll. MZSF.

? **Occitanie**, “Francia, Montpellier”, 1918, 1 ♀, Lavagne [leg.], coll. Paolo Luigioni deposited in MCZR [locality probably mistaken].

**Comment**

For France, *B.unicornis* was first recorded by Panzer (1802) without precise data. Up to now, it is reliably known only from Alsace, with the last record from Colmar in 1967 (Gangloff 1991). Brustel and Gouix (2012) reported two specimens from the 19^th^ century from the Savoy Prealps (Albertville), which, according to Denis Keith (pers. comm.), is dubious and probably based on mislabelled material. The site (a mountainous area) does not meet the known requirements of the species and its occurrence here seems to be highly improbable. The same applies to the Mont Cenis specimen from the Abeille de Perrin’s collection in the MNHN.

### ﻿Germany

**Published data**

**Baden-Württemberg**, Markgräflerland, Neuenburg am Rhein – Grißheim, “Grißheimer Trockenaue”, [ca. 47°52'18.3"N, 7°33'55.5"E, ca. 210 m a.s.l.], 2.vi.1967, 1 ♂, at light, Hans Messmer leg., photo + coll. Richard Disch (Brechtel et al. 1995; Krell 1998; Bense et al. 2000; Frank and Konzelmann 2002; Petersen et al. 2006).

**Bavaria (Bayern)**, “Bavaria”, no other data (Panzer 1793a, 1795); “Bayern”, no other data (Reitter 1909; Kuhnt 1912; Huber 1916; Horion 1951; Geiser 1984); “Bavaria”, no other data, 1 ♂ (ex original coll. Rudolf Veselý) in coll. NMPC (Hillert et al. 2016); Unterfranken, Aschaffenburg – Strietwald, ca. 130 m a.s.l., 1830, more spec., Dr Hoffmann leg. (Oechsner 1854; Kittel 1879; Ihssen 1935; Horion 1951, 1957, 1958) – note: Fröhlich (1897) and Knörzer (1912) consider record from Aschaffenburg to be doubtful; Oberbayern, Neuburg an der Donau – Bergheim, ca. 380 m a.s.l., 9.vii.1946, 1 spec. and 20.vii.1954, 1 spec., Rudolf Müller leg., coll. NMAG (Jungwirt 2005, 2012); ? Oberbayern, Ingolstadt, September 1892, 1 spec. K[arl] Daniel leg. (Horion 1957, 1958; Jungwirt 2005; see Material examined and new observations below).

**Material examined and new observations**

“Germ.” [= Germany], no other data, 1 ♂ and 1 ♀ in coll. ZINR, 1 ♀ in coll. Karel Mazura deposited in MMBC.

“Germania” [= Germany], no other data, 1 ♂ in coll. Georg Frey deposited in NHMB, 1 ♂ in coll. Ladislav Bojčuk deposited in MHKC, 1 ♂ in coll. NHMD.

“Germania mer.”, no other data, 1 spec in coll. ZSMG.

**Baden-Württemberg**, Bruchsal – Untergrombach, Michaelsberg and Habichtsbuckel Nature Reserve, ca. 49°5'32"N, 8°34'13"E, 200–220 m a.s.l., 3.vii.2021, 1 ♀, light trap, FTK and TBK obs., 4.vii.–5.viii.2021, 17 ♂♂ and 11 ♀♀ FSLG after sunset, together with more spec. of *Od.armiger* and *Och.chrysomeloides*, FTK and TBK obs. (5 spec. leg., coll. FTK, TBK and SMNK) – these records will be published with additional details at a later date (Florian Theves and Torsten Bittner pers. comm., 2021).

**Bavaria (Bayern)**, Upper Bavaria (Oberbayern), Ingolstadt, [ca. 370 m a.s.l.], 9.ix.[18]92, 1 spec. Dr K[arl] Daniel [leg.], “Fundortverwechslung” [= locality mistaken], coll. ZSMG (see Horion 1957, 1958; Jungwirt 2005); ? Oberbayern, “Holzapfelkr.” [= Holzapfelkreuth, former manor on the western outskirts of Munich], [ca. 550 m a.s.l.], 12.x.[19]12, H[ans] Kulzer [leg.], “Fundortverwechslung” [= locality mistaken], coll. ZSMG.

**Comment**

**Figure 10.**
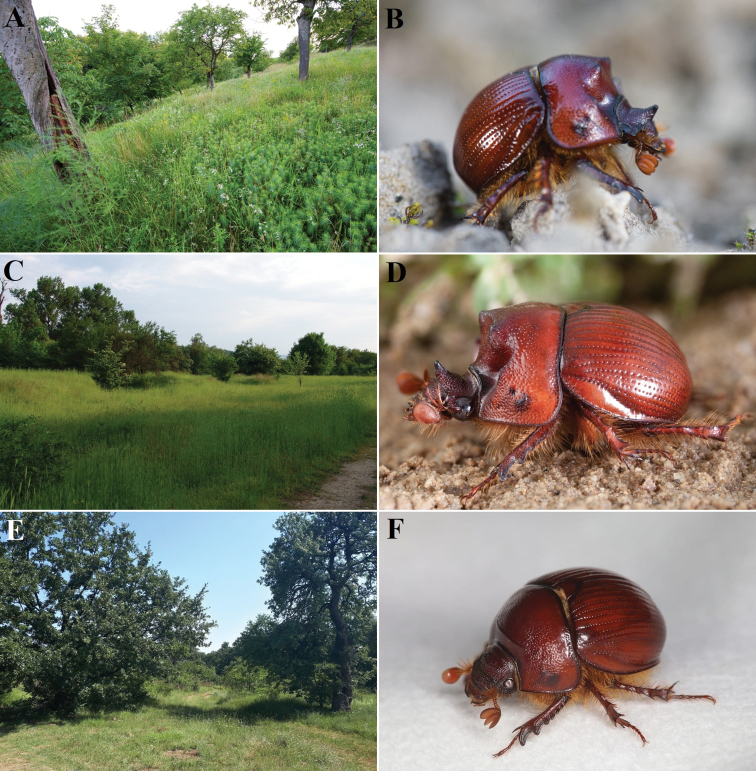
Findings of *B.unicornis***A, B** Germany, Bruchsal – Untergrombach, Michaelsberg and Habichtsbuckel Nature Reserve, 7.vii.2021 (photographs by Torsten Bittner) **C, D** Bulgaria, Dimovo env., 26.vi.2010 (photographs by Aleš Sedláček) **E, F** Bulgaria, Oreshak env., 6.vii.2020, (photographs by Maximilian Teodorescu).

In addition to old records from the late 18^th^ and the first half of the 19^th^ centuries from Bavaria, only one record from Baden from 1967 and two records from Bavaria in 1946 and 1954 were known from Germany. Daniel’s specimen from Ingolstadt and Kulzer’s specimen from Munich are questionable because they bear the labels added later of “Fundortverwechslung” (= locality mistaken). The new records presented from Baden represent the first known data on the species’ occurrence in Germany after 54 years (see also Fig. 10A, B).

### ﻿Switzerland

**Published data**

**Basel-Stadt (Kanton Basel-Stadt)**, Basel, undated, 1 spec., Ed. Bernoulli leg. (Heer 1841; Stierlin and Gautard 1867; Stierlin 1900; Huber 1916; Brustel and Gouix 2012).

? **Republic and Canton of Ticino (Repubblica e Cantone Ticino)**, no other data, Villa [leg.] (Heer 1841; Stierlin and Gautard 1867; Stierlin 1900); given that the canton of Ticino is mountainous, this record does not seem credible (see Habitat pre­ferences in this study).

**Material examined**

**Figure 11.**
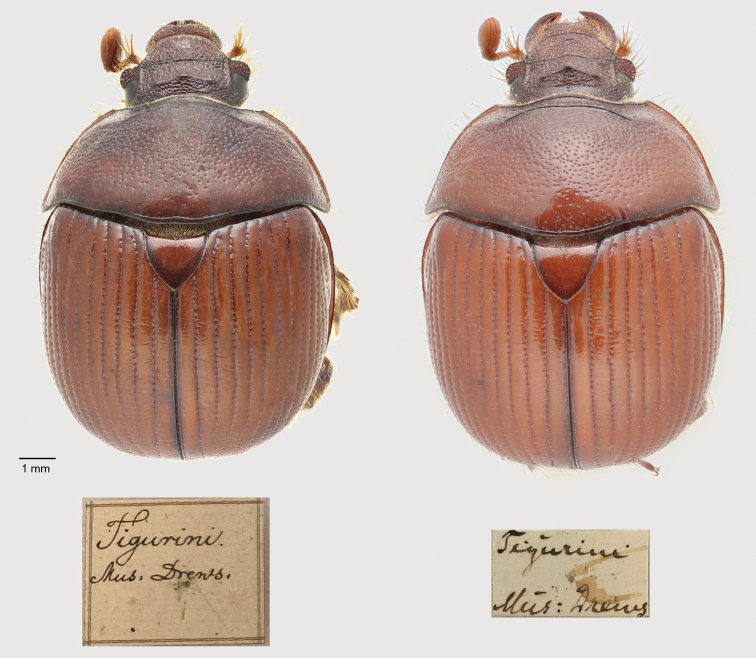
The only two specimens of *B.unicornis* so far known from Switzerland, deposited in NHMD (photographs by Caroline Amalie Høegh-Guldberg, edited by Peter Kurina).

**Canton of Zürich (Kanton Zürich)**, “Tigurini” [= Zürich], undated, 2 ♀♀, collector unknown, “Mus. Drews.” [= Musaeum Drewseni, = ex coll. Christian Drewsen (1799–1896)], coll. NHMD (Fig. 11).

**Comment**

The two old records from Basel and Canton of Ticino were later considered questionable for the absence of any subsequent sightings (Allenspach 1970). Both editions of the Catalogue of Palaearctic Coleoptera (Král et al. 2006; Nikolajev et al. 2016) list Switzerland for *B.unicornis* probably on the basis of these records. *Bolbelasmusunicornis* is no longer included in the very recent checklist of Scarabaeoidea of Switzerland (Cosandey et al. 2017). The two specimens from Zürich deposited in NHMD confirm the historical occurrence of the species in the country.

### ﻿Italy

**Published data**

“Italia”, no other data (Panzer 1802).

“Italia borealis”, no other data (Cristofori and Jan 1832).

**Piedmont (Piemonte)**, no other data (Marseul 1857; Jacquelin du Val 1863; Baudi di Selve 1889; Bertolini 1899a; Bedel 1911; Luigioni 1929; Porta 1932; Horion 1958; Arnone and Massa 2010; Brustel and Gouix 2012; Carpaneto et al. 2021), 2 spec., no other data (Costa 1864), 3 ♂♂ and 1 ♀, [19^th^ century], [Vittore] Ghiliani leg., coll. MSNG, 1 ♂, [19^th^ century], “ex coll. Demarchi”, [Flaminio] Baudi [di Selve] [leg.], coll. MSNG, 1 ♂ with no other data in coll. NMPC (Arnone and Massa 2010; Ballerio et al. 2014; Hillert et al. 2016; data from MSNG specified and supplemented by Roberto Poggi pers. comm., 2021), 1 ♂, [Flaminio] Baudi [di Selve] [leg.], and 1 ♂, L. Carrara [leg.], no other data, coll. MNFI (Arnone and Massa 2010; Hillert et al. 2016); Torino env., cattle pastures, date not specified, 2 spec. flying after sunset, together with *Od.armiger* and *Och.chrysomeloides*, Vittore Ghiliani leg. (Ghiliani 1847, 1887); Torino env., June 1845, 1 spec. on the bank of the Po River after a flood, Vittore Ghiliani leg. (Ghiliani 1847, 1887); Torino, 1 spec., no other data (Horion 1958; Barbero and Cavallo 1999); Torino, alluvial materials of the Po river, ca. 230 m a.s.l., 2 spec., no other data, coll. A. Gagliardi deposited in MFSN (Barbero and Cavallo 1999; information on the storage of these specimens supplemented by Enrico Barbero pers. comm., 2021); Provincia di Cuneo, Montelupo Albese, 24.v.1978, 1 ♂, collector not specified, coll. MCAS (Barbero and Cavallo 1999; Carpaneto et al. 2016; information on the storage of this specimen specified by Enrico Barbero pers. comm., 2021); Provincia di Alessandria, Lerma, 21^st^ century, no other data (Carpaneto et al. 2016; Glerean and Stefani 2019); Provincia di Novara, Bellinzago Novarese, Caserma Valentino Babini env., ca. 45°33'4"N, 8°39'59"E, ca. 185 m a.s.l., 1982–1989, number of spec. not specified, Roberto Pescarolo leg. et det. (Pescarolo 1990).

**Lombardy (Lombardia)**, no other data, (Bertolini 1872, 1899a; Luigioni 1929; Porta 1932; Horion 1958; Brustel and Gouix 2012; Carpaneto et al. 2021); “Milano” [= Milan], [ca. 120 m a.s.l.], [19^th^ century], 1 ♂ and 1 ♀, “ex coll. A[chille] Griffini”, no other data, coll. MSNG (Arnone and Massa 2010; Hillert et al. 2016; data specified by Roberto Poggi pers. comm., 2021).

**Trentino-Alto Adige/Südtirol**, “Tirol” [= probably Südtirol], [Stefano de] Bertolini leg. (Gredler 1863), Südtirol, no other data (Horion 1958); Provincia autonoma di Trento, Trento env., [ca. 190 m a.s.l.], September 1868, 1 [♂], plant materials alluviated by the flooded Adige River, together with *Od.armiger*, Stefano de Bertolini leg. (Bertolini 1871, 1874; note: in the first paper from 1871, Bertolini did not include *B.unicornis* in the list of identified species, but he added it in his later article from 1874) – this specimen, labelled “92", is still in the Bertolini’s collection deposited in MUSE; ? Provincia autonoma di Trento, Torcegno env., “in the mountains above Torcegno”, undated, 3 spec., together with *Od.armiger*, [Giovanni] Costesso leg., coll. Stefano de Bertolini (Bertolini 1891, 1899b) – this record seems improbable due to the very high altitude (ca. 1000–2300 m) of the area (cf. Habitat preferences in this study); Provincia autonoma di Bolzano, “Bozen Boden” [an urban area of Bolzano in the east of the city], [ca. 260 m a.s.l.], undated, 1 spec., coll. Vinzenz Maria Gredler (Gredler 1863; Peez and Kahlen 1977; Kahlen 2018; note: in the Gredler’s collection deposited in FGBI, the space for “*Bolb.quadridens*” in the box is empty – Daniel Lorenz pers. comm., 2021; in MSNB there are no specimens of *B.unicornis* – Petra Kranebitter pers. comm., 2021); Venezia Tridentina, no other data (Luigioni 1929; Porta 1932); Trentino, no other data (Arnone and Massa 2010; Brustel and Gouix 2012).

**Veneto**, no other data, (Bertolini 1872, 1899a; Luigioni 1929; Porta 1932; Horion 1958; Arnone and Massa 2010; Brustel and Gouix 2012).

**Friuli Venezia Giulia**, Provincia di Pordenone, Magredi del Cellina, Cordenons env., ca. 116 m a.s.l., 8.ix.2018, 1 ♂ and 1 ♀, dead on a path, 9.ix.2018, 1 ♂ and 1 ♀ in flight at 20.10–20.30 CEST (air temperature 21.5 °C, humidity 81%), 1 ♀ at actinic light at 21.15 CEST (air temperature 20 °C, humidity 96%), 10.ix.2018, 1 ♂ in flight at 20.15 CEST (air temperature 19 °C, humidity 78%), 12.x.2018, 1 ♀ in flight at 20.00 CEST, 15.v.2019, 1 ♂ and 1 ♀ in flight at 21.15 CEST (air temperature 14 °C, humidity 75%), 16.v.2019, 2 ♂♂ and 1 ♀ in flight at 21.00–21.15 CEST (air temperature 17 °C), 1 ♀ crawling on the ground at 21.20 CEST, 24.v.2019, 5 ♂♂ in flight at 21.20–21.35 CEST (air temperature 20 °C, humidity 70%), 26.v.2019, 1 ♂ in flight at 21.20–21.35 CEST (air temperature 20 °C, humidity 70%), 1.vi.2019, 1 ♂ and 2 ♀♀, in flight at 21.40 CEST (air temperature 20 °C, humidity 80%), 6.vi.2019, 2 ♂♂ and 1 ♀, in flight at 21.00–21.20 CEST (air temperature 22 °C, humidity 80%), 7.vi.2019, 2 ♂♂ in flight at 21.35 CEST (air temperature 22 °C, humidity 50%), Paolo Glerean and Gabriele Stefani obs. (Glerean and Stefani 2019; for flight statistics see Table 9); Provincia di Udine, Pasian di Prato, Biotopo prati del Lavia, ca. 90 m a.s.l., 15.–31.v.2005, 1 ♀, pitfall trap, Pietro Zandigiacomo leg. (Zandigiacomo 2005; Lapini et al. 2013).

**Tuscany (Toscana)**, no other data, coll. Dr L[ucas] von Heyden (Heyden 1884); 1 spec. with no other data (Horion 1958); 1 ♀ with no other data in coll. OHS (Hillert et al. 2016).

**Material examined and new observations**

“Ital.” [= Italy], no other data, 1 ♀ in coll. MNBG.

“Italia borealis”, 1 ♂, “ex coll. [Achille] Griffini”, no other data, coll. MSNG.

“Italien” [= Italy], “coll. [Gustav] Kraatz”, no other data, 2 ♂♂ and 5 ♀♀ in coll. SDEI.

“Italia”, undated, 1 ♂ and 1 ♀ (ex coll. Alexander Fry), coll. NHML.

“Italia, Sella [it is not clear whether it is a geographical name or the name of a person]”, no other data, 1 ♂ in coll. SDEI.

**Piedmont (Piemonte)**, “Pedem.”, [= Pedemontium, currently Piedmont], no other data, 1 ♂ in coll. RBIN; “Pedemt.” [= Piedmont], no other data, 3 ♂♂ and 1 ♀ in coll. Maurice Pic deposited in MNHN; “Pedemont.” [= Piedmont], no other data, 1 ♂ and 1 ♀ (ex coll. Christian Drewsen) in coll. NHMD, 1 ♂ (ex coll. Carl Gustaf Thomson) in coll. MZLU, 1 spec. in coll. NHMW; “Pedemont.” [= Piedmont], undated, L[éon Marc Herminie] Fairm[aire] [leg.], 1 ♂ and 1 ♀ (ex coll. Fredrik Wilhelm Mäklin) in coll. FMNH; “Alp. Pedemont.” [= Alpes Pedemontium], undated, 2 ♂♂ and 1 ♀, [Vittore] Ghiliani [leg.], coll. NHMD; “Piémont” [= Piedmont], undated, 1 ♂ in coll. Elzéar Abeille de Perrin deposited in MNHN, 1 ♀ in coll. Antoine Boucomont deposited in MNHN, 1 ♀ in coll. Jacques Baraud deposited in MNHN, 1 ♂ in coll. NMPC, 1 ♀, in coll. Alfonz Gspan deposited in PMSL, 1 ♂ (ex coll. Giacomo Doria, ex coll. Edward Bonney Nevinson) in coll. NHML; “Piemont” [= Piedmont], no other data, 1 ♂ and 1 ♀ in coll. MNBG, 1 ♂ and 1 ♀ in coll. RBIN; “Piemont” [= Piedmont], “coll. Rottenberg”, 1 ♀ in coll. SDEI; “Piemont” [= Piedmont], “coll. [Carl] Felsche”, 1 spec. in coll. MTDG; “Piemonte” [= Piedmont], “colezz. Alzona” [= coll. Alzona], 1 ♀ in coll. MSNM; Città metropolitana di Torino, Rivarossa, [ca. 285 m a.s.l.], no other data, 1 ♀ in coll. Leopold Mader deposited in MNSA; “Turin” [= Torino], no other data, 1 ♂ and 2 ♀♀ in coll. Sylvain Augustin de Marseul deposited in MNHN, 1 ♂ and 1 ♀ in coll. NHMD, 1 spec. in coll. ZSMG; Torino, 25.vii.[year not specified], no other data, 1 ♂ in coll. Georg Frey deposited in NHMB; Torino, “alluvioni Po” [= alluvial materials of the Po river], ca. 230 m a.s.l., 1871, 1 ♀, L. Fea leg., coll. MSNG; Torino, no other data, 1 ♂ in coll. FMNH, 1 spec. in coll. NHMW; Borgofranco d’Ivrea, [ca. 250 m a.s.l.], undated, 1 ♂, L. Demarchi leg., coll. MSNG; Provincia di Alessandria, Lerma, ca. 300 m a.s.l., May 1995, 1 ♂, in the morning accidentally dug up from the soil in the orchard; 8.iv.2014, 1 ♂, in the morning accidentally dug up from the soil in the orchard; 4.v.2014, 1 ♂ in the morning on the ground and 1 ♀ at UV light at 21.30 CEST, after several days of rain; 11.v.2014, 1 ♀, accidentally dug up from the soil in the garden at 16.00 CEST; 3.viii.2014, 1 ♀ flying around the light at 21.30 CEST; 16.v.2015, 1 ♂, at UV light at 21.30 CEST, rain in the morning and the day before, very wet, 17 °C; 17.v.2015, 1 ♂, at UV light at 21.15 CEST, wet, 17 °C; 20.vi.2015, 1 ♀, at UV light at 22.00 CEST, heavy rainfall in previous days, vegetation and soil heavily saturated with water, 17 °C; 21.vi.2015, 1 ♀, at UV light at 22.00, wet, 17 °C; 29.vi.2015, 1 ♀, at UV light at 21.50 CEST, 23 °C, LRL obs. (see Glerean et al. 2021).

**Lombardy (Lombardia)**, Provincia di Varese, Casorate Sempione, ca. 280 m a.s.l., October 1958, 1 ♂, at light, A. Bilardo leg., ex original coll. Giovanni Mariani, currently deposited in coll. RPM (for partial data on this record see Ballerio 2008 and Ballerio et al. 2014). Note: Zilioli and Pittino (2004) reported that in 2000 Riccardo Pittino unsuccessfully attempted to rediscover the species at this locality.

**Comment**

In the collection of Zdeněk Tesař deposited in SNMS, there is one specimen with the locality “Tirolis”, which may refer to the territory of South Tyrol (today Trentino-Alto Adige). Records from Sicily (Baraud 1977; Paulian and Baraud 1982; Carpaneto and Piattella 1995; Sparacio 1995; Barbero and Cavallo 1999; Martín-Piera and López-Colón 2000; Agoglitta et al. 2006; Trnka 2009; Arnone 2010; Alonso-Zarazaga et al. 2013; Schoolmeesters 2019; Nuß and Jäger 2020) refer to *Bolbelasmusvaulogeri* (Abeille de Perrin, 1898) (see Arnone and Massa 2010 and Hillert et al. 2016). Benasso’s record from Luint, Friuli-Venezia Giulia (Benasso 1971) is apparently based on a misidentified specimen of *Bolbocerosoma* sp. (bearing an erroneous locality label), which is evident both from the drawing of the specimen and from its description; in addition, this specimen was allegedly lost (Paolo Glerean pers. comm., 2020). This study presents new records from the third known locality with a recent occurrence of the species in Italy (Lerma).

### ﻿Poland

**Published data**

**Mazovian Voivodeship (Województwo mazowieckie)**, Warsaw – Saska Kępa, 80–85 m a.s.l., undated, 2 spec., Antoni Waga leg. (Hildt 1896; Tenenbaum 1923; Kubicka 1981; Szwałko 2004; Byk et al. 2012, 2016).

**Opole Voivodeship (Województwo opolskie)**, Opole County, Złotniki, ca. 155 m a.s.l., undated, 1 ♀, Ludwik Fryderyk Hildt leg. (Hildt 1896; Szwałko 2004; Byk et al. 2016).

**Świętokrzyskie Voivodeship (Województwo świętokrzyskie)**, Kielce County, Chęciny, 1 spec., no other data, (Tenenbaum 1923; Szwałko 2004; Bidas 2012; Byk et al. 2012, 2016; for details on this record see Material examined below); Ostrowiec County, Skarbka, 9.viii.1973, 1 ♂, dug up from the soil on a meadow, A. Liana, coll. MIZP (Stebnicka 1976; Szwałko 2004; Byk et al. 2016); Sandomierz County, Góry Pieprzowe Nature Reserve, ca. 150 m a.s.l., 28.vi.2001, 1 ♀, at light, KPL (Bunalski et al. 2013).

**Lublin Voivodeship (Województwo lubelskie)**, Lublin env., no other data, Baumgarten leg. (Hildt 1896; Szwałko 2004; Byk et al. 2016).

**Material examined**

**Lesser Poland Voivodeship (Województwo małopolskie)**, Wadowice County (Po­wiat wadowicki), “Wadowice, Hal.” [= Hałyczyna or Galicja (Galicia), Wadowice, ca. 250–300 m a.s.l.], 1 ♀, undated, Smolik [leg.], DJP det., coll. NMBE.

**Świętokrzyskie Voivodeship (Województwo świętokrzyskie)**, Kielce County, “Góry Stokrzyskie [env.], Gałęzice, [Mt.] Góra Ostrówka” [currently the Ostrówka quarry, ca. 50°50'11.94"N, 20°24'46.38"E, ca. 250 m a.s.l.], July 1921, 1 ♂, J. Czarnocki [leg.], “Polonia, [coll.] Sz[ymon] Tenenbaum”, coll. MIZP (for incomplete data on this record see Tenenbaum 1923; Szwałko 2004; Byk et al. 2012, 2016).

**Comment**

From Poland, only six records were known, which are summarised and specified by Byk et al. (2016). The last Polish record is from 2001 from the Góry Pieprzowe Nature Reserve (Bunalski et al. 2013). This study presents a previously unpublished historical record from Wadowice.

### ﻿Austria

**Published data**

**Upper Austria (Oberösterreich)**, Linz, Scharlinz, ca. 250 m a.s.l., 25.v.1936, 1 ♂, [Johann] Wirthumer leg., coll. BZLA (Mitter 2000; Schwarz 2008; sex specified by Martin Schwarz pers. comm., 2022); Linz, Weikerlsee, ca. 250 m a.s.l., 10.vii.1955, 1 ♂ and 3 ♀♀, after the flood, [Hermann] Haider leg., coll. BZLA (Mitter 2000; Schwarz 2008; sexes specified by Martin Schwarz pers. comm., 2022); Linz – Ebelsberg, bank of the Traun river, 10.vii.1954, 9 spec., F. Linzinger leg., 4 spec. in coll. HMS, 5 spec. [3 ♂♂, 2 ♀♀] in coll. BZLA, Linz env., undated, 2 spec. [1 ♂ and 1 ♀], [Emil] Munganast leg., coll. BZLA (Franz 1974; Mitter 2000; Schwarz 2008; sexes specified by Martin Schwarz pers. comm., 2022); Steyregg, ca. 250 m a.s.l., 1 ♀ with no other data, coll. BZLA (Franz 1974; Mitter 2000; Schwarz 2008; sex specified by Martin Schwarz pers. comm., 2022); bank of the Danube river between the villages of Steyregg and Pulgarn, driftwood, no other data (Dalla Torre 1879; Schwarz 2008); Saxen an der Donau, 21^st^ century, no other data (Paill and Mairhuber 2012; Gimpl et al. 2020).

**Lower Austria (Niederösterreich)**, no other data (Panzer 1793b; Sturm 1805); Mühling, ca. 260 m a.s.l., no other data, Arthur Schatzmayr leg. (Schatzmayr 1936; Benasso 1971); Schauboden env., Hochrieß, ca. 370 m a.s.l., end of July 1955, 1 ♂, F. X. Seidl leg., Rudolf Petrovitz det. et coll. (Ressl and Kust 2010); Melk, undated, 2 spec., [Josef] Breit leg. (Horion 1958; Franz 1974); Mödling env., Eichkogel hill., ca. 330 m a.s.l., no other data (Franz 1974; Schmölzer 1989); Weidling bei Wien, no other data (Duftschmid 1805); Wienerwald, Weidlingbach, undated, 2 spec., [Josef] Breit leg. (Horion 1958; Franz 1974); [Vienna env.,] “Donau-Auen”, undated, 3 spec., [Franz] Blühweiss leg. (Pittioni 1943; Horion 1958); Donau-Auen National Park, Orth an der Donau env., 48°7'59.87N, 16°42'20.56"E, 145 m a.s.l., 6.–8.vii.1997, 1 ♀, plant materials alluviated by flooded Danube river, PZW obs. + photo (Paill 2007; coordinates specified by PZW pers. comm., 2009); Groß-Enzersdorf – Mühlleiten env., 48°10'34"N, 16°33'6.6"E, 159 m a.s.l., 24.vi.2019, 1 ♂ flying up to 0.5 m above the ground at 21.40 CEST, meadow adjacent to the forest, 22 °C, gentle breeze, ADW obs. + photo (Dostal 2019; Dostal and Barries 2019; Dostal et al. 2021b); Leitha Mts, Mannersdorf am Leithagebirge env., July 1900, 1 ♀ and October 1912, 1 ♀, in a forest clearing, Molitor leg. (Horion 1958; Franz 1974); Oberweiden, Sandberge Oberweiden Nature Reserve, 48°17'15.4"N, 16°49'38.5"E, ca. 155 m a.s.l., 23.viii.2019, 1 ♀ perching motionless on a path at 19.00 CEST, 25 °C, DRW and SRL leg., det et coll. (Rabl et al. 2019); Marchegg, ca. 135 m a.s.l., no other data (Franz 1974).

**Vienna (Wien)**, no other data, coll. Dr Lucas von Heyden (Heyden 1884); no other data, (Dobiasch 1911); “Umg. Wien” [= Vienna env.], undated, 1 ♀, Ad[olf] Hoffmann [leg.], coll. ZFMK (Hillert et al. 2016); Vienna, Danube inundation area, 17.vii.1906, 3 spec., collector nor specified (Franz 1936, 1974); Vienna XXI [– Floridsdorf], August 1948, 12 spec., plant materials alluviated by flooded Danube river, [Harald] Schweiger leg. (Horion 1958); Vienna – Floridsdorf, ca. 155 m a.s.l., June 1949, 1 spec., at light in the garden, Harald Schweiger leg. (Schweiger 1951; Horion 1958; Franz 1974); Vienna env., Kahlenberg hill, no other data (Franz 1974); Vienna – Donaustadt, Fuchshäufel, 48°11'45.5"N, 16°28'57.9"E, 160 m a.s.l., 26.vi.2019, 1 ♂ flying up to 0.5 m above the ground at 21.55 CEST, 25 °C, no wind, WBW and ADW leg. + photo (Dostal 2019; Dostal et al. 2021a, b); Vienna – Donaustadt, Müllergraben, ca. 48°11'24.6"N, 16°30'42.4"E, 150 m a.s.l., 21.vi.2019, 1 spec., pitfall trap with vinegar, KFW leg. (Dostal 2019; Dostal et al. 2021a, b); Vienna – Donaustadt, Schusterau, 48°10'33.7"N, 16°32'54.7"E, 163 m a.s.l., 25.vi.2019, 1 ♀ flying up to 0.5 m above the ground at 21.50 CEST, 24 °C, no wind, WBW and ADW leg. + photo (Dostal 2019; Dostal et al. 2021a, b); Donau-Auen National Park, Untere Lobau, W of Kreuzgrund [= Lausgrund], ca. 48°9'34.52"N, 16°31'42.94"E, 152 m a.s.l., 15.vi.–9.vii.2006, 1 ♀, pitfall trap, Wolfgang Paill obs. (Paill 2007; Dostal et al. 2021b); Vienna – Donaustadt, Kreuzgrund, 48°9'36"N, 16°32'42"E, 160 m a.s.l., 12.vi.2019, one burrow, ADW and ADW obs.+ photo (Dostal 2019; Dostal et al. 2021a, b).

**Burgenland**, shore of Neusiedler See, plant materials amassed by flood water, seve­ral times (according to Sturm), with no further details (Petrovitz 1956); Winden am See, foot of the Zeilerberg mountain, ca. 200 m a.s.l., 3.vi.1981, 1 spec., at light, Gerhard Rößler leg. (Rößler 1989); Günser Gebirge, Rechnitz env., area of Geschriebenstein, no other data, Alfonz Freh leg. (Kaszab 1937; Horion 1958; Franz 1974); Jois env., steppe meadows north of the town, [ca. 220 m a.s.l.], 11.viii.2021, 1 ♂ flying up to 0.5 m above the ground and 1 ♀ in light trap, 21 °C, no wind, ADW and WBW obs. (Dostal and Barries 2021).

**Carinthia (Kärnten)**, Villach, Teufelsgraben, 1 spec. with no other data (Holdhaus and Prossen 1901; Horion 1958; Paill and Mairhuber 2006); Villach env., undated, 1 spec., Arthur Schatzmayr leg. (Prossen 1913; Schatzmayr 1936; Horion 1958; Benasso 1971; Paill and Mairhuber 2006).

**Styria (Steiermark)**, Grazer Bergland, Hörgas [near Gratwein-Straßengel], undated, 1 ♂ [10.6 mm], G[ustav] Wallaberger Sr. leg., coll. UMJG (Horion 1958; Franz 1974; Holzer 2019; sex specified by the author); Leutschach, Glanzer Klapotetzstraße 74 (Biohof Gunczy), 46°39'17.518"N, 15°31'18.03"E, ca. 370 m a.s.l., 8.ix.2018, 1 ♀, at light (flew through the open window), J. Gunczy obs., photo Gernot Kunz (Holzer 2019).

**Material examined**

“Styria” [= Duchy of Styria, a territory that included the modern Austrian state of Styria and the Slovenian region of Lower Styria], 1858, 1 ♂, [Eduard Albert] Bielz [leg.], coll. BNMS.

**Lower Austria (Niederösterreich)**, “Nied. Oesterr.” [= Niederösterreich], no other data, 1 ♂ in coll. NMBE; Melk, undated, 23 spec. in coll. NHMW, 1 ♂ and 1 ♀ (ex original coll. Josef Breit, Vienna) in coll. Georg Frey deposited in NHMB, 1 ♂ (ex original coll. Rudolf Petrovitz) in coll. MHNG (cf. Horion 1958 and Franz 1974); Wachau, no other data, 1 spec. in coll. NHMW; Wienerwald, Weidlingbach, undated, 2 ♂♂ (ex original coll. Josef Breit, Vienna) in coll. Georg Frey deposited in NHMB (cf. Horion 1958 and Franz 1974); “Umg. Wien” [= Vienna env.], Wienerwald, 1 spec. in coll. NHMW; “Blumau, Steinfeld” [= Blumau near Neurißhof], [ca. 250 m a.s.l.], undated, 1 ♂ (ex original coll. Rudolf Petrovitz) in coll. MHNG; [Vienna env.,] “Donau-Auen”, undated, 1 ♂ and 1 ♀, F[ranz] Blühweiss leg., 1 ♂ and 1 ♀, Fr. Reiss leg., ex original coll. Rudolf Petrovitz, currently in coll. MHNG; [Vienna env.,] Donauauen, no other data, 1 ♀ in coll. TLMF, 10 spec. in coll. NHMW; [Vienna env.,] Donauauen, undated, 1 ♀, F[ranz] Blühweiss [leg.], coll. MNBG; “Marchfeld, Oberweiden”, no other data, 1 ♀ in coll. MNBG; Oberweiden, Steppe [= steppe], 7.viii.1959, 1 ♀, J[osef] Gusenleitner leg., coll. BZLA.

**Vienna (Wien)**, “Wien” [= Vienna], no other data, 2 ♀♀ in coll. Vladimír Zoufal deposited in MMBC, 1 spec. in coll. MTDG, 1 ♀ in coll. BZLA; “Wien” [= Vienna], undated, 1 ♂, J[osef] Moser leg., coll. BZLA; “Vienne” [= Vienna], no other data, 1 ♂ and 1 ♀ in coll. Albert Sicard deposited in MNHN; “Wien Umg.,” [= Vienna env.], no other data, 1 ♂ in coll. Leopold Mader deposited in MNSA; “Wien, Umgebg.” [= Vienna env.], undated, 1 ♀, F. Schade [leg.], coll. Jaroslav Matoušek deposited in MMBC; “Wien Umgebgebung”, undated, 2 ♂♂, A[dolf] Hoffmann leg., coll. TLMF; “Umg. Wien” [= Vienna env.], undated, Ad[olf] Hoffmann [leg.], 1 ♀ (ex coll. P. Franck) in coll. MIZP, 1 ♂ in coll. SMNS, 1 ♀ in coll. Alfonz Gspan deposited in PMSL; “Hochwasser bei Wien” [= flood near Vienna], no other data, 1 ♀ (ex coll. Adolf Hoffmann) in coll. Jan Roubal deposited in SNMS; Vienna, Donau [= Danube river], Hochwasser [= flood], undated, 1 ♂ (ex original coll. Rudolf Petrovitz) in coll. MHNG; Vienna, “Donauüberschwemmung” [= flooded Danube river], September 1920, 1 spec., R. F. Lang [leg.], coll. NHMW; Vienna env., undated, 1 ♀, Carl Mandl [leg.], coll. Georg Frey deposited in NHMB; Vienna env., undated, 1 ♂, Matuschka [leg.], ex original coll. Josef Breit (Vienna), currently in coll. Georg Frey deposited in NHMB; Vienna, “Inundationsgebiet” [= inundation area of the Danube river], undated, 3 spec. (ex original coll. Herbert Franz) in coll. NHMW, 1 ♂ (ex original coll. Josef Breit, Vienna) in coll. Georg Frey deposited in NHMB; Vienna, Prater, no other data, 1 spec. in coll. NHMW.

**Comment**

In Austria, this species is known from six of the nine Austrian states. A recent attempt to rediscover the species at suitable sites along the Traun River in Upper Austria (Link et al. 2011) was unsuccessful, probably due to the use of inappropriate collecting methods and ignorance of the species’ bionomy. This study presents previously unpublished older data from three Austrian localities.

### ﻿Hungary

**Published data**

**Western Transdanubia (Nyugat-Dunántúl)**, Vas County, “Molna-Szecsőd” [= Molnaszecsőd], 10.vi. [turn of the 19^th^ and 20^th^ century], ca. 180 m a.s.l., 1 spec. inside the digestive system of *Cuculuscanorus*, Ernő Csiki obs. (Csiki 1904); Vas County, Kőszegi-hegység, no other data (Endrődi 1957); Zala County, Nova, ca. 190 m a.s.l., no other data (Endrődi 1957).

**Central Transdanubia (Közép-Dunántúl)**, Komárom-Esztergom County, “Szőny” [a part of the current Komárom city], 6.viii.1901, 2 spec. inside the digestive system of *Upupaepops*, Ernő Csiki obs. (Csiki 1905); Komárom-Esztergom County, “Ószőny” [= Szőny, the part of the current Komárom city], ca. 105 m a.s.l., no other data (Endrődi 1957); Komárom-Esztergom County, Esztergom, 1 ♂, Sebő Endrődi leg., coll. HNHM (Endrődi 1957; Nádai 2006; sex specified by VKS pers. comm., 2020); Komárom-Esztergom County, Csolnok, no other data (Endrődi 1957), 28.v.1898, 1 ♂, Zahradka leg., coll. HNHM (Nádai 2006; sex specified by VKS pers. comm., 2020); Oroszlány env., Majkpuszta, Majki-hegy, 14.vi.1997, 1 ♀, at light. CKZ (Kutasi 2002; Nádai 2006; Eichardt and Kutasi 2011); Fejér County, Velence – Kisvelence, ca. 115 m a.s.l., July 1940, 1 ♀, Rudolf Lenczy leg., coll. HNHM (Nádai 2006; sex specified by VKS pers. comm., 2020); Fejér County, Adony, no other data (Endrődi 1957), 1 ♂, undated, Viktor Stiller leg., coll. HNHM (Nádai 2006; sex specified by VKS pers. comm., 2020).

**Southern Transdanubia (Dél-Dunántúl)**, Somogy County, Fonyód, ca. 140 m a.s.l., undated, 1 ♂, Viktor Stiller leg., coll. HNHM (Endrődi 1957; Nádai 2006; sex specified by VKS pers. comm., 2020); Somogy County, Ordacsehi, Csehi-berek, 21.vii.2004, György Rozner leg. (Nádai 2006); Somogy County, Kaposvár, no other data (Endrődi 1957), 8.vii.1931, 1 ♂, Miklós Nattán leg., coll. HNHM (Hillert et al. 2016), 22.v.1951, 1 ♂, 3.vi.1951, 1 ♂, 4.vii.1951, 1 ♂, 31.vii.1958, 1 ♂, 19.v.1960, 1 ♀, Miklós Nattán leg., coll. HNHM (Nádai 2006; sex specified by VKS pers. comm., 2020); Somogy County, Balatonföldvár, no other data (Endrődi 1957); Somogy County, Nagyberény, 1937, 1 ♂, Ferenc Lichtneckert leg., coll. HNHM (Nádai 2006; sex specified by VKS pers. comm., 2020); Somogy County, Balatonvilágos – Balatonaliga, 1.viii.1980, collector unknown, 1 spec. in coll. HNHM (Nádai 2006; data specified by OMB pers. comm., 2020); Somogy County, Szenna, 9.vi.1998, György Rozner leg. (Rozner 2001; Nádai 2006); Tolna County, Gyulaj, 1 ♂, 1952, Jenő Győrffy leg., coll. HNHM (Nádai 2006; sex specified by VKS pers. comm., 2020); Tolna County, Hőgyész, 46°30'38"N, 18°25'55"E, 24.vii.1994, 1 spec. at light, collector not specified (Nádai 2006; coordinates specified by SBP, pers comm. 2021); Tolna County, Bátaapáti env., Nagy-mórágyi-völgy [valley], Quercetum, 15.vii.2004, 1 ♀, OMB leg., coll. HNHM (Nádai 2006; Hillert et al. 2016); Tolna County, Bonyhád, ca. 140 m a.s.l., 8.vii.1938, 1 spec., Nándor Vámos leg., coll. ZUDH (Enyedi 2006); Baranya County, Szigetvár, ca. 120 m a.s.l., no other data (Endrődi 1957), 1.vi.1909, remains of 1 spec., Ottó Mihók leg., coll. HNHM (Nádai 2006; data specified by VKS pers. comm., 2020); Baranya County, Sellye, finding in truffle (*Tuber* sp.), no other data (Merkl 2014); Baranya County, Pécs, no other data, (Viertl 1894; Kuthy 1898; Endrődi 1957), 1 ♂, undated, Ferenc Ehmann leg., coll. HNHM (Nádai 2006; sex specified by VKS pers. comm., 2020); Baranya County, “Szabolcs” [= Pécs – Szabolcs or Mecsekszabolcs] env., “Szarvasnóta”, ca. 46°8'8"N, 18°15'46"E, beginning of June 1880, 1 ♂ and 1 ♀, the female was digging a hole into the ground at the edge of a fo­rest footpath like *Copris*, and it seemed that the male was helping her with this work, Dr Ernő Kaufmann leg. (Kaufmann 1897, 1914a, b); Baranya County, Abaliget, 1978, no other data (Nádai 2006); Baranya County, Villányi-hegység Mts, Csukma-hegy hill, 5.v.1972, 1 spec. at light (mercury-vapor lamp), Ákos Uherkovich leg. (Horvatovich 1980; Sár and Horvatovich 2000; Nádai 2006).

**Central Hungary (Közép-Magyarország)**, Veszprém County, Pápa env., no other data (Wachsmann 1907; Endrődi 1957), 1893, 1 ♀, Ferenc Wachsmann leg., coll. HNHM (Nádai 2006; sex specified by VKS pers. comm., 2020), June 1895, 1 ♀, Fe­renc Wachsmann leg., coll. HNHM (Rozner 1984; Nádai 2006; sex specified by VKS pers. comm., 2020); Veszprém County, Balatonalmádi, 5.ix.1940, 1 ♂, Ernő Csiki leg., coll. HNHM (Rozner 1984; Nádai 2006; sex specified by VKS pers. comm., 2020); Veszprém County, Vászoly env., Öreg-hegy, 250–290 m a.s.l., 3.vii.1999, 1 spec., IRB leg. (Nádai 2006; data specified by OMB pers. comm., 2020); Veszprém County, Paloznak 17.viii.1961, Frigyes Novák leg., coll. HNHM (Rozner 1984; Nádai 2006); Veszprém County, Berhida, undated, 1 ♀, Rudolf Lenczy leg., coll. HNHM (Rozner 1984; Nádai 2006; sex specified by VKS pers. comm., 2020); Pest County, Buda hills (Budai-hegység), no other data (Frivaldszky I. 1865; Frivaldszky J. 1879a, b; Endrődi 1957); Pest County, Buda Hills, Hármashatárhegy [env.], [47°32'50.21"N, 19°0'18.77"E, ca. 390 m a.s.l.], 31.v.2004, 1 ♂, caught after sunset with a net attached to the roof of a moving car, OMB leg., coll. HNHM (Hillert et al. 2016; data specified by OMB pers. comm., 2020); Pest County, “Kis-Szent-Miklós” or “Őrszentmiklós” [= Őrbottyán – Őrszentmiklós], 1876, dry oak forest on the hill, 1 spec. on the ground in the grass in the evening (localised thanks to audible stridulation), Karoly Sajó leg. (Sajó 1881, 1897, 1910a, b), 1880s, more spec., sons of Karoly Sajó leg. (Sajó 1897, 1910b); Pest County, Sződliget, 16.vi.2005, 1 spec., Tamás Hácz leg. (Nádai 2006; data specified by OMB pers. comm., 2020); Pest County, Pilis hegység, no other data (Endrődi 1957); Pest County, Pilis hegység, Szentendre env., undated, 1 ♀, Hugó Die­ner leg., coll. HNHM (Nádai 2006; sex specified by VKS pers. comm., 2020); Pest County, Szigetszentmiklós, 6.vi.1954, 1 ♀, Miklós Nattán leg., coll. HNHM (Nádai 2006; sex specified by VKS pers. comm., 2020); Pest County, Dabas, no other data (Frivaldszky 1865; Kuthy 1898; Endrődi 1957); Pest County, Dabas – Gyón env., [47°9'6.08"N, 19°18'6.84"E, ca. 100 m a.s.l.], 20.v.2012, 1 spec., at light, SIB leg. (Merkl and Szénási 2018; coordinates specified by OMB pers. comm., 2020); Pest County, Táborfalva env., shooting and training area, [47°5'52"N, 19°23'26"E, 118 m a.s.l.], 11.vii.2012, 1 spec., at light, SIB obs. (Merkl and Szénási 2018; coordinates specified by SBP pers. comm., 2021); Pest County, Gödöllő env., no other data (Pétsch and Szénási 2019); Pest County, Gödöllő env., Valkó, 22.vii.1992, 1 spec., clearing in an oak forest, László Köteles leg. (Köteles and Bakonyi 1996; Nádai 2006); Pest County, Gödöllő – Máriabesnyő, no other data (Endrődi 1957), 31.v.1912, 1 ♂, István Gurányi leg., coll. HNHM (Nádai 2006; sex specified by VKS pers. comm., 2020); Pest County, Gödöllő, 55 Erdőszél Street, [47°36'11.3"N, 19°23'23.6"E, 250 m a.s.l.], 2005, no other data, VSI leg., (Nádai 2006); Pest County, Gödöllő env., Faház-tető hill, no other data, VSI leg., (Nádai 2006); Pest County, Pécel, no other data (Kuthy 1898; Endrődi 1957; Pétsch and Szénási 2019), 1 ♀, undated, István Peregi leg., coll. HNHM (Nádai 2006; sex specified by VKS pers. comm., 2020); 47°29'11.48"N, 19°23'19.52"E, 3.vi.1972, 1 spec., IRB leg. (Nádai 2006; data specified by OMB pers. comm., 2020); Pest County, Isaszeg, no other data (Endrődi 1957; Pétsch and Szénási 2019); 29.v.1909, 1 ♂, 2.vi.1909, 1 ♂, Ottó Mihók leg., coll. HNHM (Nádai 2006; corrections and sex specified by VKS pers. comm., 2020); 1908, 1 ♀, 1909, 1 ♀, June 1917, 1 ♂, Hugó Diener leg., coll. HNHM (Nádai 2006; corrected and sex specified by VKS pers. comm., 2020); June 1929, 1 spec., Hugó Diener leg., coll. HNHM (Nádai 2006); 2008, 1 ♀, collector not specified, coll. HNHM (Hillert et al. 2016); June 2013, more spec. FSLG after sunset, TNB obs. + photo (Németh 2013); Pest County, Isaszeg, 29 Erdő Street, [47°31'23.412"N, 19°23'33.87"E, 210 m a.s.l.], 2005, VSI leg. (Nádai 2006); Pest County, Kistarcsa env., no other data (Pétsch and Szénási 2019); Pest County, Kerepes env., no other data (Pétsch and Szénási 2019); Pest County, Domony env., no other data (Pétsch and Szénási 2019); Pest County, Csévharaszt, [47°18'26"N, 19°26'26"E, 127 m a.s.l.], 14–15.viii.2001, 3 ♂♂ and 1 ♀, pitfall traps with ethylene glycol and at light, GSB leg., coll. HNHM (Nádai 2006; Szél and Kutasi 2011; Hillert et al. 2016; data specified by VKS pers. comm., 2020, and SBP pers. comm., 2021), 17.vi.2002, 1 spec., at light, SIB obs., 19.vi.2004, 1 spec., at light, SIB obs., 29.v.2005, 1 spec., at light, SIB obs. (Nádai 2006; Szél and Kutasi 2011; data specified by OMB pers. comm., 2020); Pest County, Biatorbágy, 27.vi.1999, 1 ♂, at light, AGB leg., coll. HNHM (Nádai 2006, Hillert et al. 2016; data specified by VKS pers. comm., 2020); Pest County, Nagykovácsi env., Julianna-major, 10.vi.1985, 1 spec., at light, 18.vii.1985, 1 spec., at light, Dezső Szalóki leg. (Nádai 2006); Pest County, Budakeszi, 28.v.1991, on *Glomusmacrocarpum*, no other data (Bratek et al. 1992); Pest County, Budakeszi env., Hosszú-dűlő, 200 m a.s.l., 5.vi.1991, 2 ♀♀, on *Glomusmacrocarpum*, *Cynodonto-Festucetum*, LAB leg. coll. HNHM (Nádai 2006; data specified by VKS pers. comm., 2020), 8.vi.1991, 2 ♀♀, on *Glomusmacrocarpum*, *Cynodonto-Festucetum*, LAB leg., coll. HNHM (Nádai 2006; Hillert et al. 2016); Pest County, Budakeszi env., airport, 5.vi.1991, 1 ♀, LNB (Nádai 2006); Pest County, “Nógrádverőce” [= Verőce], Borbély-hegy hill, 1 ♂, summer 1916, municipal forest, Sebő Endrődi leg., coll. HNHM (Endrődi 1957, 1979; Nádai 2006; sex specified by VKS pers. comm., 2020); Buda [currently western part of Budapest], “Graberl” [a historical excursion destination in the Buda surroundings], 13.v.1798 (!), 1 spec., Tóbiás Koy leg. (Horváth 1884; this record is a quotation from the unpublished diary of János Boehm, the pioneer of Hungarian entomology); Budapest, no other data (Kuthy 1898); Budapest, 22.v.1930, no other data, Hugó Diener leg. (Nádai 2006); Budapest – Hűserbiavösvölgy, 9.vi.1939, 1 ♀, József Stahulják leg., coll. HNHM (Nádai 2006; sex specified by VKS pers. comm., 2020); Budapest env., Kamaraerdő, 25.iv.1920, 1 ♂ and 30.v.1922, 1 ♀, Hugó Diener leg., coll. HNHM (Nádai 2006; data specified by VKS pers. comm., 2020); [Budapest –] Rákos, no other data (Frivaldszky 1879a, b); Budapest – Budafok, no other data (Endrődi 1957), 1 ♂ with no other data, coll. HNHM (Nádai 2006; sex specified by VKS pers. comm., 2020); Budapest – Cinkota, no other data (Endrődi 1957), July 1907, 1 ♀, Kálmán Szombathy leg., coll. HNHM (Nádai 2006; sex specified by VKS pers. comm., 2020); Budapest – Mártonhegy, 17.iii.1949, 1 ♂, József Szőcs leg., coll. HNHM (Nádai 2006; Hillert et al. 2016); Budapest – Békásmegyer, 27.vi.1954, 1 spec., 29.vi.1954, 1 spec. and 1.vii.1954, 1 spec., Attila Podlussány leg., coll. MMGH (Nádai 2006; Enyedi and Ádám 2009); Budapest – Normafa, 9.vi.1967, 1 spec., Kálmán Gaskó leg. (Nádai 2006; data specified by OMB pers. comm., 2020); Budapest – Nagytétény, undated, 1 ♂, Sebő Endrődi leg., coll. HNHM (Endrődi 1957; Nádai 2006); Budapest – Ördög-orom, 2.vi.1959, 1 spec., Kálmán Gaskó leg. (Nádai 2006; data specified by OMB pers. comm., 2020); Budapest – Rákosszentmihály, 15.viii.1930, 1 ♂, at light, Jenő Győrffy leg., coll. HNHM (Nádai 2006; data specified by VKS pers. comm., 2020); Budapest – Szépvölgy, 23.vi.1975, OMB leg. (Nádai 2006); “Pest“ [currently eastern part of Budapest], no other data, 1 ♀ in coll. NMEG (Hillert et al. 2016).

**Southern Great Plain (Dél-Alföld)**, Csongrád-Csanád County, Szeged – Kiskundorozsma env., Nagyszék, 16.–23.vi.1989, 1 spec., pitfall trap with ethylene glycol, Béla Gaskó leg., coll. MMSH (Nádai 2006; Gaskó 2008); Békés County, Bélmegyer env., Fáspuszta, 46°53'42.19"N, 21°11'8.55"E, 1967, 1 spec., at light, no other data (Merkl et al. 2014; Merkl 2015; details specified by TDS pers. comm., 2021); Békés County, Dombegyház env., Trianon border mound, 46°18'17.54"N, 21°8'43.38"E, 99 m a.s.l., 9.vi.2013, 1 ♂, pitfall trap on a narrow strip of grass with loess soil, TDS and TDB leg., coll. HNHM (Merkl et al. 2014; details specified by TDS pers. comm., 2021); Bács-Kiskun County, “Peszér” [= Kunpeszér] env., no other data (Frivaldszky 1865; Endrődi 1957); Bács-Kiskun County, Csávoly, 10.vi.1999, 1 spec., at light, collector not specified (Nádai 2006; Merkl 2015).

**Northern Hungary (Észak-Magyarország)**, Heves County, Mátra Mts, Galyatető, 10.vii.1959, Sándor Szabó leg. (Nádai 2006); Borsod-Abaúj-Zemplén County, Agg­telek National Park, Jósvafő env., 48°29'46"N, 20°33'53"E, 300 m a.s.l., 8.vii.1980, 1 spec., Iván Gyulai leg. (Nádai 2006; data specified by OMB pers. comm., 2020 and SBP pers. comm., 2021).

**Northern Great Plain (Észak-Alföld)**, Hajdú-Bihar County, “Debreczen” [= Debrecen] env., ca. 1860–1880, 1 spec., József Török leg. (Török 1882); Hajdú-Bihar County, Debrecen, 10.vii.1958, 2 spec., Imre Tatár leg., coll. ZUDH (Enyedi 2006); Szabolcs-Szatmár-Bereg County, “Szabolcs vármegye” [= Szabolcs County], no other data (Szlabóczky and Borbás 1900).

**Material examined and new observations**

**Western Transdanubia (Nyugat-Dunántúl)**, Győr-Moson-Sopron County, Győr – Likócs env., ca. 47°42'52.5"N, 17°41'45"E, 2019, 115 m a.s.l., pitfall traps, no other data (see unpublished report for the Fertő-Hanság National Park Directorate, Anonymus 2019); Győr – Győrszentiván env., 47°42'42.17"N, 17°46'25.05"E and 47°42'51.18"N, 17°46'40.77"E, 110 m a.s.l., 10.vi.2016, 5 spec., pitfall traps, CSS and PKB leg. [storage of the specimens not specified]; 47°42'51.04"N, 17°46'40.24"E, 112 m a.s.l., 25.v.2019, 1 ♂, pitfall trap, CSS and PKB leg. [storage of the specimen not specified].

**Central Transdanubia (Közép-Dunántúl)**, Fejér County, Csór, ca. 150 m a.s.l., 21.v.2014, 1 ♀, at light on a steppe, MPK leg., coll. DCO; 28.v.2016, 1 ♂, at light, DVZ obs.; Fejér County, Csákberény, Bucka hill, 47°20'51.65"N, 18°21'35.32"E, 230 m a.s.l., 11.vi.1987, 1 spec., at light, CSB obs.; Fejér County, Gánt env., Köves-völgy [valley], 47°24'19.94"N, 18°22'47.67"E, 280 m a.s.l., 14.vi.2019, 1 ♀ flying after sunset, VSI obs.; Fejér County, Nagykarácsony, 46°52'49.4"N, 18°43'27.1"E, 150 m a.s.l., 2.vi.2021, 2 ♂♂ and 1 ♀ FSLG after sunset, 17–18 °C, light breeze, TDS obs.; Fejér County, Adony env., 47°5'17.2"N, 18°49'10.3"E, 120 m a.s.l., 1.vi.2021, 10 spec. FSLG after sunset, 11–15 °C, no wind, TDB and TDS obs.; Komárom-Esztergom County, Környe, no other data, 1 ♂ in coll. RBIN; Komárom-Esztergom County, Esztergom env., Kis-Strázsa-hegy hill, 47°44'59.210"N, 18°44'35.07"E, 210 m a.s.l., 23.iv.2006, 1 spec., at light (mercury-vapor lamp), VPB; Komárom-Esztergom County, Kesztölc env., 47°43'13.4"N, 18°47'43.3"E, 17.x.2014, 260 m a.s.l., 1 ♀ excavated from its burrow from a depth of 60 cm, loess steppe with abundant occurrence of *Lethrusapterus* (Laxmann, 1770), TVP (for incomplete data on this record see Hillert et al. 2016); Komárom-Esztergom County, Máriahalom env., Siklóernyő-hegy hill, 47°37'38.57"N, 18°41'20.38"E, 214 m a.s.l., 11.vi.2019, 1 ♂ and 1 ♀ flying after sunset, VSI obs. (♂ in coll. HNHM); Komárom Esztergom County, Máriahalom env., 47°37'28.3"N, 18°41'21.68"E, 190 m a.s.l., 31.vii.2020, 1 ♀, at light, BKL obs. + photo (DJP det.); Veszprém County, Nagyvázsony env., 47°1'40.73"N, 17°42'38.62"E, 315 m a.s.l., 12.vi.2009, 1 ♀ flying ca. 10 cm above the ground after sunset, KLP; 16.vi.2016, 3 ♂♂ FSLG after sunset, JHH, JPP, JSUMSZ, MPV and PIL obs.; Veszprém County, Vászoly env., Öreg-hegy, 250–290 m a.s.l., 3.vii.1999, 1 spec., IRB leg., coll. SZM; Veszprém County, Örvényes, 46°55'8.3"N, 17°48'26.07"E, 150 m a.s.l., 16.vi.2019, 1 ♀ flying after sunset, forest pasture, VSI obs.; Veszprém County, Felsőörs, Öreg-hegy, 47°0'57.59"N, 17°58'52.72"E, 214 m a.s.l., 7.viii.2018, 1 ♂, dead near the light in a garden, FKD obs. + photo (DJP det.); Veszprém County, Bakony Mts, Litér, [ca. 200 m a.s.l.], 14.vii.2014, 1 ♀, IRB leg., coll. GML.

**Southern Transdanubia (Dél-Dunántúl)**, Somogy County, Balatonendréd, 46°50'52"N, 17°59'18"E, 174 m a.s.l., 11.v.1989, 1 ♀ excavated from its burrow together with 1 ♂ of *Od.armiger*, VRH; Somogy County, Ságvár, Jaba-völgy [valley], 46°49'28.29"N, 18°2'32.93"E, 180 m a.s.l., 25.ix.2017, 1 ♀ crawling on the ground, PFS obs. + photo (DJP det.); Somogy County, Balatonvilágos – Balatonaliga, 10.vi.1983, 1 spec., at light, SIB obs.; Baranya County, Zselic Mts, Mozsgó, ca. 150 m a.s.l., 27.vii.2017, 1 ♂, at light, MRM; Baranya County, Drávaszabolcs, 4/c Köztársaság tér Street, 45°48'20.95"N, 18°12'43.74"E, 91 m a.s.l., 28.vi.2020, 1 ♀ dead under the lamp, JST; Baranya County, Villányi-hegység Mts, Nagyharsány env., Szársomlyó hill, ca. 145 m a.s.l., 22.v.1977, 1 ♂, at light., AUP; Baranya County, Erdősmecske, ca. 240 m a.s.l., 18.viii.2012, 1 spec., 31.vii.2016, 1 spec., 27.v.2017, 1 spec., REE obs.

**Central Hungary (Közép-Magyarország)**, Pest County, Zsámbék, June 2016, 1 ♀, students of Department of Zoology, Charles University, Prague leg., coll. DKP deposited in NMPC; Pest County, Biatorbágy, 47°27'54.501"N, 18°51'0.515"E, ca. 190 m a.s.l., 24.vii.2021, 1 ♂, at light, GAB obs. + photo (DJP det.); Pest County, Nagymaros env., Rigó-hegy hill, 47°46'31.63"N, 18°56'11.65"E, ca. 300 m a.s.l., 21.iv.2019, 1 ♂, night sweeping, TNB leg., coll. HNHM; Pest County, Szentendre – Izbég env., 47°41'47.61"N, 19°1'40.06"E, 195 m a.s.l., 9.vi.2014, 1 spec., at light (mercury-vapor lamp), GBP and APE obs.; Pest County, Pócsmegyer env., 47°43'44.5"N, 19°6'25.7"E, 110 m a.s.l., 11.viii.2006, 1 spec., 20.vi.2008, 1 spec., 18.vi.2010, 1 spec., pitfall traps without attractant, SBP and ZBP leg., 16.ix.2014, 1 ♀, pitfall trap, SBP leg. [storage of the specimens unspecified]; Pest County, Pomáz env., Szamár-hegy hill, 47°39'28.7"N, 18°58'43.06"E, ca. 185 m a.s.l., 2.vii.2019, 1 ♂ flying after sunset, VSI obs.; Pest County, Pomáz, Majdánpola, 47°38'27.7"N, 19°0'18.61"E, 190 m a.s.l., 1.viii.2019, 1 ♂ at light (mercury-vapor lamp), SIB obs., 1 m FSLG after sunset, VSI leg., coll. HNHM; Pest County, Budakeszi env., Hosszú-dűlő, 200 m a.s.l., 5.vi.1991, 2 ♂♂ and 1 ♀, *Cynodonto-Festucetum*, on *Glomusmacrocarpum*, LAB leg., coll. GML (pair) and JMB (1 ♂); Pest County, Budakeszi, 5.vi.2013, 1 spec., 6.vii.2014, 1 spec., 19.vii.2014, 1 spec., 26.vii.2014, 1 spec., 3.vi.2015, 1 spec., 28.v.2016, 1 spec., 4.vi.2018, 1 spec., all at light, SIB obs.; Pest County, Budakeszi, gliding airport, ca. 200 m a.s.l., 5.vii.1991, 1 spec., LNB leg., coll. SZM; Pest County, Budakeszi, Farkas-hegy env., gliding airport, 47°28'39.7"N, 18°54'50"E, ca. 200 m a.s.l., 6.v.2018, 2 ♂♂ flying after sunset, TNB obs. (1 ♂ in coll. HNHM); 23.v.2019, 1 m flying after sunset, 12.vi.2019, 1 ♀, night sweeping, 17.vi.2019, 1 ♂, night sweeping, 27.vi.2019, 1 ♂, night sweeping, 2.vii.2019, 1 ♀ flying after sunset, TNB obs., 22.ix.2019, 1 spec., TNB obs., 47°28'55.2"N, 18°55'6.25"E, 18.vi.2018, 2 ♂♂ and 1 ♀ flying after sunset, 20.vi.2018, 4 ♀♀ flying after sunset, 5.vii.2018, 2 ♂♂ and 3 ♀♀ flying after sunset, 10.vii.2018, 2 ♂♂ flying after sunset, 12.ix.2018, 1 ♂ excavated from its burrow, TNB leg., coll. HNHM; Pest County, Budakeszi – Nagyszénászug, ca. 47°29'11.6"N, 18°55'26.3"E, ca. 230 m a.s.l., 18.vi.2018, 1 spec., 9.vi.2019, 3 spec. in a private garden, LMB obs.; Pest County, Budaörs env., Farkas-hegy, 47°28'27.29"N, 18°56'40.42"E, ca. 335 m a.s.l., 8.vi.2019, 1 spec., OMB obs.; 22.vi.2021, 1 ♂ FSLG after sunset, VSI obs.; Pest County, Törökbálint, Nagy-Mező, 47°25'31.01"N, 18°57'31.04"E, 216 m a.s.l., 18.vi.2019, 1 ♀ flying after sunset, VSI obs.; Budapest, Tétényi-fennsík env., 47°25'2.309"N 18°58'59.332"E, 180 m a.s.l., 6.viii.2021, 1 ♂, at light, MLB obs. + photo, DJP det.; Budapest, “Pest” [currently eastern part of Budapest], no other data, 2 spec in coll. MNHN; Újpest [currently part of Budapest], undated, 1 ♂, Robert Meusel [leg.], coll. Jože Staudacher deposited in PMSL; Budapest, no other data, 7 spec. in coll. NHMW, 2 ♂♂ and 1 ♀ (ex original coll. Josef Breit, Vienna) in coll. Georg Frey deposited in NHMB, 1 ♂ (ex original coll. Josef Breit, Vienna) in coll. Jacques Baraud deposited in MNHN, 1 ♂ in coll. MNBG, 1 ♂ in coll. DKC; Budapest, undated, [Hugó] Diener [leg.], 2 ♂♂ and 1 ♀ (ex original coll. Josef Breit, Vienna) in coll. Georg Frey depo­sited in NHMB, 3 spec. in coll. ZSMG, 1 spec. in coll. SMNK, 1 ♂ in coll. DKC; Budapest, Ofen [= Buda], undated, [E.] Merkl [leg.], 2 ♂♂ and 2 ♀♀ (ex coll. Stöcklein) in coll. Georg Frey deposited in NHMB; “Buda-Pesth” [= Budapest], undated, 1 ♂ and 1 ♀, E. Merkl leg., coll. NMPC; Budapest, 1890, “coll. O. Leonhard”, no other data, 2 ♂♂ in coll. SDEI; Budapest, 1895, 1 ♂ and 1 ♀, [Hugó] Diener [leg.], coll. SDEI; Budapest, 1899, 2 ♀♀, [Hugó] Diener [leg.], coll. MSNG; Budapest, Hármashatárhegy Airfield, 47°33'11.133"N, 18°58'29.279"E, 276 m a.s.l., 7.vi.2019, 1 spec., NPB obs.; Pest County, Dunakeszi, gliding airport, 47°36'51.79"N, 19°8'55.91"E, 125 m a.s.l., 10.vi.2019, 1 ♀ flying after sunset, VSI obs.; Pest County, Bugyi env., Nemes-ürbő, ca. 47°10'55.9"N, 19°11'24.7"E, 92 m a.s.l., 7.vii.2018, 3 spec., Hunor Győrfy obs.; Pest County, Bugyi, Ürbőpuszta, 47°9'52.47"N, 19°10'21.22"E, 91 m a.s.l., 10.vi.2019, 1 ♂ flying after sunset, VSI obs.; Pest County, Tatárszentgyörgy env., Ordító, ca. 47°2'13.8"N, 19°17'40.2"E, ca. 95 m a.s.l., 5.vii.1999, 4 spec., AMK obs.; Pest County, Tatárszentgyörgy env., Rohanka-dűlő, 47°3'48.05"N, 19°20'26.47"E, 98 m a.s.l., date not available [end of 20^th^ or beginning of 21^st^ century], 1 spec. flying after sunset, AMK obs.; Pest County, Tatárszentgyörgy env., Szabad-rét, ca. 47°3'14.07"N, 19°18'1.37"E, 94 m a.s.l., 29.vi.2018, 1 spec., CVK obs.; Pest County, Tatárszentgyörgy env., Széna-dűlő, ca. 47°1'42.25"N, 19°17'26.7"E, ca. 100 m a.s.l., 21.vi.1998, 3 ♀♀, AMK obs.; Pest County, Nagytarcsa env., Küdői-hegy hill, 47°32'21.43"N, 19°19'11.77"E, 230 m a.s.l., 8.vi.2003, 1 ♂ and 2 ♀♀, at light (mercury-vapor lamp), VSI obs. (1 spec. in coll. HNHM), 47°32'13.92"N, 19°19'10.72"E, 21.iv.2006, 1 ♀, at light (mercury-vapor lamp), VSI obs., 47°31'59.96"N, 19°19'22.96"E, ca. 250 m a.s.l., 19.vi.2018, 1 ♂, night sweeping, VSI leg., coll. HNHM, 1 ♀, at UV light, SIB obs., 47°32'17.59"N, 19°19'16.03"E, 18.vi.2013, 1 ♂ and 2 ♀♀, at light (mercury-vapor lamp), 25.vii.2019, 1 ♀, at UV light, VSI obs.; Pest County, Csomád, Öreg-hegy, 47°39'29.88"N, 19°12'38.05"E, 15.vi.2002, 1 ♂ and 1 ♀, at light (mercury-vapor lamp), VSI obs. (♂ in coll. HNHM); Pest County, Gödöllő, 55 Erdőszél Street, 47°36'11.3"N, 19°23'23.6"E, 250 m a.s.l., 15.vi.2004, 1 ♂, at light (mercury-vapor lamp), VSI leg., coll. HNHM, 5.viii.2004, 1 ♀, 7.viii.2004, 1 ♀, at light (mercury-vapor lamp), VSI obs.; Pest County, Gödöllő env., Faház-tető hill, 47°37'10.12"N, 19°25'8.94"E, 255 m a.s.l., 19.v.2004, 1 m and 2 ♀♀, at light (mercury-vapor lamp), VSI obs. (1 f in coll. HNHM), 47°37'5.81"N, 19°25'9.68"E, 26.vi.2017, 1 ♀, at light (mercury-vapor lamp), VSI, TNB and AKB obs.; Pest County, Gödöllő env., Perőc-oldal, 47°34'5.754"N, 19°20'8.424"E, ca. 250 m a.s.l., 30.vi.2019, 1 ♀, Csanád Szénási leg., coll. HNHM; Pest County, Vác­kisújfalu, Szélesek, 47°42'40.24"N, 19°19'36.32"E, 180 m a.s.l., 24.vii.2018, 1 ♀ flying after sunset, VSI obs.; Pest County, Pest County, Galgamácsa env., Ecskendi Forest, Ördög-árok area, 47°44'20.32"N, 19°25'17.34"E, 235 m a.s.l., 5.vi.2015, 1 ♀, at light (marcury-vapor lamp), VSI obs.; Pest County, Domonyvölgy, Bárányjárás, 47°37'23.8"N, 19°24'1.94"E, 220 m a.s.l., 21.v.2004, 1 ♂ and 1 ♀, at light (mercury-vapor lamp), VSI obs. (1 spec. in coll. HNHM); Pest County, Gödöllő - Máriabesnyő env., 47°35'38.59"N, 19°24'4.82"E, ca. 190 m a.s.l., 13.vi.2013, 1 ♂, ZKB obs. + photo (DJP det.); Pest County, Isaszeg, 29 Erdő Street, 47°31'23.412"N, 19°23'33.87"E, 19.vi.2003, 1 ♂, at light, VSI obs.; Pest County, Isaszeg env., Szarkaberki-völgy [valley] 47°32'14.86"N, 19°22'11.26"E, ca. 210 m a.s.l., 27.vi.2019, 2 ♂♂ and 1 ♀ flying after sunset, VSI obs., 1 ♂, at UV light, SIB leg., coll. HNHM; 23.vi.2020, 10 spec. SIB obs.; 1.vii.2020, 1 spec., at light, SIB obs.; Pest County, Isaszeg env., Kőmalmi tölgyes, 47°33'51.65"N, 19°25'48.93"E, ca. 250 m a.s.l., 9.v.2004, 1 ♀, at light (mercury-vapor lamp), VSI leg., coll. HNHM; Pest County, Dabas, 20.v.2012, 1 spec., at light, SIB obs.; Pest County, Pécel, 5.vi.2018, 1 spec., at light, SIB obs.; Pest County, Pécel env., 47°29'49.85"N, 19°22'56.56"E, ca. 200 m a.s.l., 12.vi.2010, 1 ♂, at light (mercury-vapor lamp), JDB obs.; Pest County, Pécel env., Trianoni-emlékmű, 47°28'28.86"N, 19°22'10.78"E, ca. 255 m a.s.l., 15.iv.2015, 1 spec., LNB obs.; Pest County, Csévharaszt, 24.vi.2004, 1 spec., at light., SIB obs.; Pest County, Albertirsa env., Golyófogó-völgy [valley], 47°15'52.86"N, 19°37'59.73"E, 150 m a.s.l., 1.vii.2019, 2 ♂♂ flying after sunset, SIB and VSI obs., 2 ♀♀, at UV light, SIB obs. (1 ♀ in coll. HNHM); Pest County, Tóalmás, Boldogkáta-puszta, 47°30'22.77"N, 19°42'2.44"E, 110 m a.s.l., 28.vi.2019, 1 ♂ and 1 ♀ flying after sunset, VSI obs. (♀ in coll. HNHM), 1 spec., at light, SIB obs.; Pest County, Tápióbicske, Gombai-patak [stream] bank, 47°22'12.5"N, 19°38'43.6"E, 120 m a.s.l., 3.vii.2019, 1 ♂ flying after sunset, VSI obs.; Pest County, Tápióbicske, Felső-Tápió [stream] bank, 47°23'58.92"N, 19°41'25.29"E, 111 m a.s.l., 20.vii.2020, 1 ♂ flying after sunset, VSI obs.

**Southern Great Plain (Dél-Alföld)**, Bács-Kiskun County, Kunpeszér env., Alsó-Peszéri-rétek, ca. 47°3'50.129"N, 19°17'57.59"E, 93 m a.s.l., 8.vi.1996, 2 ♂♂, 23.vi.1998, 1 spec., 10.vi.2002, 2 ♂♂ and 1 ♀, at light, AMK obs.; Bács-Kiskun County, Kunpeszér env., Peszéri-erdő forest, ca. 100 m a.s.l., 6.vi.1998, 6 spec., 21.vi.1999, 3 spec., 30.vi.1999, 2 ♂♂, 11.vii.1999, 1 spec., AMK obs.; 26.vi.2018, 2 spec., at light, REE obs.; 28.vi.2018, 3 spec., at light, REE and CVK obs.; 4.vii.2018, 4 spec., 5.vii.2018, 7 spec., at light, REE obs.; 6.vii.2018, 1 spec., CVK obs.; 9.vii.2020, 1 spec., 13.vii.2020, 1 spec., at light, REE obs.; 22.vii.2020, 1 spec., 29.vii.2020, 1 spec., 30.vii.2020, 5 spec., 31.vii.2020, 2 spec., 8.viii.2020, 1 spec., 16.viii.2020, 10 spec., 18.viii.2020, 7 spec., 19.viii.2020, 6 spec., 20.viii.2020, 8 spec., Botond Kozma obs.; Bács-Kiskun County, Kunadacs env., Hunga­rian meadow viper Conservation Centre, ca. 47°1'27.807"N, 19°17'21.286"E, ca. 100 m a.s.l., 29.vi.2018, 1 spec., Vadász Csaba obs.; Bács-Kiskun County, Kunadacs env., Hetvenholdas, ca. 47°0'56.34"N, 19°16'54.34"E, ca. 97 m a.s.l., 27.ix.2016, 1 ♀ FSLG after sunset, AMK obs.; Bács-Kiskun County, Kunadacs, Nagy-erdő forest, date not available [21^st^ century], 1 spec. caught after sunset, AMK obs.; Bács-Kiskun County, Kunadacs, Peregi-dűlő, ca. 46°57'0.4"N, 19°17'29.4"E, 95 m a.s.l., 6.vi.2006, 1 spec., AMK obs.; Bács-Kiskun County, Kunadacs env., Szabadszállási-legelő, ca. 46°56'6"N, 19°18'15"E, 94 m a.s.l., 9.vi.2006, 1 spec., AMK obs.; Bács-Kiskun County, Páhi, Páhi-rétek, ca. 100 m a.s.l., 10.vii.2020, 3 spec., CBK and REE obs.; Bács-Kiskun County, Kiskunhalas env., pasture, 46°24'10.97"N, 19°30'33.08"E, 122 m a.s.l., 7.vi.2021, 2 ♂♂ and 1 ♀, at light just after sunset, together with 1 ♀ of *Od.armiger*, TKK obs. + photo (DJP det.); Bács-Kiskun County, Kecskemét – Hunyadiváros, 46°55'6.114"N, 19°42'43.794"E, 115 m a.s.l., 29.vi.2021, 1 ♀, at light, BCK and KVB obs. + photo (DJP det.).

**Northern Hungary (Észak-Magyarország)**, Nógrád County, Kozárd, village area, 47°54'53.31"N, 19°37'7.07"E, 180 m a.s.l., 28.vii.2020, 1 ♀, at light (wall lamp of a residential house), KHE obs.; Nógrád County, Kozárd env., Majorsági-hegy hill, 47°54'59.87"N, 19°36'37.52"E, ca. 240 m a.s.l., 1.viii.2020, 1 ♀ flying after sunset, KHE obs.; Nógrád County, Kozárd env., Pohánka hill, 47°54'56.21"N, 19°37'29.88"E, 225 m a.s.l., 29.vii.2020, 1 ♂ and 1 ♀ flying after sunset, KHE obs.; Nógrád County, Bátonyterenye – Kisterenye, Váci Mihály Street, 48°0'32.22"N, 19°49'46.48"E, 190 m a.s.l., 28.v.1978, 1 ♂ FSLG after sunset, TKB and TKG obs.; Heves County, Tarna­lelesz env., Pataji-far, 48°7'32.66"N, 20°9'32.12"E, 475 m a.s.l., 9.vi.2016, 1 ♂, in the grass during the day, shrubby edge of an oak forest (*Quercuscerris*), CBE obs.

**Comment**

**Figure 12.**
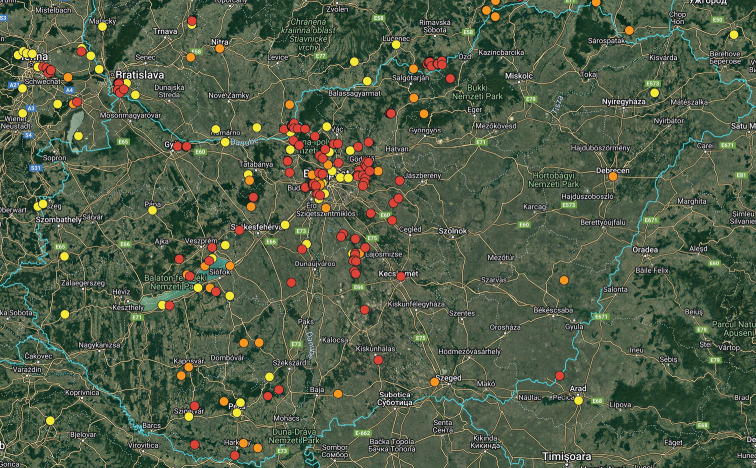
Distribution of *B.unicornis* in Hungary (yellow circles – records before 1950, orange circles – records between 1950–1999, red circles – records after 1999).

Approximately one-third of the known localities of the species are located in Hungary. It is known here from 18 of the 19 counties. The first record from Hungary, without further details, is given by Illiger (1800). The first dated Hungarian record is from the vicinity of Buda (Graberl, a historical excursion destination) from 1798, only nine years after the species was described (Horváth 1884). Old records are summarised by Endrődi (1957). *Bolbelasmusunicornis* has been recorded several times as food for some birds (*Cuculuscanorus*, *Falcovespertinus*, and *Upupaepops*) in several localities of the Austro-Hungarian Empire, including two Hungarian, two Slovak, and one Romanian locality (Csiki 1904, 1905, 1910; Madon 1930; Keve and Szijj 1957). Newer Hungarian records are summarised by Nádai (2006). Data collected by Duna-Ipoly National Park are now available online (Duna-Ipoly National Park 2021). This study presents as yet unpublished records from 68 Hungarian localities. For the distribution of the species in Hungary see Fig. 12.

### ﻿Slovenia

**Published data**

**Carniola (Kranjsko)**, “Carniolia, *Bolbocerasquadridens* Fabr.”, undated, 1 ♂, Ferdinand Joseph Schmidt leg., coll. F. J. Schmidt deposited in PMSL, Savo Brelih revid. (Brelih et al. 2010; sex supplemented by Tomi Trilar pers. comm., 2021); note: Trilar (2019) reported that there is another specimen of *B.unicornis* in the Schmidt’s collection in PMSL labelled “*Athyreuskordofanus*”, but the photograph makes it clear that it is a member of the genus *Athyreus*; Bohinjska Bela, 1 spec. with no other data in coll. JHIS (Brelih et al. 2010; Vrezec et al. 2011); Sorško polje, June 1900, 1 spec., Mate Hafner leg., coll. JHIS, Alja Pirnat revid. (Brelih et al. 2010; Vrezec et al. 2011).

**Styria (Štajerska)**, “Leonhard” [= Lenart v Slovenskih goricah], no other data, 1 spec. J. N. Spitzy leg. et coll. (Brancsik 1871; Horion 1958; Brelih et al. 2010; Vrezec et al. 2011); “Marburg, Styria” [= Styria, Maribor], undated, 2 ♂♂ and 1 ♀ [Josef] Peyer [leg.], coll. J. Peyer deposited in PMSL (Brelih et al. 2010; Vrezec et al. 2011; data supplemented and specified by Tomi Trilar pers. comm., 2021).

**Comment**

Although there are many localities suitable for the species in Slovenia, only five old records are known from this country. The species is most likely still present here, and the reason for the absence of new data is probably the low collecting activity of the local entomologists and/or the ignorance of appropriate monitoring methods for the species. Also, a recent attempt to rediscover the species in Slovenia (Pirnat 2009) was unsuccessful mainly due to the use of inappropriate collecting methods.

### ﻿Croatia

**Published data**

**Croatia proper (Središnja Hrvatska)**, Moslavina [a microregion between the rivers Lonja in the south and west, Česma in the north and Ilova in the east], no other data [19^th^ century] (Schlosser Klekovski 1878); Koprivnica-Križevci County, Križevci env., no other data [19^th^ century] (Schlosser Klekovski 1878).

**Slavonia (Slavonija)**, Osijek-Baranja County, Osijek env., no other data [19^th^ century], Vukas [leg.] (Schlosser Klekovski 1878); Vukovar-Syrmia County, Vinkovci, promenade near the Bosut river, 80 m a.s.l., 1.vi.1895, 1 ♀, caught with a net, collector not specified (Koča 1906; Mikšić 1959, 1960, 1970); Vukovar-Syrmia County, Gradište env., 45°10'33.7"N, 18°44'54.7"E, mixed lowland forest, 81 m a.s.l., 5.vi.2014, 1 ♂, at light, collector not specified (Koren 2017).

**Material examined**

“Chorvatsko” [= Croatia in Czech language], 1 ♂, “ex. coll. E. Hachler”, no other data, coll. MMBC.

**Dalmatia (Dalmacija)**, “Dalmatia”, no other data, 1 ♀ in coll NMPC; “Dalmat.” [= Dalmatia], no other data, 1 ♀ in coll. NMPC.

**Comment**

In Croatia, the species is known only from four old records from the 19^th^ century. The only recent record (Gradište) is given by Koren (2017). Further historical undated specimens deposited in MMBC and NMPC are presented in this study.

### ﻿Bosnia and Herzegovina

**Published data**

“Herzegovina”, no other data, 1 ♂ in coll. ZFMK (Hillert et al. 2016).

**Federation of Bosnia and Herzegovina (Federacija Bosne i Hercegovine)**, Zavidovići env., Gostović river valley, no other data, Károly Kendi leg. (Kendi 1910); Sarajevo, no other data (Mikšić 1953, 1958, 1960, 1970; Lelo 2006; Lelo and Kašić-Lelo 2010; Koren 2017).

**Federation of Bosnia and Herzegovina (Federacija Bosne i Hercegovine)** or **Republika Srpska** (Република Српска), Babin potok [river], no other data, 1 spec. in coll. René Mikšić [currently deposited in CMZC] (Mikšić 1953, 1958, 1960, 1970; Lelo 2006; Lelo and Kašić-Lelo 2010; Koren 2017).

**Comment**

Only four old records from Bosnia and Herzegovina have been published. No recent findings are known.

### ﻿Serbia

**Published data**

**Vojvodina (Војводина)**, Srem District (Сремски округ), Mt. Fruška gora (Фрушка гора), village of Vrdnik (Врдник), June 2016, 1 ♀, at light, collector unknown, coll. DKP deposited in NMPC (Ćurčić et al. 2019); Srem District (Сремски округ), Ruma (Рума), undated, 1 ♂, [Harald] Schweiger leg., coll. MSNG (Arnone and Massa 2010; Hillert et al. 2016; collector’s name specified by Roberto Poggi pers. comm., 2021); Inđija (Инђија) env., Krčedin (Крчедин), 1.vii.2013, 1 ♂, at light at 21.25 CEST, ZBB obs. + photo (Ćurčić et al. 2019; for more detailed data on this record see Material examined and new observations below); South Bačka District (Јужнобачки округ), South Bačka District (Јужнобачки округ), Mt. Fruška gora (Фрушка гора), Sremski Karlovci (Сремски Карловци) env., Stražilovo (Стражилово), 14.vii.2005, 1 ♀, at light, Dejan Stojanović obs. (Gavrilović and Stojanović 2008; Ćurčić et al. 2019); South Banat District (Јужнобанатски округ), Deliblato Sands (Делиблатска пешчара), Deliblato (Делиблато) env., Jagoda (Јагода), ca. 44°53'33"N, 21°3'2.6"E, date not specified, Zoran Gradojević leg. (Gradojević 1963; Ćurčić et al. 2019).

**Belgrade District (Град Београд)**, Mala Ivanča (Мала Иванча) env., Grkovo (Грково), Trešnja Forest (Шума Трешња), 14.v.1986, 1 ♀ dug up beneath a hazel shrub together with *Tuber* fungi DPB leg. et coll. (Ćurčić et al. 2019); Mt. Kosmaj (планина Космај), Tresije Monastery (Манастир Тресије), 21.vi.2003, 1 ♀, dead under the lamp near a restaurant, DPB leg. et coll. (Ćurčić et al. 2019).

**Southern and Eastern Serbia (Јужна и источна Србија)**, Bor District (Борски округ), Đerdap National Park (Национални парк Ђердап), 6 km WSW of Tekija (Текија), 27.–28.v.2014, 2 ♂♂ and 1 ♀, collector not specified, coll. DKP [deposited in NMPC] (Hillert et al. 2016; for details on this record see Material examined and new observations below); Pirot District (Пиротски округ), Bela Palanka (Бела Паланка) env., Babin Kal (Бабин Кал) env., 43°19'9"N, 22°23'23"E, 750 m a.s.l., 3.vii.2014, 1 ♂, at light, a mea­dow near an oak-hornbeam forest, SBS leg., coll. NMSB (Ćurčić et al. 2019); “Tsaribrod (Цариброд)” [= Dimitrovgrad (Димитровград)], no other data (Nedyalkov 1906; Mikšić 1959); Zaječar District (Зајечарски округ), village of Planinica (Планиница), 28.v.2006, 1 ♀, dug up in the garden, Siniša Ognjenović leg., coll. DPB (Ćurčić et al. 2019).

**Figure 13.**
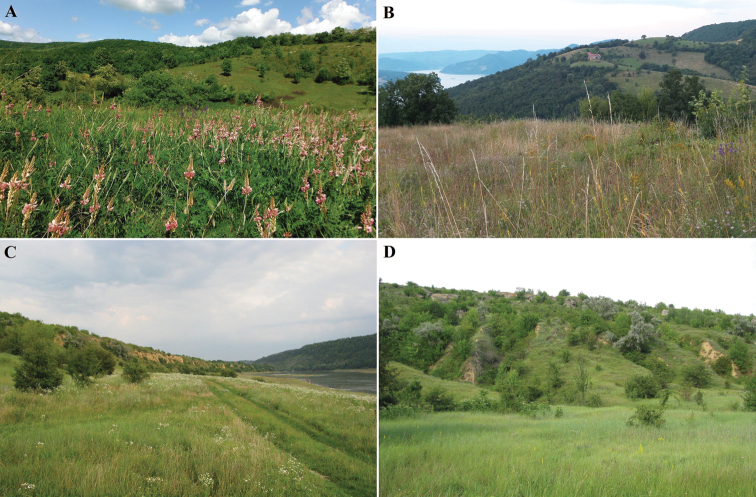
Localities with *B.unicornis***A** Hungary, Kozárd env. (photograph by Krisztián Harmos) **B** Serbia, Đerdap National Park, Tekija env. (photograph by Ivo Martinů) **C, D** Ukraine, Dniester Canyon National Nature Park, Horodok env. (photographs by Yurii V. Kanarskyi).

**Material examined and new observations**

**Figure 14.**
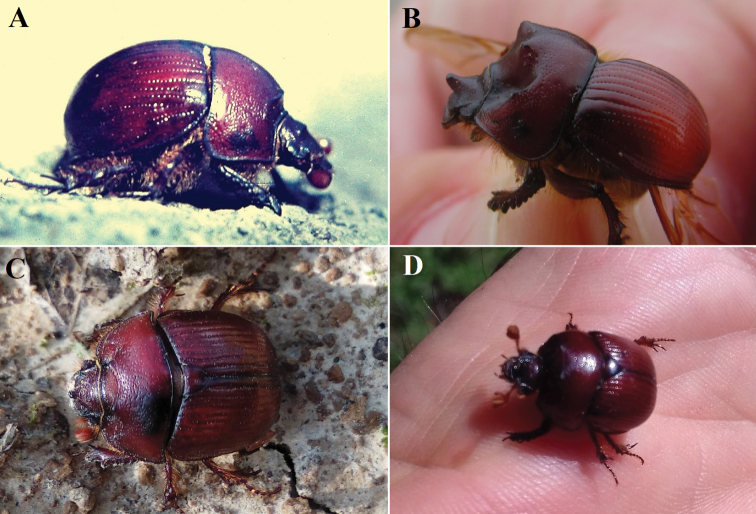
Findings of *B.unicornis***A** Slovakia, Slanská Huta env., 24.vii.1972 (photograph by Zdeněk Laštůvka) **B** Serbia, Bukovac env., Beljevo hill, 27.v.2019 (photograph by Marko Šćiban) **C** Serbia, Leskovo env., 20.vi.2020, (photograph by Miloš Popović) **D** Ukraine, Semyhiria env., 3.vii.2020 (photograph by Dmytro Protopopov).

**Vojvodina (Војводина)**, Srem District (Сремски округ), Inđija (Инђија) env., Krčedin (Крчедин), 19.viii.2006, 2 ♂♂, at light, steppe meadow near the Danube river, LMN leg., coll. RSG and VVO; 45°10'04.5"N, 20°08'15.4"E, 98 m a.s.l., 1.vii.2013, 1 ♂, at light at 21.25 CEST, ZBB obs. + photo (for partial data on this record see Ćurčić et al. 2019); South Bačka District (Јужнобачки округ), Fruška Gora National Park (Национални парк Фрушка гора), Bukovac (Буковац) env., northern slope of Beljevo (Бељево) hill, 45°10'56.579"N, 19°53'0.802"E, 270 m a.s.l., 27.v.2019, 1 ♂, dead on the ground near the road (killed in flight by a passing car), MSN obs. + photo (Fig. 14B).

**Southern and Eastern Serbia (Јужна и источна Србија)**, Bor District (Борски округ), Leskovo (Лесково) env., 44°18'17.28"N, 21°56'54.96"E, ca. 400 m a.s.l., 20.vi.2020, 1 ♂ crawling on the ground near the road at 18.42 CEST, MPN obs. + photo (Fig. 14C); Bor District (Борски округ), Đerdap National Park (Национални парк Ђердап), 6 km WSW of Tekija (Текија), 44°39'19.4"N, 22°20'15.6"E, 300 m a.s.l., 27.v.2014, 1 spec. accidentally dug up while setting pitfall traps for ground beetles, RKP, 27.–28.v.2014, 51 spec. (both sexes in a ratio of 1:1) FSLG at 20.35–21.00 CEST, steppe hillside (probably former pasture, presently with tall vegetation) near an oak-beech forest, DHH (22 spec.), RKP (16 spec.), ZCP (11 spec.), TGK (1 spec.), and PSZ (1 spec.) leg., coll. OSD, DHH, DJP, DKP, GML, LMO, MBF, PSZ, RKP, TGK, VJP and ZCP (for incomplete data on this record see Hillert et al. 2016) (see Table 8 for full data on the flights); 12.–13.vii.2014, 8 ♂♂ and 12 ♀♀ FSLG at 20.43–21.15 CEST, the same place as May 27–28, RKP (11 spec.), IMO (7 spec.), MKJ (2 spec.) leg. et coll., 4 ♂♂ and 3 ♀♀ in coll. GML (see Table 8 for full data on the flights); 17.vi.2018, 1 ♂ and 2 ♀♀ FSLG after sunset, IMO, ZCP.

**Table 8.** Data on flights of adults of *B.unicornis* at the locality of Tekija (for abbreviations see Table 1).

**Serbia, Tekija env.**									
**date**	***n***	**BF**	**EF**	**S**	**S-BF**	**S-EF**	**DF**	**T**	**note**
27.v.2014	27	20.35	21.00	20.03	32 min	57 min	25 min	24 °C	after a period of persistent rainfall
28.v.2014	24	20.35	21.00	20.03	32 min	57 min	25 min	24 °C	dtto
12.vii.2014	9	20.45	21.15	20.16	29 min	59 min	30 min	19 °C	ca 5 hours after the rain; no wind
13.vii.2014	11	20.43	21.14	20.15	28 min	59 min	31 min	22 °C	full moon, clear skies, light breeze, storm in the distance
***n***	**71**	**average**			**30 min**	**58 min**	**28 min**	**22 °C**	

**Table 9.** Data on flights of adults of *B.unicornis* at the locality of Cordenons env. (for abbreviations see Table 1).

Italy, Cordenons env. (Glerean and Stefani 2019)									
**date**	***n*** (♂/♀)	**BF**	**EF**	**S**	**S-BF**	**S-EF**	**DF**	**T**	**note**
9.ix.2018	2 (1/1)	20.10	20.30	19.35	35 min	55 min	20 min	21.5 °C	humidity 81%
16.v.2019	3 (2/1)	21.00	21.15	20.34	26 min	41 min	15 min	17 °C	male crawling on the ground at 21.20 CEST
24.v.2019	5 (5/-)	21.20	21.35	20.43	37 min	52 min	15 min	20 °C	humidity 70%
6.vi.2019	3 (2/1)	21.00	21.20	20.55	5 min	25 min	20 min	22 °C	humidity 70%
***n*** (♂/♀)	**13 (10/3)**	**average**			**26 min**	**43 min**	**18 min**	**20 °C**	

**Comment**

The known distribution of the species in Serbia was summarised by Ćurčić et al. (2019), who listed a total of 12 localities. New data from two of them (Krčedin, Tekija) and from two other new sites (Bukovac, Leskovo) are presented in this study.

### ﻿Albania

**Published data**

**Tirana County (Qarku i Tiranës)**, Sauk, 10.vi.1958, 1 spec., 10.–20.vi.1961, 1 spec., Xhelo Murraj leg. (Murraj 1962); Ibë, 13.v.1959, 1 spec., 8.vi.1962, 1 spec., Xhelo Murraj leg. (Murraj 1962). Note: Murraj stated that he also found *Od.armiger* and *Och.integriceps* at both sites.

**Comment**

From Albania, only these records from two localities near Tirana have been published. Murraj (1962) reported that in Albania, *B.unicornis* is rare in lowland areas up to 700 m a.s.l. None of the editions of the Catalogue of Palaearctic Coleoptera (Král et al. 2006; Nikolajev et al. 2016) lists Albania for this species.

### ﻿Romania

**Published data**

**Crișana**, Arad County, “Újarad” [= Arad – Aradul Nou], 28.iv.1907, 1 spec. inside the digestive system of *Falcovespertinus*, Ernő Csiki obs. (Csiki 1910).

**Transylvania (Transilvania)**, Sălaj County, Zalău env., 3.viii.1973, 1 ♀, forest, collector unknown, coll. OHS (Hillert et al. 2016); Bistrița-Năsăud County, “Bistritz” [= Bistrița], no other data, Müller leg. (Petri 1912; Panin 1957); Bistrița-Năsăud County, Urmeniș, no other data (Panin 1957); Hunedoara County, “Nagyág” [= Săcărâmb], no other data (Bielz 1887; Kuthy 1898; Petri 1912; Endrődi 1957; Panin 1957); Sibiu County, “Mediasch” [= Mediaș], no other data, Prof. Fabini leg. (Fuss 1858), no other data, Eduard Albert Bielz leg. (Bielz 1887; Kuthy 1898; Petri 1912; Endrődi 1957; Panin 1957); Sibiu County, “Nagyszeben” or “Hermannstadt” [= Sibiu], no other data (Kuthy 1898; Petri 1912; Endrődi 1957; Panin 1957); Sibiu County, “Szenterzsébet” [= Sibiu – Gușterița], no other data (Endrődi 1957); Cluj County, “Kolozsvár” [= Cluj-Napoca], no other data (Endrődi 1957); Cluj County, “Szamosújvár” [= Gherla], no other data, Ormay leg. (Kuthy 1898; Petri 1912; Endrődi 1957; Panin 1957); Cluj County, Stufărișurile de la Sic Nature Reserve env., 2002–2004, 6 spec., forest edge, no other data (Niţu 2007; Ruicănescu and Niţu 2008; Anonymus 2015); Mureș County, “Schässburg” [= Sighișoara or Segesvár], no other data, Karl Petri leg. (Petri 1912; Endrődi 1957; Panin 1957), Sighișoara, Târnava Mare river, no other data (Ruicănescu and Niţu 2008; Tatole et al. 2009).

**Western Moldavia (Moldova Occidentală)**, Suceava County, “Mihoweny” [= Mihoveni], 1 ♂ with no other data (Jasilkowski 1906); Vaslui County, Zorleni, no other data (Fleck 1905; Panin 1957).

**Banat**, Caraș-Severin County, “Gerník” [= Gârnic] env., 44°45'36.72"N, 21°46'29.48"E, 620 m a.s.l., 11.–13.vi.2016, ca. 14 spec. fyling low above the ground after sunset, together with tens of spec. of *Od.armiger*, air temperature 12–15 °C, JHH, JPP, JSUMSZ, MPV and PIL obs. (Spružina 2016; data specified and corrected by JHH and JSU pers. comm., 2021).

**Muntenia**, Giurgiu County, Comana, no other data, Arnold Lucien Montandon leg., Jules Bourgeois det. (Montandon 1906); Bucureşti env., no other data (Mano­lache 1930).

**Dobruja (Dobrogea)**, Tulcea County, Babadag [env.], [100–200 m a.s.l.], 1989–2000, no other data (Niţu 2001); Constanța County, Albești env., Hagieni Forest, ca. 50 m a.s.l., no other data, L. Székely pers. comm., 2014 (Fusu et al. 2015).

**Material examined and new observations**

**Transylvania (Transilvania)**, Sibiu County, “Transsylv. Alpen” [= Transsilvanische Alpen (Carpații Meridionali)], “R.Turm Paſs” [= Roter-Turm-Pass (Pasul Turnu Roșu)], 350–450 m a.s.l., 1917, 1 ♂ and 1 ♀, Dr Maertens [leg.], coll. MNBG; Cluj County, Suatu, ca. 46°46'39"N, 23°58'24"E, ca. 365 m a.s.l., August 1997, 1 ♀, at light, steppe hillside with sparsely scattered oak trees, ARC (for incomplete data on this record see Ruicănescu and Niţu 2008); Sibiu County, Șura Mare, [ca. 450 m a.s.l.], 28.vii.1972, 1 ♂ and 2 ♀♀, E[ckbert] Schneider [leg.], coll. Eckbert Schneider deposited in BNMS; Sibiu County, “Hammersdorf” [= Sibiu – Gușterița], [ca. 425 m a.s.l.], 17.vi.1888, [Mauritius von] Kimakowicz [leg.], coll. BNMS.

**Western Moldavia (Moldova Occidentală)**, Bacău County, Comănești (ca. 46°25'38.5"N, 26°26'31.1"E), July 2004, 1 ♂, dead inside the collector’s house (pro­bably attracted by the light), APC; 31.vii.2010, 1 ♀, at light, APC; 8.viii.2011, 1 ♂, Barber pitfall trap, APC leg., coll. CMI; Iași County, Hârlău env., Pîrcovaci env., 47°28'28.29"N, 26°47'22.17"E, 240 m a.s.l., 24.vi.2021, 1 ♀, LHI obs. + photo (DJP det.); Iași County, Iași – Rediu, Iazul Tăutești, 47°13'33.4"N, 27°28'06.7"E, 120 m a.s.l., 28.vii.2021, 3 ♀♀, at light, MJR leg., coll. PKG; Iași County, Iași – Miroslava, Valea lui David, 47°11'38"N, 27°28'2.114"E, ca. 90 m a.s.l., 9.vii.2021, 2 ♀♀, together with 1 ♂ of *Od.armiger*, LHI obs. + photo (DJP det.); Iași County, Bârnova Forest (ca. 47°00'37.4"N, 27°33'32.8"E), 4.vii.2005, 1 ♂, found accidentally on the ground during the day, LFI leg., coll. CUIR (for incomplete data on this record see Ruicănescu and Niţu 2008; Tatole et al. 2009; Stan and Niţu 2013); Iași County, Stânca near Comarna, 47°4'11.874"N, 27°48'13.403"E, 7.vii.2017, 1 ♂, at light, CMI.

**Banat**, Caraș-Severin County, Svatá Helena (Sfânta Elena) env., Kulhavá skála hill env., 44°42'11.47"N, 21°43'41.49"E, 357 m a.s.l., 1.vi.2012, 1 elytron on a path going through a pasture, BJN; Caraș-Severin County, Svatá Helena (Sfânta Elena), 44°40'29.8"N, 21°42'35"E, 325 m a.s.l., 18.vi.2017, 1 ♀ FSLG after sunset, ZCP, 44°40'57.73"N, 21°42'19"E, 350 m a.s.l., 23.vi.2017, 4 spec. FSLG after sunset, ZCP obs. (1 ♂ leg. et coll.); Caraș-Severin County, Mehadia, undated [19^th^ century], 1 ♀, “ex. coll. [Otto] Staudinger”, coll. MTDG; Mehedinți County, Tisové Údolí (Eibenthal), ca. 44°32'36.7"N, 22°10'20.4"E, ca. 420 m a.s.l., 28.v.2008, 1 ♂ flying slowly up to 0.5 m above a path crossing a forest-steppe meadow at 21.45 EEST (= 40 min after sunset), JKV.

**Muntenia**, “Bukarest” [= Bucharest], undated, 1 ♂, V[ladimír] Zoufal leg., coll. Vladimír Zoufal deposited in MMBC; Teleorman County, Poroschia, [ca. 40 m a.s.l.], no other data, 1 ♀ in coll. GANM; Buzău County, Măgura, Mănăstirea Ciolanu [= Ciolanu Monastery], 5.vii.2014, 1 ♂, at light (160 W mercury-vapor lamp), beech forest, VUB.

**Dobruja (Dobrogea)**, Tulcea County, Agighiol, 12.vi.1993, 1 ♀, Ioana Matache leg., coll. GANM; Tulcea County, Babadag [env.], [100–200 m a.s.l.], 20.vi.1958, 1 ♂, 20.vi.1968, 1 ♂, Nicolae Săvulescu leg., coll. GANM; 11.vii.1985, 1 ♂, at light (mercury-vapor lamp), foot of a forest-steppe loess hill, JHM leg., coll. VKS (for partial data on this record see Hillert et al. 2016); 16.v.2014, 2 ♀♀, Juhász leg., coll. GML; Tulcea County, Mănăstirea Codru [= Codru Monastery] env. (ca. 8 km S of Babadag), 44°48'55.47"N, 28°41'23.15"E, 110 m a.s.l., 6.vi.2016, 1 spec., IIB, 44°49'04.0"N, 28°40'57.9"E, 140 m a.s.l., 10.vi.2016, ca. 30 spec. FSLG after sunset, MVP obs. (1 ♂ leg., coll. NMPC); Constanța County, Băneasa – Canaraua Fetei, ca. 44°3'13.28"N, 27°40'15.07"E, ca. 115 m a.s.l., 17.vii.1965, 1 ♂, Nicolae Săvulescu leg., coll. GANM; Constanța County, Albești env., Hagieni Forest, ca. 50 m a.s.l., 20.vi.1964, 1 ♀, collector unknown, coll. GANM; Constanța County, Hagieni, ca. 50 m a.s.l., 18.vi.1995, 1 ♀, at light, CWP leg., coll. LKKA.

**Comment**

For Romania, which can be considered one of the countries at the centre of the species’ distribution, surprisingly small amounts of data have been published. New records from 22 Romanian localities are presented here.

### ﻿Moldova

**Published data**

**Călărași District (Raionul Călărași)**, Bularda near Dereneu, ca. 165 m a.s.l., 16.vi.1931, 3 ♂♂ and 4 ♀♀, Nicolai Zubowsky leg., coll. N. Zubowsky deposited in NMCM (Derjanschi et al. 2016; sex of the specimens specified by Valeriu Derjanschi pers. comm., 2021).

**Ialoveni District (Raionul Ialoveni)**, Dănceni, ca. 170 m a.s.l., 31.v.1929, 1 ♂, Nicolai Zubowsky leg., coll. N. Zubowsky deposited in NMCM (Derjanschi et al. 2016; sex of the specimen specified by Valeriu Derjanschi pers. comm., 2021).

**City of Chișinău (Municipiul Chișinău)**, Chișinău, [ca. 100 m a.s.l.], 20.v. and 10.vii.[between 1900–1915], no other data (Miller and Zubowsky 1917); 11.vii.1911, 1 ♀, Nicolai Zubowsky leg., coll. N. Zubowsky deposited in NMCM (Derjanschi et al. 2016; sex of the specimen specified by Valeriu Derjanschi pers. comm., 2021).

**Material examined**

**City of Chișinău (Municipiul Chișinău)**, Chișinău, 20.iv.1912, 1 ♀, Nicolai Zubowsky leg., Valeriu Derjanschi det., coll. Rodion Stepanov (box No. 10) deposited in NMCM.

**Anenii Noi District (Raionul Anenii Noi)**, Hîrbovăț env., Hîrbovăț Forest, ca. 285 m a.s.l., June 1970, 1 ♂, Rodion Stepanov leg., Valeriu Derjanschi det., coll. R. Stepanov (box No. 28) deposited in IZCM (for incomplete data on this record see Neculiseanu et al. 2002).

**Comment**

The first known record from Moldova (Chișinău) is mentioned by Miller and Zubowsky (1917). Old records from another two localities are reported by Derjanschi et al. (2016). The occurrence of the species in Moldova without further details is also mentioned by Panin (1957). This study presents the latest known Moldovan record from 1970.

### ﻿Ukraine

**Published data**

“Gubernia podolska” [= Podolian Governorate (Подольская губерния) of the Russian Empire, now Ukraine] (Hildt 1892).

“Volhynien” [= Volhynian Governorate (Волынская губерния), a historical region of the Russian Empire that included almost the entire area of today’s Volyn Oblast, as well as the Rivne and Zhytomyr Oblasts, northern parts of the Ternopil and Khmelnytskyi Oblasts, parts of the Podlaskie and Lublin Voivodeships of Poland and Brest Region of Belarus], undated, 2 spec., prof. Bresser leg. (Hochhuth 1873; Tenenbaum 1923; Savchenko 1931) – this record probably refers to data from Kremenets (Ternopil Oblast) reported by Eichwald (1830) – see below.

? **Ivano-Frankivsk Oblast (Івано-Франківська область)**, Chornohora (Чорногора) [mountain range], 9.viii.1939, 1 ♂, collector unknown, coll. SIZK (Vasko 2010) – the nature of the area (high mountains) does not correspond to the known requirements of the species and its occurrence here is unlikely; it is therefore probably a mislabelled specimen.

**Ternopil Oblast (Тернопільська область)**, Ternopil Raion (Тернопільський район), Zboriv (Зборів), 19.viii.1937, 1 ♀; collector unknown, coll. SIZK (Vasko 2010); Ternopil Raion (Тернопільський район), Ternopil (Тернопіль) env., “Gaje Tarnopolskie” [= Velyki Hai (Великі Гаї)], 26.vii.1884–1890, 1 spec., on a path, Michael Rybiński leg. et coll. (Rybiński 1897, 1903); Ternopil Raion (Тернопільський район), Ternopil (Тернопіль), no other data (Łomnicki 1913; Tenenbaum 1923; Savchenko 1938; Horion 1958); Ternopil Raion (Тернопільський район), “Zbaraż” [= Zbarazh (Збараж)] env., “Hnilice” [= Hnylytsi (Гнилиці)], no other data (Kuntze and Noskiewicz 1938); Kremenets Raion (Кременецький район), “Volhynia, Cremenezum” [= Kremenets (Кременець)] env., no other data (Eichwald 1830; Savchenko 1938).

**Chernivtsi Oblast (Чернівецька область)**, Bukovina (Буковина), Chernivtsi Raion (Чернівецький район), Chernivtsi (Чернівці), 4 spec. with no other data (Horion 1958), 2 ♂♂ and 3 ♀♀ with no oher data, coll. K. A. Penecke deposited in ZMNU (Vasko 2010), 1 ♀ in coll. NMPC (Hillert et al. 2016).

**Vinnytsia Oblast (Вінницька область)**, Vinnytsia Raion (Вінницький район), Vinnytsia (Вінниця) env., August 1928, 1 ♂, caught in flight in the evening, collector unknown (Savchenko 1933, 1938); Vinnytsia Raion (Вінницький район), Vinnytsia (Вінниця) env., Sabariv meadows (Сабарівські луки), no other data, 1 ♂ in coll. Yevhen Mykolaiovych Savchenko deposited in NHMU (Vasko 2010); Vinnytsia Raion (Вінницький район), Lypovets (Липовець), 24.vi.1926, 1 ♂, collector unknown, coll. Ye. M. Savchenko deposited in NHMU (Savchenko 1934, 1938; Vasko 2010); Vinnytsia Raion (Вінницький район), “Lintsi (Лінці)” [= Illintsi (Іллінці)], [ca. 215 m a.s.l.], 10.v.1905, 1 ♂ and 1 ♀, collector unknown (Savchenko 1934, 1938), 14.vi. and 19.vi. (year not specified), no other data (Savchenko 1938); Haisyn Raion (Гайсинський район), Trostianets-Podilskyi (Тростянець-Подільський) [= Trostianets (Тростянець)] env., “Zatishje” [= village of Obodivka (Ободівка)], 15.vii.1930, 1 ♀, caught in flight in the evening, V. Paliy leg., coll. Ye. M. Savchenko deposited in NHMU (Savchenko 1933, 1938; Vasko 2010).

**Odessa Oblast (Одеська область)**, Odessa Raion (Одеський район), Odessa (Одеса), 1827–1831, no other data (Krynicki 1832; Savchenko 1938; Trach and Gontarenko 2005), Odessa Raion (Одеський район), Odessa (Одеса) env., [1825–1860], 1 ♂, undated, [prof. I. B.] Bertoldi [leg.], “coll. University of Novorossiysk” (Kulikovskiy 1897; for information on Bertoldi’s collection see Sevastianov 2000, 2001), “2 spec. in coll. Gugel [or Hugel/Hügel]” (Kulikovskiy 1897); Odessa (Одеса) env., Bilhorod-Dnistrovskyi Raion (Білгород-Дністровський район), Sadove (Садове) env., Lymanskyi (Лиманський) nature reserve, ca. 46°15'19.9"N, 30°11'2.9", ca. 50 m a.s.l., 8.vi.2004, 2 ♀♀, at UV light, HDO (Gontarenko and Trach 2011; data specified by YSK pers. comm., 2021); Rozdilna Raion (Роздільнянський район), 4 km NW of Butsynivka (Буцинівка) village, 4.vi.2011, 1 ♀, at UV light, YKO leg., coll. VTO (Gontarenko and Trach 2011; sex specified by YSK pers. comm., 2021).

**Kyiv Oblast (Київська область)**, Kiyv (Київ), old town, May 1839, 4 spec. under a dead dog, June 1870, 1 spec. on a grassy path, Johann Heinrich Hochhuth leg. (Hochhuth 1873); Kyiv (Київ) env., no other data (Cherkunov 1889); Kyiv (Київ), undated, 1 spec., prof. Jelski leg., coll. of deceased J. Wańkowicz (Hildt 1896); Kyiv (Київ), Shevchenkivskyi Raion (Шевченківський район), Nyvky Park (Парк “Нивки”), 2.viii.1998, 1 ♂, dead on the ground, BVK leg., coll. SIZK (Vasko 2010); Kyiv (Київ), Holosiivskyi Raion (Голосіївський район), Holosiyiv Forest (Голосіївський ліс) [currently Holosiivskyi National Nature Park (Національний природний парк «Голосіївський»)], near the building of Astronomical Observatory of the National Academy of Sciences of Ukraine, 6.vi.1923, 1 ♀, Ye. M. Savchenko leg., coll. Ye. M. Savchenko deposited in NHMU (Savchenko 1934, 1938; Vasko 2010); Kyiv (Київ), Holosiivskyi Raion (Голосіївський район), Holosiyiv Forest (Голосіївський ліс) [currently Holosiivskyi National Nature Park (Національний природний парк «Голосіївський»), 10.vii.1928, 1 ♀, Ye. M. Savchenko leg., coll. Ye. M. Savchenko deposited in NHMU (Savchenko 1934, 1938; Vasko 2010); Kyiv (Київ), Holosiivskyi Raion (Голосіївський район), Holosiivskyi National Nature Park (Національний природний парк «Голосіївський»), no other data (Solomakha et al. 2020); Kyiv (Київ), Holosiivskyi Raion (Голосіївський район), Lysa Hora (Лиса гора), 20.vi.1998, 1 ♀, pitfall trap, H. Uspenskyi leg., coll. BVK; 19.vi.2007, 1 ♀, pitfall trap, RHK leg., coll. SIZK (Vasko 2010; data specified by RHK pers. comm., 2021); Obukhiv Raion (Обухівський район), Hryhorivka (Григорівка), 6.vi.1928, 1 ♂, collector unknown, coll. Ye. M. Savchenko deposited in NHMU (Savchenko 1938; Vasko, 2010); Obukhiv Raion (Обухівський район), Rzhyshchiv (Ржищів) env., area of the Ecological Research Centre “Hluboki Balyky (Глибокі балики)”, 49°57'44"N, 31°7'8"E, 5.–6.viii.2020, 1 spec., at light, VKK (Sheshurak et al 2020a); Bila Tserkva Raion Raion (Білоцерківський район), village of Luka (Лука), undated [probably between 1925–1939, Bohdan M. Vasko pers. comm., 2020], 1 ♀, Jenni leg., coll. Ye. M. Savchenko deposited in NHMU (Vasko 2010).

**Cherkasy Oblast (Черкаська область)**, Zvenyhorodka Raion (Звенигородський район), Talne (Тальне), 1 ♂ with no other data (Savchenko 1934, 1938); Cherkasy Raion (Черкаський район), Kaniv (Канів), hornbeam forest, 8.vi.1951, 1 ♀, collector not specified, coll. SIZK (Vasko, 2010); Cherkasy Raion (Черкаський район), Kaniv (Канів) env., Kaniv Nature Reserve (Канівський природний заповідник), no other data (Solomakha et al. 2020).

**Chernihiv Oblast (Чернігівська область)**, Novhorod-Siverskyi Raion (Новгород-Сіверський район), Novhorod-Siverskyi (Новгород-Сіверський) env., 51°59'N, 33°16'E, 18.vii.2003, 1 spec., I. V. Porokhniach leg., coll. GUNU (Vovk et al. 2005, 2016; Sheshurak et al. 2018, 2020b).

**Sumy Oblast (Сумська область)**, Shostka Raion (Шосткинський район), Matskove (Мацкове) env., ca. 51°28'48"N, 33°53'24"E, ca. 150 m a.s.l., 28.vii.2018, 1 spec., MZK (Kavurka et al. 2019).

**Poltava Oblast (Полтавська область)**, Lubny Raion (Лубенський район), Lubny (Лубни), [ca. 160 m a.s.l.], July (year and nummber of specimens not specified), Kruhlik [leg.], coll. Provincial Museum of Poltava (Ohloblin 1913; Savchenko 1938).

**Dnipropetrovsk Oblast (Дніпропетровська область)**, Synelnykove Raion (Синельниківський район), Raivka (Раївка), 1.viii.2000, 1 spec., A. M. Sumarokov leg. (Martynov 2003); Novomoskovsk Raion (Новомосковський район), Andriivka (Андріївка), 6.viii.1986, 1 spec., at light, A. M. Sumarokov leg. (Martynov 2003; Vasko and Bryhadyrenko 2011); Dnipro Raion (Дніпровський район), Dnipro (Дніпро) [Dnipropetrovsk until 19 May 2016], “около Днепропетровского Гослесхоза” [= near the Dnipropetrovsk State Forestry Enterprise, = Tunelna Balka (Тунельна балка) tract] (Vasko 2010; Vasko and Bryhadyrenko 2011; for detailed data see Material examined and new observations below); Dnipro Raion (Дніпровський район), Dnipro (Дніпро) [Dnipropetrovsk until 19 May 2016], 16.vi.2010, 1 ♀, collector not specified (Miessen 2011), 18.vi.2010, 1 ♀, Dementiev leg. (Brustel and Gouix 2012); Pavlohrad Raion (Павлоградський район), Kocherezhky (Кочережки) env., 21^st^ century, no other data (Vasko and Bryhadyrenko 2011).

**Material examined and new observations**

**Zakarpattia Oblast (Закарпатська область)**, Mukachevo Raion (Мукачівський район), “Schönb Ungarn” [= Hungary, Schenborn (Шенборн)], [ca. 190 m a.s.l.], “coll. Kirsch”, undated, 1 ♂, coll. MTDG.

**Ivano-Frankivsk Oblast (Івано-Франківська область)**, Kosiv Raion (Косівський район), Pistyn (Пістинь), [ca. 400 m a.s.l.], undated, 1 ♂,“A. St?kl” [the third letter is illegible] leg., coll. SMLU.

**Ternopil Oblast (Тернопільська область)**, Chortkiv Raion (Чортківський район), “Torskie, pow[iat] Zaleszcz[yki]” [= Zalishchyky (Заліщики) Powiat, Torske (Торське)], [ca. 250 m a.s.l.], 27.vi.[19]33, 1 ♂, collector unknown, coll. MIZP; Dniester Canyon National Nature Park (Національний природний парк «Дністровський каньйон»), Chortkiv Raion (Чортківський район), Horodok (Городок), 48°38'18.96"N, 25°50'11.04"E, ca. 140 m a.s.l., 6.vii.2018, 6 ♂♂ and 2 ♀♀ FSLG after sunset, steppe meadow on the terrace of the Dniester (Дністер) river, YKL, YHS and ABZ leg. coll. YKL and YHS (for photographs of the site see Fig. 13C, D).

**Chernivtsi Oblast (Чернівецька область)**, Bukovina (Буковина), Chernivtsi Raion (Чернівецький район), “Czernowitz” [= Chernivtsi (Чернівці)], no other data, 1 ♂ in coll. Georg Frey deposited in NHMB, 1 ♂ and 2 ♀♀ (ex original coll. Josef Breit, Vienna) in coll. Georg Frey deposited in NHMB, 2 ♂♂ and 3 ♀♀ in coll. ZMNU, 1 ♂ and 2 ♀♀ in coll. UMJG.

**Vinnytsia Oblast (Вінницька область)**, “Киевская г[уберния], Сквирский у[езд]” [= Kiev Governorate of the Russian Empire (disestablished 1925), Skvirsky Uyezd (incorrectly, it was actually Lipovetsky Uyezd), currently Vinnytsia Raion (Вінницький район)], “Ильинцы” [= Illintsi (Іллінці)], 14.vi.[year not specified], [ca. 215 m a.s.l.], 1 ♀, collector not specified, coll. ZINR (probably one of the two specimens mentioned by Savchenko (1938) – see published data).

**Odessa Oblast (Одеська область)**, Bilhorod-Dnistrovskyi Raion (Білгород-Дністровський район), Karolino-Buhaz (Кароліно-Бугаз), Studentska (Студентська) railway station, ca. 46°9'56.34"N, 30°33'24.58"E, 22 m a.s.l., 15.vi.2017, 1 ♂ crawling on the ground, OKO.

**Kyiv Oblast (Київська область)**, Bila Tserkva Raion (Білоцерківський район), “Hałajki, Kijow[ska] g[ubernia]” [= Kiev Governorate, Halaiky (Галайки)], [ca. 190 m a.s.l.], [probably 19^th^ Century], no other data, 1 ♂ in coll. MIZP; Bucha Raion (Бучанський район), Muzychi (Музичі), ca. 160 m a.s.l., 18.vii.2006, 1 ♂, at light, M. Nesterov leg., coll. SIZK; “Kiew” [= Kyiv (Київ)], undated, 1 ♀ in Hartmann [leg.], coll. NMPC; “Kieff” [= Kyiv (Київ)], May [19]05, 1 ♂, Shelushko [leg.], coll. ZINR; Fastiv Raion (Фастівський район), Novosilky (Новосілки), ca. 180 m a.s.l., 21.vii.2012, 1 ♀, M. Nesterov leg., coll. SIZK; Obukhiv Raion (Обухівський район), Mali Dmytrovychi (Малі Дмитровичі), 50°12'59"N, 30°32'29"E, ca. 160 m a.s.l., 17.vii.2010, 1 ♂, at light, together with 1 ♂ of *Od.armiger*, VSK leg., coll. KLP; 29.v.2014, 1 ♂ and 1 ♀, at light, RHK; 25.v.2016, 1 ♂, 28.v.2016, 1 ♀, 13.vi.2020, 1 ♂ and 2 ♀♀, at light, STK; Obukhiv Raion (Обухівський район), Rzhyshchiv (Ржищів), Taras Shevchenko Park (Парк імені Тараса Шевченка), 49°57'58.1"N, 31°02'39.5"E, 112 m a.s.l., 18.ix.2021, 1 ♀ crawling on the ground at 16:22 EEST, HTR obs. + photo (DJP det.); Obukhiv Raion (Обухівський район), Rzhyshchiv (Ржищів) env., area of the Ecological Research Centre “Hlyboki Balyky (Глибокі балики)”, 49°57'44.082"N, 31°7'8.094"E, ca. 150 m a.s.l., 18.vi.2021, 1 ♀, at light OVK obs. + photo + recorded an audio track of its stridulation (DJP det.); 49°57'43.729"N, 31°7'9.782"E, 19.vi.2021, 1 ♂, together with 1 ♂ and 2 ♀♀ of *Od.armiger*, OVK obs. + photo (DJP det.); Myronivka Raion (Миронівський район), Tulyntsi (Тулинці), ca. 150 m a.s.l., 9.vi.2020, 1 ♀, at light, STK; Myronivka Raion (Миронівський район), Velykyi Bukryn (Великий Букрин) env., 49°57'13"N, 31°18'8"E, 155 m a.s.l., 27.vi.2009, 1 ♀, at light, VSK.

**Cherkasy Oblast (Черкаська область)**, Cherkasy Raion (Черкаський район), Kaniv (Канів) env., Kaniv Nature Reserve (Канівський природний заповідник), 49°43'12"N, 31°31'19"E, ca. 200 m a.s.l., 20.vi.1984, 6 spec. excavated from their burrows, steppe slope in a hornbeam forest, KVM and VGG leg., coll. MKY and MPGU.

**Kirovohrad Oblast (Кіровоградська область)**, Oleksandriia Raion (Олександрійський район), Semyhiria (Семигір’я) env., 49°0'29.52"N, 32°54'21.24"E, 135 m a.s.l., 2.vii.2020, 1 ♂, at light, DPS obs. + photo (Fig. 14D).

**Figure 15.**
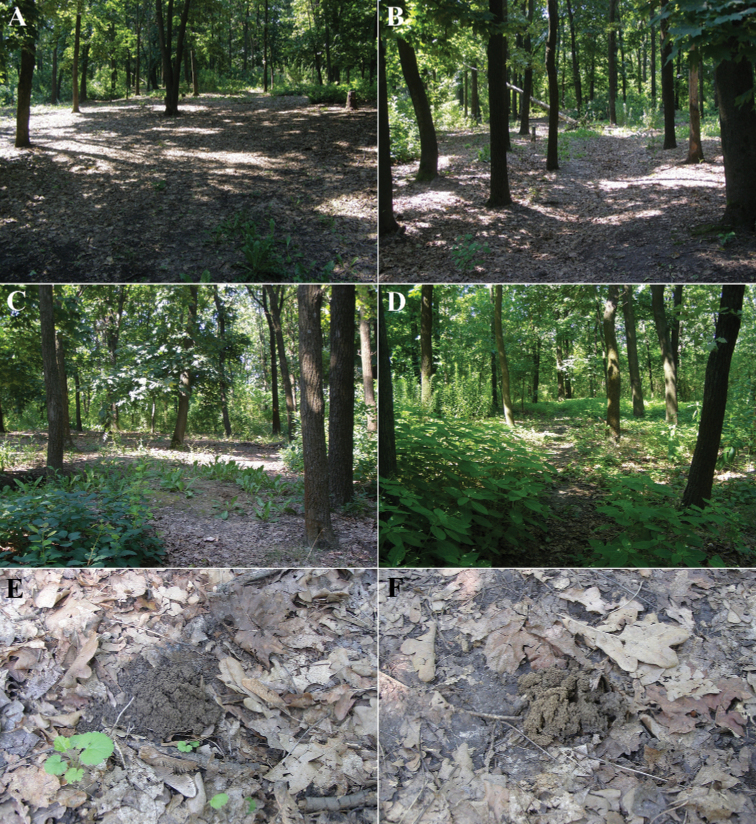
Tunelna Balka tract (Ukraine), locality with abundant occurrence of *B.unicornis***A–D** views of the site (photographs by Oleksandr O. Sukhenko) **E, F** burrows dug by adults of *B.unicornis* with push-ups (photographs by Oleksandr O. Sukhenko).

**Dnipropetrovsk Oblast (Дніпропетровська область)**, Dnipro Raion (Дніпровський район), Dnipro (Дніпро) [Dnipropetrovsk until 19 May 2016], Tunelna Balka tract (Тунельна балка) [the name of an area with oak forest in the sou­thern part of the city, see Fig. 15], 48°25'11.8"N, 35°02'59.8"E, 15.vii.2005, 1 ♂ and 1 ♀ excavated from their burrows near the edge of a forest path under oak tree, OSD leg., coll. OSD and SIZK; 16.vi.2006, 3 ♂♂ and 4 ♀♀ excavated from their burrows, OSD leg., coll.SIZK; 16.vii.2006, 1 ♂ and 1 ♀ excavated from their burrows, OSD; 26.vii.2006, 1 ♂ excavated from its burrow near the edge of a forest path under oak tree, OSD; 20.–25.vii.2007, 1 ♂, OSD leg., coll. SIZK; 16.–17.vi.2008, 4 ♂♂ and 5 ♀♀ excavated from their burrows + at light, OSD leg., coll. SIZK; 18.vi.2008, 1 ♀, at light, OSD leg., coll. SIZK; 1.–10.vii.2008, 3 ♂♂ and 8 ♀♀ excavated from their burrows + at light, OSD leg., coll. SIZK (for partial data on these records see Vasko 2010); 3.vi.2013, 1 ♂ and 1 ♀ excavated from their burrows, OSD leg., coll. GML; 48°25'10.7"N, 35°02'54.5"E, June 2009, 3 ♀ excavated from its burrow near the edge of a forest path under oak tree, OSD leg., coll. OSD and GML; 16.vi.2010, 1 ♀, 8.vi.2010, 1 ♂, 18.vi.2010, 1 ♂ and 1 ♀, at light, OSD leg., coll. SIZK; 48°25'02.8"N, 35°02'26.6"E, 5.vii.2014, 1 ♂, at light, OSD leg., coll. SIZK; 8.–15.vi.2014, 13 spec. excavated from their burrows near the edge of a forest path under oak trees, OSD (1 ♀ in coll. DJP); 48°25'04.8"N, 35°02'40.6"E, 6.vi.2015, 1 ♂ excavated from its burrow under oak tree, OSD leg., coll. DJP; 16.vii.2015, 1 ♂, OSD leg., coll. NHMK; 48°25'12.8"N, 35°03'00.7"E, 8.vi.2010, 1 ♂ in flight, OSD; 18.vi.2010, 1 ♂ and 1 ♀, at light, OSD; 11.vi.2015, 1 ♀, at light at 21.40 EEST (= 61 min after sunset), OSD leg., coll. DKP deposited in NMPC; 48°24'57.3"N, 35°02'33.1"E, 1.vii.2015, 1 ♂ and 1 ♀ excavated from their burrows under oak tree (distance between these two burrows was 40 cm), OSD leg., coll. DJP; 48°25'00.0"N, 35°02'22.4"E, 7.vii.2015, 1 ♂, dead on a forest path, OSD leg., coll. DJP; 48°25'01.1"N, 35°02'23.6"E, 7.vii.2015, 1 ♀ excavated from its burrow under oak tree, OSD leg., coll. DJP; 48°24'57.1"N, 35°02'13.0"E, 9.–12.vii.2015, 1 ♂ and 1 ♀, pitfall traps, OSD leg., coll. DJP and DKP (deposited in NMPC); 48°25'02.1"N, 35°02'22.6"E, 8.vi.2016, 4 ♂♂ and 3 ♀♀ excavated from their burrows under oak trees, OSD leg., coll. DJP and GML; 48°25'0.70"N, 35° 2'22.30"E, 106 m a.s.l., 10.vi.2016, more spec. excavated from their burrows, OSD leg., 5 ♂♂ and 2 ♀♀ in coll. ASK, 1 ♂ and 2 ♀♀ in coll. VSM, 1 ♀ in coll. YSK; 48°24'55.9"N, 35°02'34.7"E, 16.–17.vi.2016, 2 ♂♂ excavated from their burrows under oak trees, together with 2 ♂♂ of *Od.armiger*, OSD leg., coll. DJP; 48°24'55.50"N, 35°2'33.20"E, 111 m a.s.l., 13.–25.vi.2020, 9 ♂♂ and 4 ♀♀, OSD leg., coll. ASK; 48°24'57.6"N, 35°02'29.4"E, 100 m a.s.l., 14.vii.2021, 1 ♂ excavated from its burrow, OSD leg., coll. DJP; 48°24'57.4"N, 35°02'30.7"E, 100 m a.s.l., 14.vii.2021, 2 ♂♂ excavated from its burrow, OSD leg., coll. DJP and GML; Dnipro Raion (Дніпровський район), “Opytnoye” (“Опытное”) [= Doslidne (Дослідне)], research area of the Institute of Grain Crops of NAAS of Ukraine (Інститут зернових культур НААН України), 48°22'58.2"N, 35°02'01.7"E, 143 m a.s.l., June 1978, remains of a dead specimen (elytra) on the ground near the greenhouse, OSD leg. et coll. (for partial data on this record see Vasko 2010; Vasko and Bryhadyrenko 2011).

**Comment**

In the checklist of Ukraine (Martynov 2012), the species is listed from 12 of the 25 oblasts. The critical revision performed in the present study confirms occurrence in ele­ven of them with two additional ones: Sumy and Kirovohrad oblasts. Records from the Right Bank Ukraine are summarised by Vasko (2010). In the present study, new records from 15 Ukrainian localities are given, two of them (Schenborn and Semyhiria) being the first published records for the Zakarpattia and Kirovohrad oblasts, respectively.

### ﻿Bulgaria

**Published data**

**Vratsa Province (Област Враца)**, Oryahovo (Оряхово), no other data (Kovachev 1905).

**Ruse Province (Област Русе)**, Vetovo (Ветово), no other data (Kovachev 1905).

**Razgrad Province (Област Разград)**, Razgrad (Разград) env., 16.v.1905, in the barracks area, number of specimens not specified, Andrey Markovich leg, 13.vi.1907, in the vineyards, number of specimens not specified, Andrey Markovich leg. (Markovich 1909; Guéorguiev and Bunalski 2004).

**Pleven Province (Област Плевен)**, Pleven (Плевен), April [year and collector not specified] (Nedyalkov 1909; Mikšić 1959; Guéorguiev and Bunalski 2004).

**Sofia Province (Софийска област)**, Gorna Malina (Горна Малина) – “ДЗС” [= Държавно земеделско стопанство, area of the State Farm], ca. 650 m a.s.l., 7.vii.1969, 2 ♂♂ and 1 ♀ excavated from the soil from a depth of ca. 10 cm on a pasture (northern slope), collector not specified (Zaharieva-Stoilova 1974); Lozen Mountain (Лозенска планина), 5 km SE of German (Герман), “Germanski m.” [= German Monastery of St John of Rila (“Св. Иван Рилски”)], 31.v.[1]915, 1 ♂, Dr Iw[an Jossifow] Buresch [leg.], coll. NMSB (Guéorguiev and Bunalski 2004; data supplemented by Borislav Guéorguiev pers. comm., 2022; sex of the specimen corrected by the photograph).

**Shumen Province (Област Шумен)**, Shumen (Шумен), ca. 200 m a.s.l., 1914, 2 ♂♂, Hanuš leg., coll. MYP (Král and Malý 1993).

**Burgas Province (Област Бургас)**, “Michurin (Мичурин)” [= Tsarevo (Царево)], 29.–30.vi.1982, 1 ♂, at light., BSP (Král and Malý 1993).

**Silistra Province (Област Силистра)**, Dulovo (Дулово) env., Karakuz forest (гора Каракуз) [note: the locality label states “Gora Kanagöl, Dulowsko”], 14.vi.1952, 1 ♀, P[encho Stefanov] Drenski leg., coll. NMSB (Guéorguiev and Bunalski 2004; data specified by Borislav Guéorguiev, 2022).

**Material examined ans new observations**

**Vidin Province (Област Видин)**, Dimovo (Димово) env., steppe meadow near the Archar (Арчар) river, 43°45'28.7"N, 22°44'51.1"E, 110 m a.s.l., 26.vi.2010, 3 ♂♂ and 2 ♀♀ FSLG after sunset just before a storm, together with several spec. of *Och.chrysomeloides*, no wind, 26 °C, ASH (Fig. 10C, D).

**Varna Province (Област Варна)**, Oreshak (Орешак) env., 43°17'50.67"N, 27°53'47.29"E, 300 m a.s.l., 6.vii.2020, 1 ♀ flying up to 0.5 m above the grass at ca. 22.00 EEST, forest-steppe clearing in an oak forest, MTM obs. + photo (Fig. 10E, F).

**Comment**

So far, only nine localities have been published for Bulgaria. This study presents new records from two additional sites.

### ﻿Turkey (European part)

**Published data**

**Marmara Region (Marmara Bölgesi)**, Edirne Province, ca. 15 km E of Edirne [according to Walter Heinz, pers. comm., these were periodically flooded meadows on the banks of the Ebros River S of Edirne], 27.iii.[19]88, 1 ♀, WHS leg., coll. DKP deposited in NMPC (Hillert et al. 2016).

### ﻿Turkey (Asian part)

**Published data**

**Figure 16.**
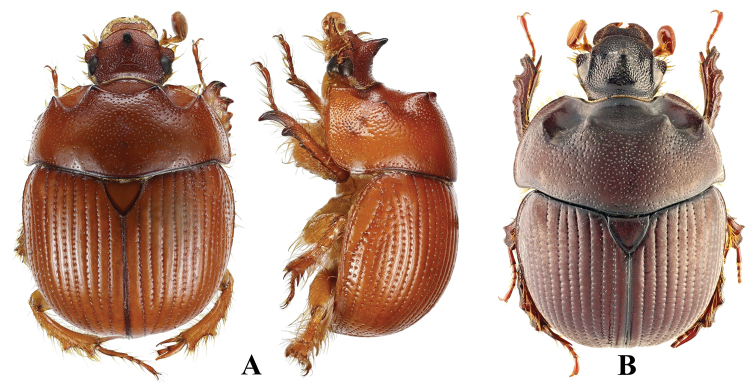
The only two specimens of *B.unicornis* so far known from Asia **A** Turkey, Denizli env., [Çürüksu River valley], “Goundely” [= Goncalı] [railway station env.], May [19]26, [Hans] Kulzer leg., coll. ZSMG, dorsal and lateral views, body length 12.0 mm (photographs by Michael Balke) **B** Turkey, [Büyük Menderes River valley], “Bereketli (Denizli)” [= Bereketli near Nazilli], 5.vii.1965, [Helio] Pierotti & [Antonello] Perissinotto leg., coll. MSNG, body length 12.5 mm (photograph by Marcello Romano).

**Aegean Region (Ege Bölgesi)**, Denizli Province, Denizli env., [Çürüksu River valley], “Goundely” [= Goncalı] [railway station env.], ca. 200 m a.s.l., May [19]26, 1 ♂, [Hans] Kulzer leg., coll. ZSMG (Hillert et al. 2016; locality identified by the author, year corrected by Oliver Hillert pers. comm., 2021) – see Fig. 16A.

**Material examined**

**Aegean Region (Ege Bölgesi)**, Aydın Province, [Büyük Menderes River valley], “Bereketli (Denizli)” [= Bereketli near Nazilli], ca. 80 m a.s.l., 5.vii.1965, 1 ♂, [Helio] Pierotti and [Antonello] Perissinotto leg., DJP det. (2021), coll. Helio Pierotti deposited in MSNG – this record was published under a misidentification as *Bolbelasmustauricus* Petrovitz, 1973 (Arnone and Massa 2010) – see Fig. 16B.

**Comment**

Only the three records mentioned above are known for Turkey. A record from Osmaniye Province (Kadirli) reported by Lodos et al. (1999) most likely refers to the related species *Bolbelasmusnireus* (Reitter, 1895) (see Miessen 2011; Hillert et al. 2016; Sommer et al. 2021).

### ﻿Dubious faunistic records

**Great Britain**

**Published data**

**East of England**, Cambridgeshire, marshes between Peterborough and Wisbech, beginning of summer 1807, 1 ♂ and 1 ♀, plant materials alluviated by flooded River Nene, together with 2 ♂♂ and 3 ♀♀ of *Od.armiger*, William Skrimshire leg. (Skrimshire 1812; Curtis 1829a, b; Stephens 1829, 1830, 1839).

**Comment**

Skrimshire’s record was probably adopted by several subsequent authors (e.g., Mulsant and Rey 1871; Sajó 1910b; Boucomont 1912; Paulian 1941; Tesař 1957; Neculiseanu et al. 2002; Trnka 2009; Vasko 2009; Arnone and Massa 2010; Vasko and Bryhadyrenko 2011; Vidlička 2011). According to Darren Mann (pers. comm. 2021), it is based on a misidentified *Od.armiger*, with no material from the British Isles. Also, Paulian and Baraud (1982) considered the report from England to be erroneous without giving any explanation. *Bolbelasmusunicornis* was no longer listed for Great Britain by the following authors: Fowler (1890), Joy (1932), Britton (1956), Jessop (1986), Mann (2012), and Lane and Mann (2016). Even in both editions of the Catalogue of Palaearctic Coleoptera (Král et al. 2006; Nikolajev et al. 2016), the United Kingdom is not listed as a country of occurrence of this species.

**Country not specified [probably Russia**]

**Material examined**

**Figure 17.**
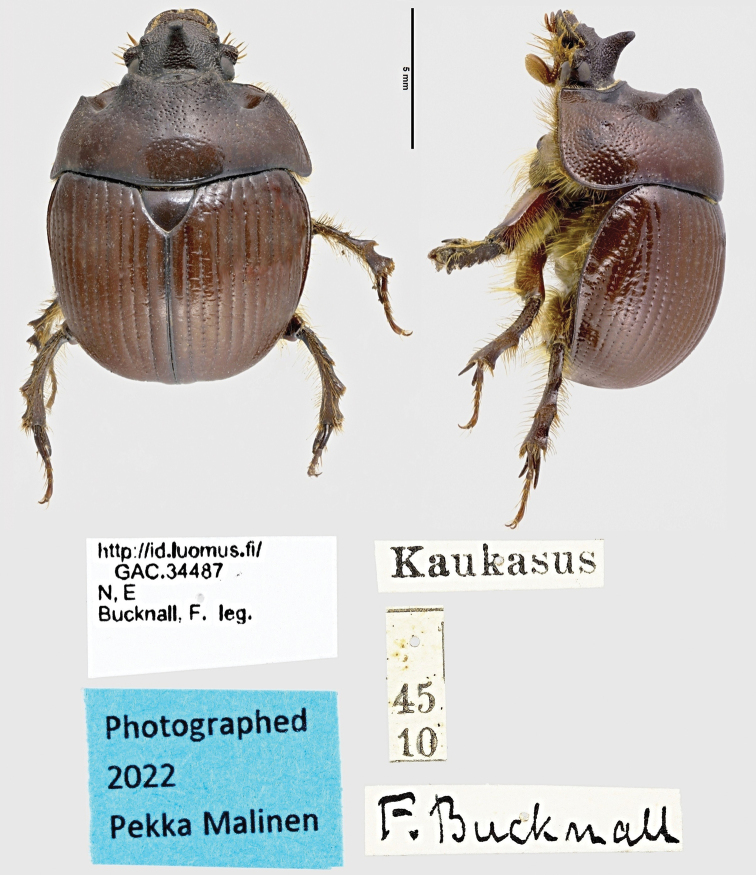
The only specimen of *B.unicornis* that could originate from the Caucasus region, deposited in FMNH (photographs by Pekka Malinen, edited by Peter Kurina).

“Kaukasus” [= Caucasus], locality and date not specified, 1 ♂, F. Bucknall [leg.], coll. FMNH (Fig. 17).

**Comment**

This specimen represents the first record of the species from the Caucasus region. Unfortunately, country is not specified (it could be Russian part of the area, but confusion of the locality label cannot be discounted). Shokhin (2007) did not record the species from Southern Russia.

## ﻿Remarks on distribution

**Figure 18.**
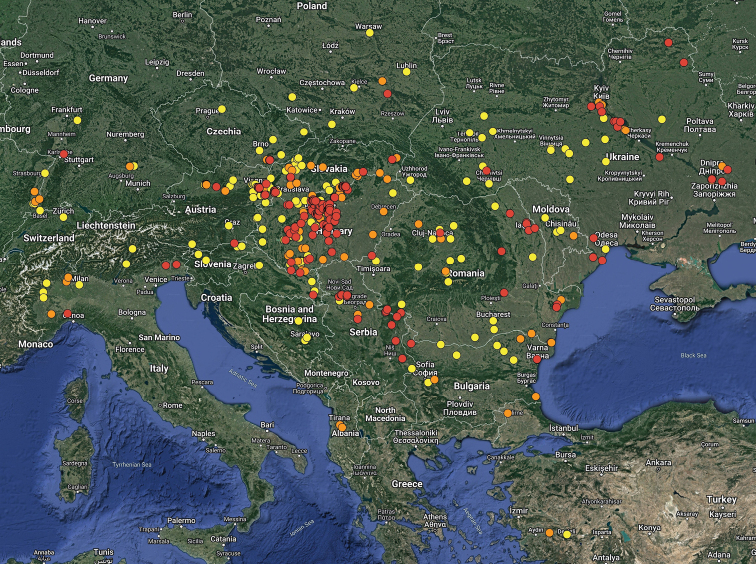
Distribution of B.unicornis (yellow circles – records before 1950, orange circles – records between 1950–1999, red circles – records after 1999).

*Bolbelasmusunicornis* was included in checklists, catalogues, and monographs dealing with the scarabaeoid fauna of several countries as follows: France (Montreuil 2014), Germany (Köhler and Klausnitzer 1998; Bleich et al. 2022), Italy (Ballerio et al. 2014; Carpaneto et al. 2021), Poland (Burakowski et al. 1983), Czech Republic and Slovakia (Juřena and Týr 2008; Zahradník 2017), Austria (Jäch et al. 1994), Hungary (Ádám 1994), Slovenia (Brelih et al. 2010), Bosnia and Herzegovina (Lelo 2006), former Yugoslavia (Mikšić 1970), Albania (Murraj 1962), Romania (Chimişliu 2004), Republic of Moldova (Bacal et al. 2013), Ukraine (Martynov 2012), Bulgaria (Bunalski 2001), and Turkey (Carpaneto et al. 2000). Also, it was mentioned in two editions of the Catalogue of Palearctic Coleoptera (Král et al. 2006; Nikolajev et al. 2016). For the general distribution of the species see Fig. 18.

No records are known from mainland Greece. Records from the Greek island of Crete (Heyden 1884; Oertzen 1886; Mikšić 1959; Neculiseanu et al. 2002; Trnka 2009) refer to species later described as *Bolbelasmuskeithi* Miessen and Trichas 2011 (see Miessen and Trichas 2011; Hillert et al. 2016), and the records from Rhodes (Schatzmayr 1936; Paulian 1941, 1959; Tesař 1957; Mikšić 1959; Petrovitz 1959; Paulian and Baraud 1982; Petersen et al. 2006) most likely relate to *B.nireus* (see Sommer et al. 2021). The record from Greece reported by Reitter (1892) probably refers to Crete, and thus to *B.keithi*. Also, all subsequent records from Greece (Paulian 1941, 1959; Krikken 1977; Lumaret 1990; Neculiseanu et al. 2002; Szwałko 2004; Agoglitta et al. 2006; Král 2006; Král et al. 2006; Vasko 2009; Vasko and Bryhadyrenko 2011; Vidlička 2011; Brustel and Gouix 2012; Alonso-Zarazaga et al. 2013; Gutowski and Przewoźny 2013; Trizzino et al. 2013; Potocký and Majzlan 2015; Nikolajev et al. 2016; Ćurčić et al. 2019; Schoolmeesters 2019; Nuß and Jäger 2020) most likely refer to the Greek islands and thus to *B.keithi* or *B.nireus*.

Records from Cyprus (Keith 2002; Král 2006; Alonso-Zarazaga et al. 2013; Potocký and Majzlan 2015; Schoolmeesters 2019; Nuß and Jäger 2020) refer to species described as *Bolbelasmusmakrisi* Miessen, 2011 (see Miessen 2011; Hillert et al. 2016; Sommer et al. 2021).

All the records from the Soviet Union and Russia (e.g., Medvedev 1965; Baraud 1992; Agoglitta et al. 2006; Král et al. 2006; Brelih et al. 2010; Brustel and Gouix 2012; Ballerio et al. 2014; Merkl 2014; Hillert et al 2016; Ćurčić et al. 2019; Schoolmeesters 2019) apply to Ukraine (Andrey V. Frolov and Liliya A. Akhmetova pers. comm., 2020). In the second edition of the Catalogue of Palaearctic Coleoptera (Nikolajev et al. 2016), Russia is no longer listed as a country of occurrence of this species.

The species has also been listed for Belarus, Montenegro, and the Republic of North Macedonia (Chobot and Mourek 2008; Alonso-Zarazaga et al. 2013; Potocký and Maj­zlan 2015; Nuß and Jäger 2020), but there are no exact data from these countries, although its occurrence at least in Montenegro and North Macedonia is highly probable. Other countries where the species is highly likely to occur are Kosovo, mainland Greece (especially Macedonia and Thrace), and western Russia (e.g., Bryansk, Kursk, Belgorod and Rostov oblasts, and Krasnodar Krai). Occurrence in southern Belarus cannot be ruled out either. The species is most likely extinct in France, Swi­tzerland, Poland, and the Czech Republic (considering the lack of suitable habitats and any new records).

Figure 18 probably does not reflect the real distribution of *B.unicornis* because of insufficient surveys in some countries. In countries such as Serbia, Romania, Moldova, Ukraine, and Bulgaria, there are probably many localities with *B.unicornis* that have not yet been discovered due to low collecting activity and the lack of application of effective collecting methods for this beetle species (see Monitoring methods below).

The northernmost known historical locality of *B.unicornis* is Warsaw (Poland), while the northernmost locality with a recent record is Novhorod Siverskyi (northern Ukraine). The southernmost historical locality is Denizli (southwestern Turkey), while the southernmost recent localities are Babin Kal (Serbia) and Oreshak (Bulgaria). The westernmost historical locality is Mulhouse (Alsace, France), while the westernmost recent localities are Bruchsal (Baden, Germany) and Lerma (Piedmont, Italy). The eas­ternmost locality with a recent record of the species is Kocherezhky (Ukraine), which is also the easternmost known point of occurrence of the species.

## ﻿Natural history of Bolboceratinae

### ﻿Evening flights of *B.unicornis*

Adults of *B.unicornis* spend most of their time underground. Above-ground activity is limited to short flight periods after sunset. Exceptionally, adults have been observed crawling on the ground during daylight hours (see Faunistic records). Flight statistics from each site are shown in Tables 1–8. A total of 63 periods of flights was documented at eight localities. The flights occurred in the date range from 27 May to 9 September with a total of 884 flying individuals observed. Both males and females flew, with slightly fewer females (ca. 44%). By comparison, in the congener *B.gallicus*, only 5% of the 830 individuals found were females (Rahola Fabra 2004) but in that study, these were mostly beetles excavated from their burrows. The flights of adults of *B.unicornis* started, on average, 35 minutes after sunset and terminated 60 minutes after sunset. For the start of flights, the minimum limit recorded was 23 minutes after sunset, and the maximum limit was 52 minutes after sunset. For the end of flights, the minimum and maximum limits were 35 and 86 minutes after sunset, respectively. On one occasion, a large number of specimens were observed flying around midnight (Josef Pavlas pers. obs., see Faunistic records). The average duration of flights was of 25 minutes, with the minimum and maximum limits of 4 and 63 minutes, respectively. The ave­rage air temperature during flights was 21 °C with limits of 14 and 26 °C. However, it is likely that the beetles are able to fly at lower temperatures, as has been observed, for example, in the Australian bolboceratine *Blackburniuminsigne* (Lea, 1916), adults of which have been found flying to lights at 4–6 °C (Howden et al. 2007). Flights of *B.unicornis* occurred exclusively after heavy rains when the soil was moist to a depth of at least ca. 30 cm. The flights were also affected by the wind intensity. Most of the flying adults were observed when there was no wind, whereas flights did not occur at all when the wind was strong. Light rain or heavy fog had no effect on the flying beetles, and, in one case, the beetles were found flying even with moderate rain (Filip Štrba pers. comm.). Similarly, flights of adults of *Odonteusarmiger* were observed during rain (Ivo Jeniš and Ilja Trojan pers. comm.). Adults of *B.unicornis* usually fly very slowly at a height of 20–50 cm above the ground, sometimes literally hovering in the same spot. However, in windy conditions they have been observed to fly faster and also at greater heights, ca. 1–2 m above the ground. Individuals flying quickly around a pile of logs at the edge of a forest were observed by the author near the village of Hajnáčka in southern Slovakia (see Faunistic records). This phenomenon was also observed in *Od.armiger* (Ilja Trojan and Ivo Jeniš pers. comm.): adults of this species were flying around the fallen oak trunk and piles of wet logs after sunset. When disturbed, the flying specimens of *B.unicornis* either immediately fell into the grass and buried themselves or accelerated their flight, increasing the height from the ground and flying away. This also applies to disturbances caused by too strong light source, e.g., from a headlamp. During flights, most beetles show light-aversion and avoid light sources; the individuals that were attracted to light were single cases only. These were mostly long-distance flights that occurred later in the night. Very rarely a few individuals did fly to the illuminated canvas just after sunset when it was not yet completely dark (Tamás Kiss, Ondřej Sabol, Tibor Spevár obs., see Faunistic records). In most Czech and Slovak localities, adults of *B.unicornis* were flying together with *Od.armiger* and *Och.chrysomeloides* or *O.integriceps* (see Faunistic records and Juřena et al. 2008). Apparently, these species almost always occur together at the sites (see also below).

### ﻿Feeding and nesting behaviour of bolboceratines

More than 50 individuals of *B.unicornis* were excavated from their burrows from depths of 5–60 cm during daylight hours. The length and the shape of the burrows varied, with the male burrows often changing direction from vertical to horizontal, whereas the female burrows usually descended vertically, changing direction only slightly and often leading to depths greater than those of males. In the dry periods and at the end of the season, the beetles burrow to the depths of more than 50 cm (e.g., Tomáš Vendl obs., see Faunistic records). When excavating adults, sometimes two to three individuals were found in a single burrow, even of the same sex (e.g., two males; see Faunistic records and Juřena et al. 2008). Similar observations were made, for example, by Mollandin de Boissy (1906) in the congeneric species *B.gallicus*, and by Robert J. Sim in some American *Odonteus* species (Wallis 1928). In contrast, Manee (1908) excavated ca. 100 specimens of the North American bolboceratine *Bradycinetulusferrugineus* (Palisot de Beauvois, 1809) in North Carolina, but he never found specimens of the same sex in the same burrow. On several occasions, individuals of *B.unicornis*, *Od.armiger*, and *Och.chrysomeloides* (or *O.integriceps*) have been found together in a single burrow (see Faunistic records and Juřena et al. 2008). Similarly, Robert J. Sim found representatives of three different genera *Bolbocerosoma*, *Eucanthus*, and *Odonteus* together in a single burrow (see Wallis 1928).

As for the feeding habits of *B.unicornis*, in none of the observations made by the author was the burrow found to lead to the sporocarp of hypogeous fungus or to the mycorrhizal roots of a shrub or tree. Nothing that could be considered as their food was ever found close to the buried individuals. In contrast, the Hungarian researchers repeatedly excavated the beetles near Budapest from the immediate vicinity of sporocarps of the large-spored pea truffle *Glomusmacrocarpum*, which were approximately the size of a fingernail, together with more specimens of *Od.armiger* (Bratek et al. 1992; Merkl 2003, 2014, 2015; Nádai 2006; Merkl and Vig 2009). In addition, according to Ottó Merkl (pers. comm.), an adult of *B.unicornis* was found on a sporocarp of *Tuber* sp. in the Baranya County in southwestern Hungary (see also Merkl 2014), and Ćurčić et al. (2019) reported that one specimen was excavated under a hazel shrub (*Corylusavellana*) together with sporocarps of *Tuber* sp. in the Belgrade District of Serbia. These findings support earlier hypotheses about the mycetophagy of the species (cf. e.g., Sajó 1910a, b; Ohaus 1929; Roubal 1936; Koch 1989). For *B.gallicus*, Rahola Fabra (2004) reported that the burrows of beetles often led to dead roots in various stages of decomposition, but never to the sporocarps of hypogeous fungi. This is in contrast to the observations by Fabre (1900, 1907, 1920), who found adults of *B.gallicus* on the sporocarps of *Hydnocystisarenaria* and *Tuberrequienii*, and Béguin (1906), who reported finding adults on sporocarps of *Tuberaestivum*. According to Rahola Fabra (2004), even in 20 years of field observations, the natural food of *B.gallicus* could not be determined with certainty. That author reported that in captivity, adults ingested sporocarps of *Tubermelanosporum*, *Rhizopogon* sp. and *Peziza* sp., but he did not consider this as unequivocal evidence of obligate mycetophagy by the species. According to Rahola Fabra (2004), dissection studies showed that the gut of adults of *B.gallicus* contained unspecified organic matter in 60% of individuals captured in the wild. Sim (1930) found that adults of the American bolboceratine *Odonteusdarlingtoni* (Wallis, 1928) stored a mass of sporocarps of ectomycorrhizal basidiomycete *Rhizopogonpachyphloes* in their burrows, and Howden (1955) reported adults of this species feeding on sporocarps of *Rh.nigrescens*. In the European species *Od.armiger*, Miquel and Vasko (2014) reported finding one adult feeding on a large sporocarp of *Rh.luteolus*, partially decayed, together with two individuals of *Anoplotrupesstercorosus* (Hartmann in Scriba, 1791), and another individual feeding on a sporocarp of *Glomusmicrocarpum*. Furthermore, these authors reported that near burrows dug by adults of *Od.armiger* kept in captivity, sporocarps of *Endogonelactiflua* were found. In contrast, adults of the genus *Eucanthus*, for example, probably do not ingest any food at all (Howden and Cooper 1977). According to Howden (2003), even adults of the genus *Bolbocerosoma* do not feed, but this is contradicted by new findings by Japanese researchers, who found that adults of *Bolbocerosomanigroplagiatum* (C. O. Waterhouse, 1875) feed on sporocarps of arbuscular mycorrhizal fungi (Higurashi and Tanahashi 2014; Aratani 2017; Higurashi et al. 2019). Higurashi and Tanahashi (2014) reported that bits of sporocarps of *Glomus* sp. were carried to the surface of the soil by adults of *B.nigroplagiatum*, then moved to another place and subsequently drawn into burrows. Spores of these fungi were found in the intestines of dissected specimens. Bezborodov (2009) and Bezborodov and Koshkin (2014a, b) reported that adults of *Bolbocerosomazonatum* (Nikolaev, 1973) were repeatedly found under dry horse and cow dung in the Far East of Russia, in cavities covered with white mould, thus suggesting that the adults are mycetophagous; no burrows were observed under the dung. In Australia, dissections of bolboceratines and analysis of their faeces were carried out mainly by Houston and Bougher (2010), who found that the intestines or excrement of adults of *Blackbolbus*, *Blackburnium*, *Bolboleaus* and *Bolborhachium* species contained large quantities of spores of various species of hypogeous fungi (e.g., of the genera *Amarrendia*, *Hysterangium*, and *Scleroderma*), as well as immature unidentified sporocarp tissue, unidentified ascomycetes, or glomeralean hyphae and spores with varying quantities of soil. These authors also reported that only six of 120 specimens of bolboceratines collected while in flight (i.e., those specimens taken at lights or from light traps), and only 34 of 114 bolboceratines collected from burrows had food in their intestines. It is likely that the beetles feed only intermittently and possibly spend protracted periods without a meal. In many cases, their burrows may serve to provide them only with shelter until their next foray. In several genera, Houston and Bougher (2010) found no food present in the gut, which they explained by suggesting that feeding for these beetles is likely episodic, governed by weather events, and timing may be the key to finding specimens feeding. In the case of *B.unicornis*, it was not possible to dissect the individuals to determine the intestinal contents due to its strict protection in all EU countries. In the burrows of some Australian species of the genera *Blackbolbus*, *Blackburnium*, *Bolborhachium*, and *Elephastomus*, pieces of sporocarps of *Scleroderma* sp., *Hysterangium* sp., and unspecified hypogeous fungi of the families Hymenogasteraceae and Clathraceae have been found, but with no eggs or larvae present in the vicinity (Howden et al. 2007; Houston and Bougher 2010). This suggests that these fungi were food for adults only. In burrows of both *B.gallicus* and Australian bolboceratines, adults of some species of round fungus beetles (Leiodidae) have been found on the sporocarps of hypogeous fungi (Béguin 1906; Howden et al. 2007). In the case of *B.gallicus*, this was *Leiodescinnamomea* (Panzer, 1793). Houston and Bougher (2010) reported that some *Scleroderma* sporocarps found in burrows of *Blackbolbusfrontalis* (Guérin-Méneville, 1838) were inhabited by numerous nitidulid beetles identified as *Thalycrodesmixtum* Kirejtshuk & Lawrence, 1992, and two sporocarps identified as *Hysterangium* sp. found in soil close to a burrow of *Blackbolbusfrontalis* were infested with nematodes. Mycetophagy of adults of the genus *Ochodaeus*, representatives of which were collected together with *B.unicornis*, was also recently confirmed (Huchet et al. 2022).

Immature stages have only been described in a few species of bolboceratines (Arens 1922; Ritcher 1947, 1966; Howden 1955, 1964; Verdú et al. 1998, 2004; Rahola Fabra 2004; Howden et al. 2007; Houston 2011, 2016). In *B.unicornis*, no immature stages are known, and consequently nothing is known about the larval diet. During the excavation of adults from their burrows, no immature stages were found, similar to the reported cases of excavations of North American bolboceratines (Manee 1908; Wallis 1928; Sim 1930). As for the European representatives of the genus *Bolbelasmus*, the larva has only been described in *B.brancoi* (as *B.bocchus*, Verdú et al. 1998), and *B.gallicus* (Verdú et al. 2004), but even in these species larval nutrition has not been elucidated. Eggs have been described and/or photographed in only a few species of bolboceratines (Arens 1922; Howden 1955; Rahola Fabra 2004; Howden et al. 2007; Houston 2011, 2016). They are surprisingly large compared to the size of the adults. For *B.gallicus*, Rahola Fabra (2004) reported egg dimensions to be 7.0–8.0 × 4.0 mm, but the egg photographed next to the female and scale was actually 7.0 × 4.6 mm, whereas the body length of the female was ca. 14 mm (calculated from the scale line). Howden et al. (2007) noted that two eggs of the Australian species *Bolborhachiumanneae* Howden, 1985 measured 6.5 × 6.2 mm and 7.3 × 6.4 mm while two eggs of the slightly larger *B.recticorne* (Guérin-Méneville, 1838) measured 7.2 × 5.9 mm and 8.1 × 6.5 mm. The largest female of *B.anneae* measured 15.1 mm in length, while the largest female of *B.recticorne* measured 18.8 mm in length. According to Houston (2011), the eggs of another Australian bolboceratine *Blackburniumreichei* (Guérin-Méneville, 1838) weighed 45–56% as much as the females that laid them and measured 9.5–10.5 × 7.5–9.0 mm. On the other hand, the eggs of the North American bolboceratine *Odonteusdarlingtoni* are not so large compared to the adults: they measure ca. 2.4 × 1.5 mm, whereas the adults are ca. 10 mm in length (Howden 1955). Similarly, for the European species *Od.armiger*, Arens (1922) reported the length of the egg to be 2.5 mm (body length of adults is usually 6–10 mm). Rahola Fabra (2004) reported that the females of *B.gallicus* have two ovaries, each composed of six ovarioles, as in other representatives of the family Geo­trupidae (cf. Ritcher and Baker 1974), with only one ovariole functioning at any time, in alternating cycles (cf. Willimzik 1930). The fecundity of females of *B.gallicus* is very low (probably one to four eggs in a lifetime), nevertheless, according to Rahola Fabra (2004), populations are relatively stable; he stated that the female of *B.gallicus* fixes its giant egg to the ceiling of a small egg-shaped brood cell using soil mixed with its own excreta. All the cells found by Rahola Fabra were empty, which means that they did not contain anything that could provide food for the future larvae. Similarly, Arens (1922) reported that the brood cells with eggs of *Od.armiger* contained no provision, but in a few cases he found pieces of unspecified fungi or humus in the burrows. Also, Miquel and Vasko (2014) found 16 empty brood cells of *Od.armiger*. The fact that the brood cells did not contain any material collected by females differs from what Howden (1955) observed in another species of the subfamily Bolboceratinae, where females lined their brood cells with material brought in from outside (surface humus, dried dung) that could be a food for the larva. In two species of the genus *Bolborhachium* the brood cells were filled with fine black humus, perhaps mixed with fungi (Howden 1985; Howden et al. 2007). In contrast, according to Houston (2016), the brood cells of some Western Australian bolboceratines, such as *Bolborhachiumrecticorne* and some congeners, were formed from darker surface soil, but no food was found. Eggs and larvae of these species collected in the field were reared in their original cells and in artificial cells made in soil. Of three instars, the first contained the already developed second instar and did not feed. Second and third instars nibbled at the walls of their cells as if feeding, grew in size, and increased their weight 2.5–3.0 times. However, they turned over little soil, ingested little solid material, and rarely passed faeces, so ingestion and digestion of ‘humus’ (finely divided plant detritus) is unlikely to account for all (if any) of their weight gain. As the contents of the larval intestine were hygroscopic, perhaps larvae ingest salts and/or humic and fulvic acids that enable them to absorb water (Houston 2016). This author also suggested that the soil bacteria may be a source of nutrition for the larvae. This is also consistent with Houston’s finding of two newly emerged adults of *Blackburniumreichei* in closed, earthen cells at depths of 60 and 72 cm, with no traces of faecal material or uneaten provision being observed in or near these cells (Houston 2011). The hypothesis that larvae of Australian bolboceratines do not ingest solid food is supported by the description of their morphology. Houston (2011) reported that compared with free-living scarabaeoid larvae (e.g., Melolonthinae, Dynastinae, Trogidae) where the head and mandibles are strongly sclerotised and the legs well developed with strong tarsal claws, the larvae of all known bolboceratines are degenerate. According to Houston (2011), the larva of *Blackbolbushoplocephalus* (Lea, 1916) provides the most extreme example of degeneration known to date. Its immobility and vestigial appen­dages (particularly its simple, feeble mandibles) suggested it was a non-feeding, resting stage. Importantly, though, the mandibles of the second instar (judging from its exuvia) were equally feeble and consistent with a no-feeding hypothesis (Houston 2011). Houston went on to point out another feature of the larvae of known bolboceratines: the relatively slender abdomen which contrasts with the swollen abdomen of many other Scarabaeoidea, suggesting at least a different feeding biology and possibly hinting at a reduction or even absence of feeding. The very simple form of the larval intestine found by Houston (2011) in *Blackburniumreichei* when compared with the intestines of larvae of other scarabaeids (e.g., Areekul 1957) and its emptiness are consistent with loss of feeding. Similarly, Higurashi and Tanahashi (2014) and Higurashi et al. (2019) recorded giant eggs, larvae, and pupae of *Bolbocerosomanigroplagiatum* excavated from a depth of ca. 80 cm, with the larvae having poorly developed appendages (mandibles and legs). Howden (1955) reported that larvae of the North American bolboceratines *Odonteusdarlingtoni*, *O.liebecki* (Wallis, 1928), and *Bolbocerosomafarctum* (Fabricius, 1775) feed on humus, carefully sifting it from a provision of humus-rich sand filling the lower ends of burrows. This is consistent with earlier observations by Robert J. Sim, who assumed that females of *Odonteussimi* (Wallis, 1928) lay their eggs in humus formed into an elongated mass at the lower ends of the burrows (Wallis 1928).

Since we have virtually no knowledge of the diet of adults or larvae of *B.unicornis*, we can only speculate on what its diet consists of. Given the findings of Australian and Japanese researchers on related species, the likely food of adults appears to be hypogeous fungi (spores, hyphae, and sporocarps), while the food of larvae could be fine soil humus and/or soil bacteria. The previous hypothesis that the larvae of *B.unicornis* feed on sporocarps of hypogeous fungi (e.g., Bartenev et al. 1997; Nádai 2006; Juřena et al. 2008; Kaděra 2017; Németh 2015; Nuß and Jäger 2020) is not supported by observation and seems very unlikely considering the observations in other species of bolboceratines. The larval morphology of the genus *Bolbelasmus* is very similar to that of larvae of the genera *Bolbocerosoma* and *Bolborhachium* (Verdú et al. 1998, 2004), which suggests a similar way of life, including feeding habits.

### ﻿Life cycles of bolboceratines

Life cycles have been documented for only a few bolboceratine species (Houston 2011, 2016). Houston (2016) found that for three Australian species (*Bolborhachiumrecticorne*, *Blackburniumreichei*, and *Bolboleaushiaticollis* Howden, 1985), the period between discovery of an egg and hatching of the larva was 15–35 days. According to Houston, duration of the larval stage in *Bolborhachiumrecticorne* ranged from 63 to 95 days (*n* = 6) and for one *Blackburniumreichei* larva, it was 44 days. One larva of *Bolboleaushiaticollis* pupated 81 days after being found while another (hatched from an egg) survived for at least 13 months before dying. Based on the Houston’s data, development from egg to adult in *Bolborhachiumrecticorne* could require 129–159 days or more. As newly emerged adults remained in their natal cells for at least 30 days while their integuments harden and darken, total development time (egg to active adult) might require 6 months or more, according to Houston. Houston (2016) suggested the possibility that in *Bolboleaushiaticollis*, mature larvae enter a dormant stage, thereby extending the development time even further. Houston (2011) recorded excavating a third larval instar of *Blackbolbushoplocephalus* that remained dormant for 105 days before pupating.

Assuming that the development of *B.unicornis* is similar, it is very likely that only adults, both old and newly emerged, overwinter. This assumption is supported by numerous records where both old, dark-coloured individuals with heavily abraded teeth of fore tibiae and fresh, pale-coloured individuals with sharp protibial teeth have been recorded at the beginning of the season (pers. obs.). Some bolboceratines have overlapping generations. For example, in the genus *Odonteus*, eggs, larvae, pupae, and adults have been observed together in a single branching burrow (Jameson 2002; Staines and Staines 2020).

### ﻿Seasonal dynamics of *B.unicornis*

**Figure 19.**
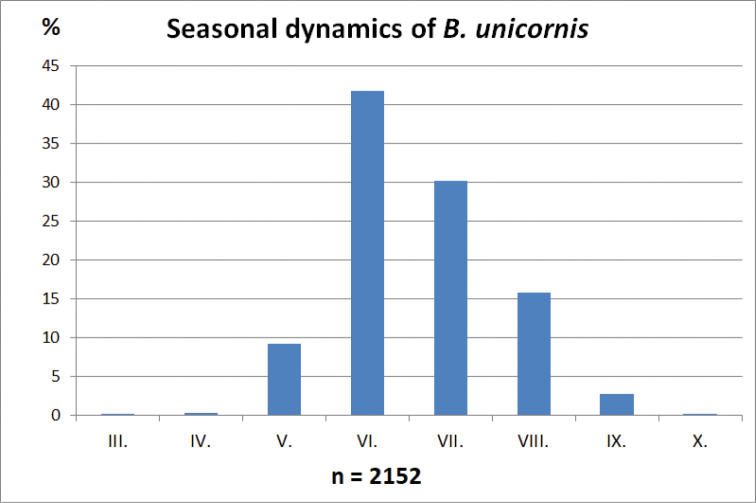
Seasonal dynamics of *B.unicornis* (the number of specimens from each country included in these statistics: Slovakia – 1392, Hungary – 350, Ukraine – 168, Serbia – 87, Romania – 79, Austria – 44, Czech Republic – 31).

In the Pannonian Basin, the centre of the distribution of *B.unicornis*, adults are active from May to September, exceptionally as early as April and as late as October, with a significant peak in June and the first half of July (Fig. 19). Very few data are available from the other parts of the distribution area. It appears that in the southernmost part of the range, adults may be active as early as March, which is supported, for example, by the record from East Thrace (see Hillert et al. 2016 and Faunistic records in this study). The seasonal dynamics of the species are always significantly influenced by precipitation changes during the year. It is likely that only the adults overwinter, as reported by Caillol (1913) for the congeneric species *B.gallicus* (see also Rahola Fabra 2004).

### ﻿Habitat preferences of *B.unicornis*

*Bolbelasmusunicornis* is a stenotopic species, characteristic of Pannonian steppes, forest-steppes and sparse deciduous forests, especially dominated by oaks. It is often found on sandy substrate (e.g., surroundings of Győr, Kiskunság National Park), sandy-loess substrate (e.g., localities around the village of Čejč), gravelly-sandy-loess substrate (e.g., Dunajské luhy Protected Landscape Area), loess substrate (e.g., wider surroundings of Štúrovo, Cerová vrchovina Mts, Gödöllő Hills, Dniester Canyon National Nature Park) or limestone substrate (e.g., Slovak Karst, Aggtelek National Park, some localities around Budapest). Characteristic habitats are steppe or forest-steppe pastures at the edges of oak forests (e.g., Cerová vrchovina Mts, Slovak Karst), of oak-beech or beech forests (e.g., Slanské kopce hills, Đerdap National Park, some localities in Romania), oak-hornbeam or hornbeam forests (e.g., Pirot District of Serbia, Kaniv Nature Reserve in Ukraine), and of shrub zones (with e.g., *Crataegusoxyacantha*, *Prunusspinosa*, and *Rosacanina*). In the Kiskunság National Park it occurs in the Pannonic sand dune thicket (*Junipero-Populetumalbae*) (Merkl 2014). Occurrences of *B.unicornis* in completely treeless habitats are known for the northern half of its range (Czech Republic, Slovakia, Hungary), while further south the species occurs in areas of more extensive forest cover (Serbia, Romania, Bulgaria). In eastern Ukraine (Dnipro City) the species occurs in sparse oak forest (Fig. 15). Also, the only known recent record from Croatia was made in forest (Koren 2017). Other typical habitats are remnants of the native steppe grasslands between vineyards, former steppe and forest-steppe pastures, remnants of steppe or forest-steppe in agricultural landscapes that have been preserved due to their inaccessibility to agricultural machinery (e.g., localities around Čejč and many sites in Hungary). In the case of hilly terrain, *B.unicornis* mainly prefers south- and southwest-facing slopes, and less frequently slopes inclined to the southeast or even to the north (e.g., Fruška Gora National Park – see Faunistic records in this study, and Gorna Malina – see Zaharieva-Stoilova 1974).

It seems that the fundamental requirement of the species is natural vegetation cover and soil undisturbed by agriculture. The same was noted by Sajó (1897), who stated that the species occurred in a dry oak forest on a hill near Kis-Szent-Miklós (currently Őrbottyán – Őrszentmiklós), but it disappeared as soon as the hill was converted to farmland. The species is probably also very sensitive to the use of chemicals in agriculture and forestry. It seems to be threatened by the overgrowth of invasive plant species such as *Robiniapseudoacacia* or *Ailanthusaltissima* in steppe and forest-steppe habitats. Furthermore, the extensive removal of shrubs such as *Crataegus* sp., *Rosacanina*, *Corylusavellana*, and trees (e.g., *Quercus* spp., *Populus* spp., *Prunusspinosa*) and taller herbaceous plants, as well as too intensive sheep grazing seem to have negative effects on the presence of *B.unicornis* (cf. also Németh 2015). The largest known population of the species in Europe at the Panský diel site on Kopáč Island near Bratislava, Slovakia (Figs 4, 5) was severely decimated by the inappropriate conservation management of the site (pers. obs., cf. also Majzlan 2020).

Central European sites with substantial populations of *B.unicornis* are characterised by the occurrence of plant species such as *Quercus* spp., *Crataegusoxyacantha*, *Prunusspinosa*, *Rosacanina*, *Festuca* spp., *Thymus* spp., *Orobanche* spp., *Scabiosaochroleuca*, *Euphorbiacyparissias*, *Achilleamillefolium*, and *Artemisia* spp.

On describing the potential habitat of the related *B.gallicus* in southern France, Rahola Fabra (2004) lists the following plant species: *Quercusilex*, *Q.coccifera*, *Q.pubescens*, *Oleaeuropaea*, *Pinushalepensis*, *P.pinea*, *Juniperusoxycedrus*, *Buxussempervirens*, *Cistusmonspeliensis*, *C.albidus*, *Genistahispanica*, *Brachypodiumretusum*, *Thymusvulgaris*, *Sedum* sp., *Coronillaglauca* and *Viburnumtinus*.

The Coleoptera that co-occur in Central European localities with *B.unicornis* include *Lethrusapterus* (Laxmann, 1770), *Odonteusarmiger*, *Ochodaeuschrysomeloides*, *O.integriceps*, *Gymnopleurus* spp., *Carabusmontivagus* Palliardi, 1825, *C.scabriusculus* Olivier, 1795, *Capnodistenebrionis* (Linnaeus, 1761), *Perotislugubris* (Fabricius, 1777), *Ptosimaundecimmaculata* (Herbst, 1784), *Sphenoptera* spp., and *Agrilusalbogularis* Gory, 1841 (observations by many collectors including the author).

The elevation of the sites where *B.unicornis* has been recorded varies between 20 and 800 m a.s.l. The average altitude of all known localities for which it could be at least approximately determined (*n* = 351) is 220 m a.s.l. It is therefore a species of lowland and lower hills.

## ﻿Monitoring methods

The most effective method for monitoring this species is to capture adults during their flights after sunset with a net using a flashlight, preferably a headlamp, in suitable microhabitat. This collecting method was employed as early as the 1920s by Rudolf Čepelák (see Čepelák 1925). The effectiveness of this method is evidenced by the large number of specimens collected by him, which are still scattered in numerous collections of museums and private collectors (see Faunistic records). Typically, the flights of beetles occur in very limited areas, and the concentration of flying individuals can vary considerably from place to place. For example, more flying beetles can be observed above grassy trails with ruts made by agricultural machinery or above paths trodden by humans or animals. Flying beetles can also be detected by the hum of their wings similar to that of a flying European hornet (*Vespacrabro* Linnaeus, 1758), as reported by Čepelák (1925) and Roubal (1936). However, sometimes the beetles fly almost noiselessly (pers. obs.).

Alternatively, during the day, one may find the beetles in their burrows, which are indicated by small piles of excavated soil at the entrances (so called “push-ups”). These push-ups are similar to those of some large ground-nesting bees but the push-ups of the bees are conical, composed of uniformly loose soil about a central entrance, whereas the soil pushed up by the earth-borer beetles tends to form an irregular pile of lumps (Figs 5E, 8A–D, 15E, F). If the individual is present in the burrow, the entrance is often covered by a pile of excavated soil. An uncovered hole usually indicates that the beetle is no longer present. Similarly, if a push-up is weathered down, it is usually old and the beetle may no longer be present in the burrow. This method is less effective on grassy sites as the push-ups may be screened from view.

Light trapping appears to be ineffective to capture this species (cf. also Cséfalvay 2015), as the beetles show light-aversion during flights and avoid light sources. Only occasional specimens which may be long-distance flyers come to the light later in the night. On very rare occasions, a few individuals have been observed flying to the light (on an illuminated canvas) just after sunset. It is likely that the beetles respond diffe­rently to different light sources, something requiring further research.

*Bolbelasmusunicornis* is also difficult to find due to the fact that observable activity of adults (flights and digging underground tunnels with push-ups) occurs only after heavy rains, when the soil is damp and loose enough for the beetles to burrow easily. During the dry periods, when the soil is hard, and also in winter, the beetles are buried deeper in the ground and show no above-ground activity, making it very difficult to find them.

### ﻿Excerpt from the diary of Rudolf Čepelák

**Figure 20.**
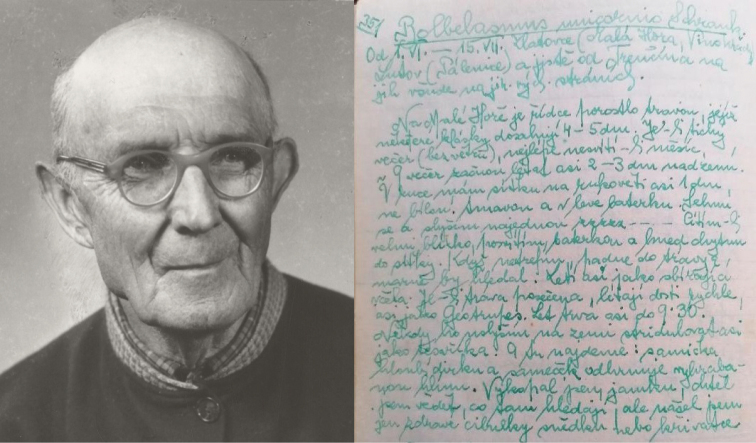
Rudolf Čepelák (born 16 April 1886 in Kutná Hora, Austria-Hungary, died 21 December 1972 in Český Brod, Czechoslovakia) and an excerpt from his diary with notes on collecting of *B.unicornis*.

Below is the translation of a passage on *B.unicornis* from the diary of the excellent Czech coleopterist Rudolf Čepelák (1886–1972; Fig. 20), the discoverer of an efficient method of collecting this beetle. This text was written in the second half of the 1960s (Svätopluk Čepelák pers. comm., 2021). Čepelák here supplemented and specified his previously published observations (Čepelák 1925) from sites north of Zlatovce near Trenčín, where he worked as a teacher in 1923–1939 (see Koleška 1979):

“*Bolbelasmusunicornis Schrank*

*From 1.vi. to 15.vii. Zlatovce (Malá hora hill; Vinohrady), Ľutov (Pálenice hill), and certainly from Trenčín southwards everywhere on the south-eastern slopes.*

*The area of Malá hora is sparsely covered with grass, which reaches 40–50 cm in places. If it is a quiet evening (no wind), preferably without moonlight, at 9 pm they start flying about 20–30 cm above the ground. In my right hand I have a net with a handle about 10 cm long, not white but dark, and in my left hand a torch. I bend down and suddenly hear ‘zzzzz...’. If I feel it’s very close, I shine the torch and immediately catch it with the net. If I miss, it falls into the grass, where I would look for it vainly. It flies like a bee collecting pollen. If the grass is cut, they fly quite fast, like*Geotrupes. *The flights continue until ca. 9.30 pm. Sometimes I hear it on the ground, stridulating like a longhorn beetle. And then we see: the female digging a hole and the male removing away the excavated soil. I dug a hole to see what they were looking for, but all I found were healthy*Ornithogalum*or*Gagea*bulbs*.”

**Figure 21.**
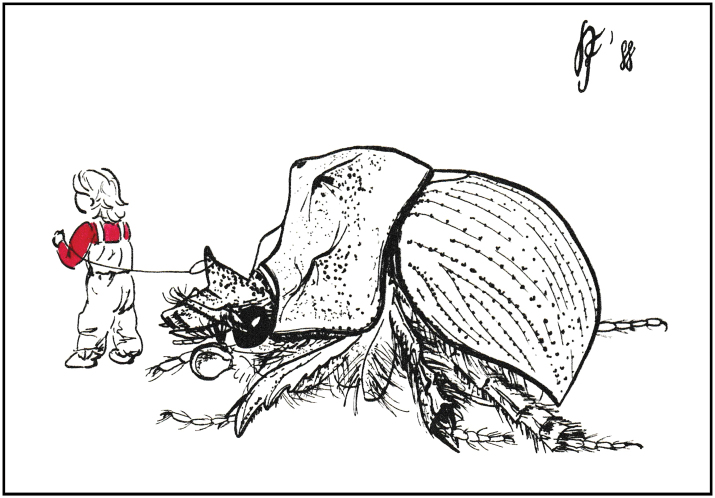
Drawing by Regina & David Král with the motif of *B.unicornis*, sent as PF 1988.
