# ﻿Addenda and corrigenda: Juřena D (2022) ﻿A critical review of the distribution of the endangered European earth-borer beetle *Bolbelasmusunicornis* (Coleoptera, Geotrupidae), with new records from 13 countries and observations on its bionomy. ZooKeys 1105: 1–125. https://doi.org/10.3897/zookeys.1105.81474

**DOI:** 10.3897/zookeys.1148.100501

**Published:** 2023-02-20

**Authors:** Daniel Juřena

**Affiliations:** 1 Lidická 59, 796 01 Prostějov, Czech Republic Unaffiliated Prostějov Czech Republic

**Keywords:** Bolboceratinae, distribution maps, Europe, faunistic records, Western Palaearctic

## Abstract

The author provides corrections of minor errors and omissions from his initial study, as well as data from omitted and new literature, and new records based on the material studied and new observations. For some of the previously published records, details obtained subsequently by the author are added. The first record of *Bolbelasmusunicornis* for Belarus is given, representing the northernmost known occurrence of the species. The second recent record for Croatia is quoted from an internet source. Updated distribution maps are provided for the Czech Republic and Slovakia, and for the entire range, as well as a distribution map of the Western Palaearctic representatives of the *B.unicornis* species group. The species is currently known from 386 localities in 20 countries.

## ﻿Introduction

The primary study on the distribution and bionomy (ecology) of *Bolbelasmusunicornis* (Schrank, 1789) (Juřena 2022) contained a number of minor errors and shortcomings which are corrected here. Some previously omitted literature data are added, namely by Bertolini (1875), Anonymus (1876), Schilsky (1888, 1909), Petri (1910), Reisser (1954), and Hoffmann et al. (1955). References to newly published papers with records of *B.unicornis* are also added: Glerean et al. (2021), Sheshurak et al. (2022), Theves and Bittner (2022), Vasyliuk (2022), and Byk et al. (2023). A reference to the second record of the species for the territory of Croatia, posted on the Facebook website (Association Hyla 2022), is also provided.

New records are presented based on material and observations obtained by the author just after his initial study (Juřena 2022) was published. Most significant is the record of *B.unicornis* from southeastern Belarus, which represents the first reliable record for this country and the northernmost known point of occurrence for the species. Records from new localities in Hungary (Csopak; Lábatlan; Kazár) and Slovakia (Mužla-Čenkov; Gemerské Dechtáre) are also provided. The updated distribution maps are shown in Figs 1, 2. Table 1 shows the number of known localities with *B.unicornis* for each country. These data show that almost half of all known localities where the species has been recorded after 1999 are located in Hungary.

**Figure 1.**
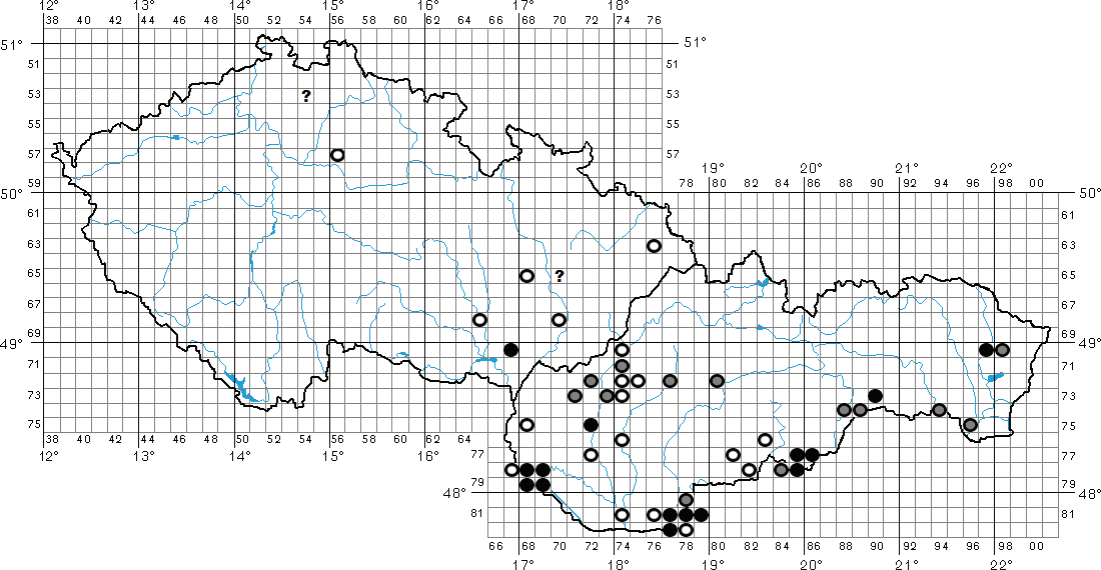
Distribution of *Bolbelasmusunicornis* in the Czech Republic and Slovakia (open circles = records before 1960; open circles with a grey centre = records between 1960–1999; solid circles = records after 1999; a question mark indicates a dubious record).

**Figure 2.**
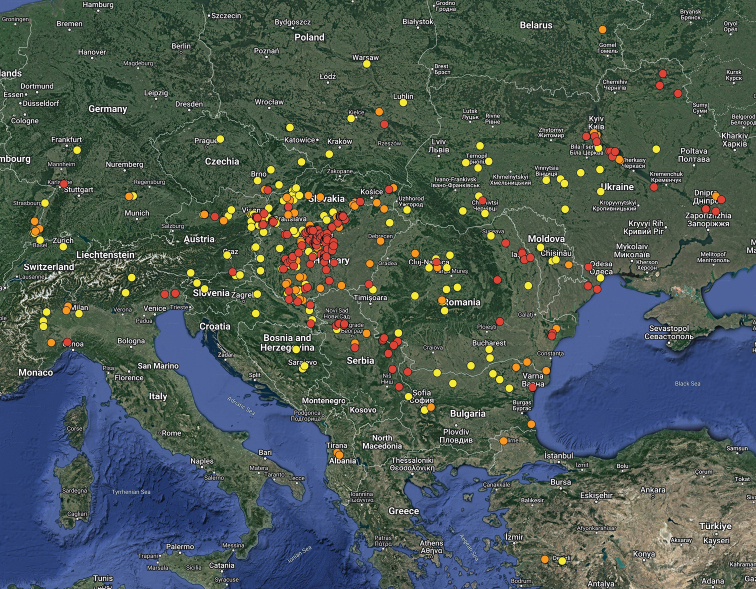
Distribution of *Bolbelasmusunicornis* (yellow circles = records before 1950, orange circles = records between 1950–1999, red circles = records after 1999).

**Table 1.** Number of known localities with *Bolbelasmusunicornis* in each country (**loc** = number of localities; **locr** = number of localities with recent occurrence, i.e., 2000–present; a cross indicates country where the author considers the species to be extinct).

Country	loc	locr
Hungary	132	74
Slovakia	54	20
Ukraine	43	18
Romania	37	14
Austria	32	9
Serbia	13	9
Italy	12	3
Bulgaria	11	2
† Czech Republic	9	1
† Poland	7	1
Croatia	6	2
† France	6	0
Germany	5	1
Moldova	4	0
Slovenia	4	0
Bosnia and Herzegovina	3	0
Turkey	3	0
Albania	2	0
† Switzerland	2	0
Belarus	1	0
**Total**	**386**	**154**

Fig. 3 shows the distribution of all Western Palaearctic representatives of the *unicornis* species group with the exception of *B.tauricus* Petrovitz, 1973, the validity of which was questioned by Miessen (2011) on the basis of a study of type material (two paratypes were found to belong to *B.nireus* Reitter, 1895, the holotype is unclear whether it is a form of *B.nireus* or a different species).

**Figure 3.**
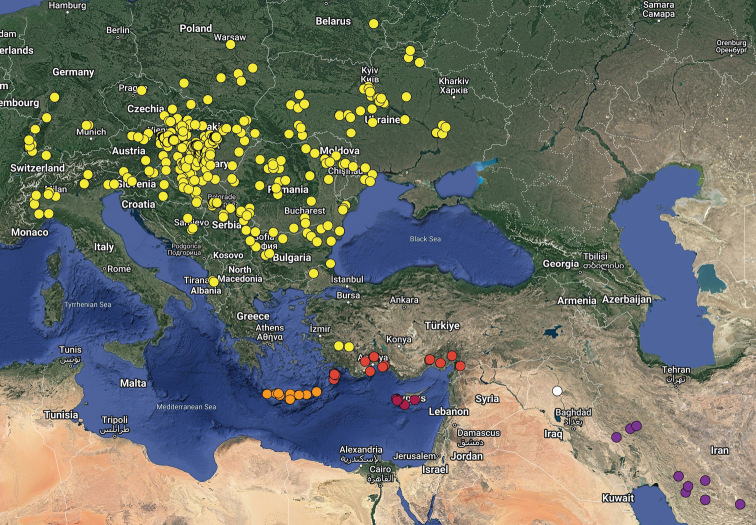
Distribution of the Western Palaearctic representatives of the *Bolbelasmusunicornis* species group: yelow circles = *B.unicornis*; orange circles = *B.keithi* Miessen & Trichas, 2011; pale red circles = *B.nireus*; dark red circles = *B.makrisi* Miessen, 2011; purple circles = *B.zagrosensis* Sommer, Hillert, Hrůzová & Král, 2021; white circle = record of a specimen tentatively assigned to *B.nireus* by Krikken (1977), but in need of revision (it could be *B.zagrosensis*).

## ﻿Materials and methods

Within Errata, only lines of text (including headings), not spaces, are numbered; figure legends are not included in the numbering. The faunistic records are divided into paragraphs according to the largest superior administrative units or traditional regions. The countries, administrative units/traditional regions, and faunistic records are ordered according to their geographical position from east to west and from north to south. A question mark at the beginning of a faunistic record indicates dubious data. The following acronyms are used in the text: **CEST** = Central European Summer Time, **FSLG** = flying slowly low above the ground, **FMF** = faunistic map field used in grid mapping of fauna and flora in Central Europe (Fig. 1; see also e.g., Zelený 1972; Novák 1989; Pruner and Míka 1996; Kolouch 2002), **PP** = Přírodní památka/Prírodná pamiatka (Natural Monument), **PR** = Přírodní rezervace/Prírodná rezervácia (Nature Reserve). Unless otherwise stated, the material has been identified or revised by the author.

The following systems are used to transliterate cited literature and geographical or personal names in the Cyrillic scripts: BGN/PCGN 1979 system for Belarusian, BGN/PCGN 1947 System for Russian, BGN/PCGN 2005 Agreement for Serbian, and BGN/PCGN 2019 system for Ukrainian.

For the distribution map of the Czech Republic and Slovakia, the records are divided into three time periods: pre-1960, 1960–1999 and post-1999 (Fig. 1). This map was compiled by manually placing the circles in the grid map used for faunistic research in these countries in standard free graphics software. For the distribution map of Europe, the following time periods are used: pre-1950, 1950–1999 and post-1999 (Fig. 2); the distribution map of the Western Palaearctic representatives of the *unicornis* species group was compiled using data contained in Krikken (1977), Lodos et al. (1999), Petersen et al. (2006), Miessen (2011), Miessen and Trichas (2011), Hillert et al. (2016), Sommer et al. (2021) and Juřena (2022). Both of the latter maps were created using the Google Maps web application by inserting specific GPS coordinates into the system. In cases where the exact GPS coordinates were not known (e.g., records from literature), the midpoint GPS coordinates of the village, town, county, or area were used. The definition of the Western Palaearctic is adopted from Mitchell (2017).

Table 1 with the number of localities with the occurrence of *B.unicornis* for each country is based on the data provided by Juřena (2022) and those in this paper.

### ﻿Acronyms for the collectors, observers, and institutes

**ABW** Adam Byk, Warsaw, Poland

**BKL** Bence Krajcsovszky, Lábatlan, Hungary

**BLZ** Boris Lauš, Zagreb, Croatia

**BPK** Balázs Pintér, Kerepes, Hungary

**DJP** Daniel Juřena, Prostějov, Czech Republic

**DKP** David Král, Praha, Czech Republic

**FPT** Filip Pavel, Týniště nad Orlicí, Czech Republic

**FTT** Filip Trnka, Tršice, Czech Republic

**GDK** Gejza Dunay, Kráľovce, Slovakia

**KHE** Krisztián Harmos, Eger, Hungary

**LFS** Lukáš Fiala, Sázava, Czech Republic

**MBK** Marek Bidas, Kielce, Poland

**MSC** Miroslav Snížek, Homole near České Budějovice, Czech Republic

**MSP** Milan Sláma, Praha, Czech Republic

**OBL** Olivier Boilly, Lille, France

**OSD** Oleksandr Oleksiiovych Sukhenko (Олександр Олексійович Сухенко), Dnipro, Ukraine

**RFS** Rudolf Fiala, Sázava, Czech Republic

**RGZ** Radim Gabriš, Zlaté Hory, Czech Republic

**RVO** † Radovan Veigler, Olomouc, Czech Republic

**SET** Sebastian Tylkowski, Kraków, Poland

**VGG** † Vadim Gennad’yevich Grachëv (Вадим Геннадьевич Грачёв), Moscow, Russia

**VNP** Vladimír Novák, Praha, Czech Republic

**ALMD** Aquazoo Löbbecke Museum, Düsseldorf, Germany

**MNHT** Civic Museum of Natural History, Trieste, Italy

**NHRS** Entomological Collections of the Swedish Museum of Natural History, Stockholm, Sweden

**NMOK** Naturkundemuseum im Ottoneum, Kassel, Germany

**NMPC** National Museum, Prague, Czech Republic

**SGGW** Department of Forest Protection, Institute of Forestry Sciences, Warsaw University of Life Sciences, Warsaw, Poland

**ZMMU** Zoological Museum of the Moscow Lomonosov State University, Moscow, Russia

## ﻿Errata

**Page 1, line 2 of Abstract**: “377 localities” should read “378 localities”.

**Page 1, line 3 of Abstract**: “152 localities” should read “153 localities”.

**Page 3, line 5**: among the literature cited giving the body length of *B.unicornis* adults to be 12–15 mm, three references are missing: Reitter (1892, 1909), Klapálek (1903).

**Page 3, line 21**: missing citation of Panzer (1813)—see references in this paper.

**Page 3, line 28**: missing citation of Bremi-Wolf (1856)—see references in this paper.

**Page 3, line 30**: missing citation of Stierlin (1893)—see references in this paper.

**Page 3, line 40**: in relation to hypogeous fungi as the presumed food of *B.unicornis*, Ohaus (1929) is erroneously listed among the cited literature—this reference should be deleted.

**Page 4, line 11**: a new reference Price and Ratcliffe (2023) may be included among the literature cited in connection with the listing of Bolboceratinae as a subfamily of Geotrupidae.

**Page 8, line 10**: a comma is missing after the name of Luciano Ragozzino.

**Page 8, line 25**: the acronym for Milan Sláma should read MSP instead of MPP.

**Page 13, line 33**: “*Od.armiger*” should read “*Odonteusarmiger* (Scopoli, 1772)”.

**Page 14, legend to Fig. 1**: five missing commas after “female” and “male”.

**Page 15, line 9**: “PR Čejkovické Špidláky reserve” should read “PP Čejkovické Špidláky reserve”.

**Page 15, line 16**: “*Odonteusarmiger* (Scopoli, 1772)” should read “*Od.armiger*”.

**Page 17, line 17**: “Ernő Csiki obs.” should read “Ernő Csiki leg.”.

**Page 18, line 28**: “Ernő Csiki obs.” should read “Ernő Csiki leg.”.

**Page 19, line 31**: “(Juřena et al. (2008)” should read “(Juřena et al. 2008)”.

**Page 19, line 41**: “*Och.chrysomeloides*” should read “*Ochodaeuschrysomeloides* (Schrank, 1781)”.

**Page 20, line 24**: the acronym for Milan Sláma should read MSP instead of MPP.

**Page 22, line 7**: “*Ochodaeuschrysomeloides* (Schrank, 1781)” should read “*Och.chrysomeloides*”.

**Page 30, line 40**: “21.10–1.40 CEST” should read “21.10–21.40 CEST”.

**Page 35, line 1**: missing question mark before “Upper Bavaria”.

**Page 36, line 9**: the cited literature listing *B.unicornis* from the Canton of Ticino is missing Huber (1916).

**Page 40, line 34**: missing “leg.” after A. Liana’s name.

**Page 43, line 26**: “Wien Umg.,” should read “Wien Umg.”.

**Page 44, lines 11 and 16**: “Ernő Csiki obs.” should read “Ernő Csiki leg.”.

**Page 47, line 22**: «“Pest“» should read «“Pest”».

**Page 49, line 15**: the acronym for Gergely Petrányi should read GPB instead of GBP.

**Page 51, lines 34–35**: “Vadász Csaba” should read “Csaba Vadász” and use the abbreviation CVK.

**Page 53, line 5**: “68 Hungarian localities” should read “69 Hungarian localities”.

**Page 54, line 22**: “(Република Српска)” should be in bold.

**Page 58, line 4**: “Ernő Csiki obs.” should read “Ernő Csiki leg.”.

**Page 59, line 5**: the record from Hammersdorf (= Sibiu-Gușterița) is missing “1 ♂”.

**Page 61**: there should be no space between lines 4 and 5.

**Page 64, line 4**: “1 ♀ in Hartmann [leg.]” should read “1 ♀, Hartmann [leg.]”.

**Page 67, line 36**: “ans” should read “and”.

**Page 71, lines 20–21**: “*Bolbelasmuskeithi*Miessen and Trichas 2011” should read “*Bolbelasmuskeithi* Miessen & Trichas, 2011”.

**Page 72, legend to Fig. 18**: “B.unicornis” should be in italics.

**Page 73, line 3**: “...locality is Mulhouse” should read “...localities are Colmar and Mulhouse”.

**Page 77, line 30**: the reference “Ohaus 1929” should be deleted.

**Page 83, line 38**: *Knautiaarvensis* is missing among the plants characteristic of Central European localities with *B.unicornis*.

**Page 90, line 20**: for Ballerio (2008), “...di Ferrara, 18:” should read “...di Ferrara 18:”.

**Page 100, line 4**: “83–83.” should read “p. 83.”.

**Page 102, line 25**: for Huchet et al. (2022), “Journal of Applied Entomology 00: 1–6” should read “Journal of Applied Entomology 146: 911–916”.

**Page 102, line 30**: for Hudeček (1928), “Natural History” should read “National History”.

**Page 102, lines 34–36**: for Hudeček (1930), the translation of the title should be as follows: “[National history of central and northern Moravia. National history of the Olomouc County. Part I. Natural monuments of central and northern Moravia. National history handbooks. Part I.]”.

**Page 104, line 28**: for Kaufmann (1914b), “Baranyavámegye” should read “Baranyavármegye”.

**Page 109, line 36**: for Majzlan (2020), the page range should read 71–101 instead of 47–70.

**Page 110, line 15**: for Manolache (1930), the page range should read 3–20 instead of 18–20.

**Page 113, lines 36–38**: the reference Ohaus (1929) should be omitted.

**Page 119, lines 2–3**: “7pp. 710–1390” should read “710–1390”.

## ﻿Additions

**Faunistic data**

### ﻿France

**Published data**

“Elsass” [= Alsace], no other data (Schilsky 1909).

**Material examined**

? “Savoie” [= Savoy, a cultural-historical region of France], 1 ♀ with no other data, coll. ALMD; since this is a mountainous area that does not meet the ecological requirements of the species, it is likely that this is a confusion of locality.

**Grand Est**, Haut-Rhin, Mulhouse, 1 ♂ with no other data, OBL det. + coll.

### ﻿Germany

**Published data**

**Bavaria (Bayern)**, no other data (Schilsky 1888, 1909).

The first record of *B.unicornis* from Germany after 54 years from Bruchsal, Baden (see Juřena 2022) was published simultaneously with full details by Theves and Bittner (2022).

### ﻿Italy

**Published data**

**Piedmont (Piemonte)**, Provincia di Alessandria, Lerma—all records from this locality published by Juřena (2022) were previously published in a poster for the XXVI Italian National Congress of Entomology (7–11 June 2021) by Glerean et al. (2021).

**Trentino-Alto Adige/Südtirol**, “Trient” [= Trento], end of October 1875, number of specimens not specified, plant materials alluviated by the flooded Adige River, Stefano de Bertolini leg. (Bertolini 1875); this is Bertolini’s second record from Trento (see Bertolini 1874).

**Material examined**

“Ital” [= Italy], 1 ♂ [ex coll.] Dohrn, coll. NHRS (catalogue number of the specimen: NHRS-ALJB000000381).

### ﻿Austria

**Published data**

? **Tyrol (Tirol)**, no other data (Schilsky 1888, 1909)—this record does not seem likely given that this is a high mountain region, which does not correspond to the ecological requirements of *B.unicornis*.

**Upper Austria (Oberösterreich)**, Linz-Ebelsberg, bank of the badly flooded Traun River, [10.vii.1954], 28 spec., F. Linninger leg. (Reisser 1954—this is the first published mention of this record with the correct name of the collector and the exact number of specimens found, something that was missing in subsequent publications: Hoffmann et al. 1955; Franz 1974; Mitter 2000; Schwarz 2008; Juřena 2022); area between Pulgarn und Steyregg, August 1875, 1 ♂ and 1 ♀, A. Mader (Linz) leg., coll. Museum Francisco-Carolinum, Linz (Anonymus 1876—this is the first published mention of this record with the name of the collector and the exact number of specimens found, something that was missing in subsequent publications: Dalla Torre 1879; Schwarz 2008; Juřena 2022).

**Carinthia (Kärnten)**, no other data (Schilsky 1909).

**Styria (Steiermark)**, no other data (Schilsky 1888, 1909).

**Material examined**

**Vienna (Wien)**, “Wien, Umg.” [= Vienna env.], 1 ♂ (ex coll. Mikhail Klavdievich Tikhonravov, 1900–1974), undated, A. Winkler [probably leg.], coll. ZMMU.

### ﻿Slovakia

**Published data**

**Prešov Region (Prešovský kraj)**, Snina District (okres Snina), Snina, July 1965, 1 ♂ flew through an open window into a room (hostel of the Snina Forestry Plant on the outskirts of the town) after sunset, together with *Odonteusarmiger* (Scopoli, 1772), MSP leg., coll. DKP deposited in NMPC (Hillert et al. 2016; Juřena 2022)—specification of this record by Milan Sláma (pers. comm., 2022).

**Material examined and new observations**

**Bratislava Region (Bratislavský kraj)**, Bratislava II District (okres Bratislava II), Bratislava-Podunajské Biskupice, Kopáč Island, PP Panský diel (e.g., 48°6'4.22"N, 17°9'41.05"E; 48°6'1.44"N, 17°9'47.32"E; 48°6'3.33"N, 17°9'47.73"E; 48°6'5.44"N, 17°9'47.13"E; 48°6'5.94"N, 17°9'52.68"E), FMF No. 7868, ca 132 m a.s.l., 18.vi.2022, 3 spec. (2 ♂♂, 1 ♀) excavated from their burrows on the edge of a forest path, and 26 spec. (15 ♂♂, 11 ♀♀) flying < 0.5 m above the ground at 21.30–21.50 CEST (sunset: 20.53 CEST), no wind, 20°C, DJP, FTT & RGZ obs.

**Trenčín Region (Trenčiansky kraj)**, Trenčín District (okres Trenčín), Trenčín [env., Malá hora hill (48°54'43"N, 18°0'30"E), ca 230 m a.s.l., or Vinohrady (48°54'47.22"N, 18°1'4.68"E), ca 250 m a.s.l., FMF No. 7074], undated, [Rudolf] Čepelák [leg.], 1 ♂ in coll. ALMD, 1 ♂ (ex coll. Egon Lekeš) in coll. ZMMU; Trenčín District (okres Trenčín), “Trencsen, Ungarn” [= Hungary, Trenčín], FMF No. 7174, 1 ♂ and 1 ♀ with no other data, coll. ALMD.

**Nitra Region (Nitriansky kraj)**, Nové Zámky District (okres Nové Zámky), Mužla-Čenkov env., outside edge of the flood barrier of the Danube River, 47°46'24.52"N, 18°33'21.52"E, FMF No. 8277, 108 m a.s.l., 12.vi.2022, 1 ♂ FSLG at 21:45 CEST (= 62 min after sunset), together with *Od.armiger* (Scopoli, 1772), 20.vi.2022, 1 ♀ FSLG at 21:25 CEST (= 38 min after sunset), together with *Ochodaeuschrysomeloides* (Schrank, 1781), an anonymous observer from the Czech Republic obs.; Kamenica nad Hronom, [Čierna hora hill], FMF No. 8178, 6.vi.2010, 1 ♂, LFS & RFS leg., OBL det. + coll.

**Banská Bystrica Region (Banskobystrický kraj)**, Rimavská Sobota District (okres Rimavská Sobota), Cerová vrchovina Mts, Hajnáčka env., 48°13'41.56"N, 19°58'10.57"E, FMF No. 7785, ca 350 m a.s.l., 14.–15.vii.1984, 2 ♀♀ FSLG after sunset, GDK leg. + det., storage of the specimens unknown; Rimavská Sobota District (okres Rimavská Sobota), Cerová vrchovina Mts, Hajnáčka [env., ca 48°13'43.88"N, 19°58'15.53"E, FMF No. 7785, ca 380 m a.s.l.], 10.vi.1989, 1 ♂ FSLG after sunset, 27.vi.1989, 1 ♀ FSLG after sunset, RVO leg., coll. Ulrich Schaffrath deposited in NMOK (these specimens are part of the findings already published by Juřena et al. 2008); July 1990, 1 ♂, VNP leg., coll. Ulrich Schaffrath deposited in NMOK; Rimavská Sobota District (okres Rimavská Sobota), Cerová vrchovina Mts, Gemerský Jablonec, FMP No. 7785–7885, 7.vii.2013, 1 ♂, FPT leg., OBL det. + coll.; Rimavská Sobota District (okres Rimavská Sobota), Cerová vrchovina Mts, Gemerské Dechtáre env., 48°14'38.31"N, 20°0'56.32"E, FMF No. 7786, ca 240 m a.s.l., 27.vi.1987, 1 ♂ and 1 ♀ FSLG after sunset, GDK leg. + det., storage of the specimens unknown; Rimavská Sobota District (okres Rimavská Sobota), Cerová vrchovina Mts, Jestice env., Drienkové, 48°12'38.25"N, 20°2'54.45"E, FMF No. 7786, ca 230 m a.s.l., 6.vii.2020, 2 ♂♂ and 1♀ FSLG after sunset, GDK obs.; Rimavská Sobota District (okres Rimavská Sobota), Cerová vrchovina Mts, Jestice env., Drienkové, 48°12'40.07"N, 20°2'53.01"E, FMF No. 7786, 235 m a.s.l., 6.vii.2020, 1 ♂ flying in sunlight ca 0.5 m above the ground at about 19.00 CEST (sunset: 20:42 CEST), an anonymous observer from Slovakia obs.

### ﻿Hungary

**Material examined and new observations**

**Central Transdanubia (Közép-Dunántúl)**, Veszprém County (Veszprém vármegye), Csopak env., ca 46°59'10.83"N, 17°54'39.43"E, ca 265 m a.s.l., 14.v.2022, 1 ♂, Botond Balogh obs. + photo; Komárom-Esztergom County, Lábatlan, ca 200 m a.s.l., 31.v.2022, 1 ♀, at light, BKL obs. + photo.

**Central Hungary (Közép-Magyarország)**, Pest County (Pest vármegye), Verőce, 47°50'36.895"N, 19°2'34.01"E, 140 m a.s.l., 10.vi.2022, 1 ♀, at light at ca 21.00 CEST, BPK obs. + photo; Budapest, 1 ♂ and 1 ♀ (ex coll. Carl Bartels [1823–1901]) with no other data, coll. NMOK; Pest County, Csomád, Öreg-hegy, [ca 47°39'22.2"N, 19°12'42.3"E, ca 220 m a.s.l.], 15.vi.2002, 1 ♂, [at light (mercury-vapor lamp)], collector unknown, OBL det. + coll.; Pest County, Domonyvölgy, Bárányjárás, [47°37'23.8"N, 19°24'1.94"E, 220 m a.s.l.], 21.v.2004, 1 ♂, [at light (mercury-vapor lamp)], collector unknown, OBL det. + coll.

**Northern Hungary (Észak-Magyarország)**, Nógrád County, Kazár-Pólyos, ca 48°2'46.94"N, 19°52'32.37"E, ca 270 m a.s.l., June 2021, 1 ♀ crawling in the grass during the day, Viktória Szecskó obs. + photo, KHE det., DJP rev.

### ﻿Croatia

**Data from the internet**

**Baranja** [a microregion in northeastern Croatia, north of Osijek], locality not specified, June 2022, 1 ♂, BLZ obs. + photo (Association Hyla 2022).

### ﻿Serbia

**Published data**

**Vojvodina (Војводина)**, Srem District (Сремски округ), village of Vrdnik (Врдник) env., 45°07'31"N, 19°48'01"E, 235 m a.s.l., 8.–9.vi.2022, 5 spec. FSLG at 21.00–22.00 CEST, ABW, MBK and SET leg., coll. SGGW (Byk et al. 2023).

### ﻿Romania

**Published data**

**Transylvania (Transilvania)**, “Schässburg” [= Sighișoara or Segesvár], no other data, Karl Petri leg. (Petri 1910)—this is Petri’s first record of *B.unicornis* from Sighișoara; he then listed this locality once more for this species (Petri 1912).

### ﻿Belarus

**Material examined**

**Gomel Oblast (Гомельская вобласць)**, Karma District (Кармянскі раён), Karma (Карма) env., Karots’ki (Кароцькі), 20.vii.1987, 1 spec. crawling on a sandy steppe during daylight hours, MSC leg. + det., storage of the specimen unknown.

The species has been reported from Belarus by several authors (e.g., Chobot and Mourek 2007; Alonso-Zarazaga et al. 2013; Potocký and Majzlan 2015; Nuß and Jäger 2020; Hejda 2023), but no concrete records from this country have been published so far. The record above from the vicinity of the village of Karots’ki represents the first documented occurrence of *B.unicornis* for Belarus. This record, along with records from northeastern Ukraine (Vovk et al. 2005, 2016; Sheshurak et al. 2018, 2020, 2022; Kavurka et al. 2019), suggests the possibility of the species occurring in adjacent areas of Russia.

### ﻿Ukraine

**Published data**

**Chernihiv Oblast (Чернігівська область)**, Novhorod-Siverskyi Raion (Новгород-Сіверський район), Rozloty (Розльоти) env., 51°41'10.03"N, 33°8'30.37"E, [140 m a.s.l.], 31.vii.–1.viii.2021, 3 ♀♀, at light, M. V. Leshchenko leg. (Sheshurak et al. 2022).

The record from the vicinity of Rzhyshchiv in the Kiev Oblast (Juřena 2022) was published again by Vasyliuk (2022).

**Material examined**

“Полтав[ская] губ[ерния]” [= Poltava Governorate of the Russian Empire, a historical region of the Russian Empire located between 51°8' and 48°41'N and between 31°2' and 36°3'E], 1 ♂ (ex coll. M. K. Tikhonravov) with no other data, coll. ZMMU.

**Chernivtsi Oblast (Чернівецька область)**, Bukovina (Буковина), Chernivtsi Raion (Чернівецький район), “Bukowina, Czernowitz” [= Bukovina (Буковина), Chernivtsi (Чернівці)], 1 ♂ (ex coll. Josef [Giuseppe] Müller, 1880–1964), undated, coll. MNHT.

**Vinnytsia Oblast (Вінницька область)**, Vinnytsia Raion (Вінницький район), “Сквир[ский] у[езд] Киев[ской] г[убернии]” [= Kiev Governorate of the Russian Empire (disestablished 1925), Skvirsky Uyezd (incorrectly, it was actually Lipovetsky Uyezd), currently Vinnytsia Raion (Вінницький район)], “Ильинцы” [= Illintsi (Іллінці)], [ca 215 m a.s.l.], 14.vi.[year not specified], 2 ♂♂ (ex coll. M. K. Tikhonravov), A[ndrey] I[vanovich] Shelyuzhko [leg.], coll. ZMMU.

**Kyiv Oblast (Київська область)**, Kiyv (Київ), “Политехник” [= probably area of the National Technical University of Ukraine], July [19]26, 1 ♀ (ex coll. M. K. Tikhonravov), collector unknown, coll. ZMMU.

**Cherkasy Oblast (Черкаська область)**, Cherkasy Raion (Черкаський район), Kaniv (Канів) env., Kaniv Nature Reserve (Канівський природний заповідник), [49°43'12"N, 31°31'19"E, ca 200 m a.s.l.], 20.vi.1984, 1 ♂ [excavated from its burrow, steppe slope in a hornbeam forest], VGG leg., coll. ZMMU.

**Dnipropetrovsk Oblast (Дніпропетровська область)**, Dnipro Raion (Дніпровський район), Dnipro (Дніпро) [Dnipropetrovsk until 19 May 2016], Tunelna Balka tract (Тунельна балка) [the name of an area with oak forest in the southern part of the city], June 2009, 1 ♀, OSD leg., OBL det. + coll.; June 2011, 1 ♂, OSD leg., OBL det. + coll.; 48°25'02.7"N, 35°02'23.6"E, 100 m a.s.l., 8.vi.2014, 2 ♂♂ and 2 ♀♀, OSD leg., OBL det. + coll. (part of already published record—see Juřena 2022, specification of GPS and storage of part of specimens).
